# Age patterns of HIV incidence in eastern and southern Africa: a modelling analysis of observational population-based cohort studies

**DOI:** 10.1016/S2352-3018(21)00069-2

**Published:** 2021-06-28

**Authors:** Kathryn A Risher, Anne Cori, Georges Reniers, Milly Marston, Clara Calvert, Amelia Crampin, Tawanda Dadirai, Albert Dube, Simon Gregson, Kobus Herbst, Tom Lutalo, Louisa Moorhouse, Baltazar Mtenga, Dorean Nabukalu, Robert Newton, Alison J Price, Malebogo Tlhajoane, Jim Todd, Keith Tomlin, Mark Urassa, Alain Vandormael, Christophe Fraser, Emma Slaymaker, Jeffrey W Eaton, ALPHA Network

**Affiliations:** a MRC Centre for Global Infectious Disease Analysis, School of Public Health, Imperial College London, London, UK; b Department of Population Health, London School of Hygiene & Tropical Medicine, London, UK; c Medical Research Council/Wits University Rural Public Health and Health Transitions Research Unit (Agincourt), School of Public Health, Faculty of Health Sciences, University of the Witwatersrand, Johannesburg, South Africa; d Institute of Health and Wellbeing, University of Glasgow, Glasgow, UK; e The Manicaland Centre for Public Health Research, Harare, Zimbabwe; f Malawi Epidemiology and Intervention Research Unit, Karonga, Malawi; g Biomedical Research and Training Institute, Harare, Zimbabwe; h Africa Health Research Institute, KwaZulu-Natal, Durban, South Africa; i Department of Science and Innovation–Medical Research Council South African Population Research Infrastructure Network, Durban, South Africa; j Rakai Health Sciences Program, Kalisizo, Uganda; k National Institute for Medical Research, Kisesa HDSS, Mwanza, Tanzania; l Medical Research Council/Uganda Virus Research Institute and London School of Hygiene & Tropical Medicine Uganda Research Unit, Entebbe, Uganda; m Department of Health Sciences, University of York, York, UK; n KwaZulu-Natal Research Innovation and Sequencing Platform, UKZN, Durban, South Africa; o Heidelberg Institute of Global Health, Faculty of Medicine, University of Heidelberg, Heidelberg, Germany; p Oxford Big Data Institute, Li Ka Shing Centre for Health Information and Discovery, Nuffield Department of Medicine, University of Oxford, Oxford, UK

## Abstract

**Background:**

As the HIV epidemic in sub-Saharan Africa matures, evidence about the age distribution of new HIV infections and how this distribution has changed over the epidemic is needed to guide HIV prevention. We aimed to assess trends in age-specific HIV incidence in six population-based cohort studies in eastern and southern Africa, reporting changes in mean age at infection, age distribution of new infections, and birth cohort cumulative incidence.

**Methods:**

We used a Bayesian model to reconstruct age-specific HIV incidence from repeated observations of individuals' HIV serostatus and survival collected among population HIV cohorts in rural Malawi, South Africa, Tanzania, Uganda, and Zimbabwe, in a collaborative analysis of the ALPHA network. We modelled HIV incidence rates by age, time, and sex using smoothing splines functions. We estimated incidence trends separately by sex and study. We used estimated incidence and prevalence results for 2000–17, standardised to study population distribution, to estimate mean age at infection and proportion of new infections by age. We also estimated cumulative incidence (lifetime risk of infection) by birth cohort.

**Findings:**

Age-specific incidence declined at all ages, although the timing and pattern of decline varied by study. The mean age at infection was higher in men (cohort mean 27·8–34·6 years) than in women (24·8–29·6 years). Between 2000 and 2017, the mean age at infection per cohort increased slightly: 0·5 to 2·8 years among men and −0·2 to 2·5 years among women. Across studies, between 38% and 63% (cohort medians) of the infections in women were among those aged 15–24 years and between 30% and 63% of infections in men were in those aged 20–29 years. Lifetime risk of HIV declined for successive birth cohorts.

**Interpretation:**

HIV incidence declined in all age groups and shifted slightly to older ages. Disproportionate new HIV infections occur among women aged 15–24 years and men aged 20–29 years, supporting focused prevention in these groups. However, 40–60% of infections were outside these ages, emphasising the importance of providing appropriate HIV prevention to adults of all ages.

**Funding:**

Bill & Melinda Gates Foundation.

## Introduction

Eastern and southern Africa are disproportionately affected by HIV, with an estimated 54% of 38 million people living with HIV worldwide and 43% of new worldwide infections in 2019.^1^ The estimated annual number of new infections in this region decreased from 1·5 million in 1997 to 730 000 in 2019.^2^ However, this number still remains substantially higher than the UNAIDS 2020 target of 500 000 new infections among adults worldwide.^1^

Since the early years of the HIV epidemic, population-based cohort studies have been a key source of information about HIV epidemiology and trends in eastern and southern Africa.^3^ The ALPHA network began in 2005 as a collaboration of independently conducted population-based health and demographic surveillance systems in eastern and southern Africa that assess HIV status.^3^ Four cohort studies in the ALPHA network—Kisumu (Kenya),^4^ Manicaland (Zimbabwe),^5^ Rakai (Uganda),^6^ and uMkhanyakude (South Africa)^7^—and eight studies collectively^8^ have reported declines in the overall incidence of HIV in adults. Few studies, however, assessed changes in age-specific incidence in maturing HIV epidemics. A 2019 systematic review of data from ten countries in sub-Saharan Africa found that HIV incidence is decreasing in adolescent girls and young women (aged 15–24 years) in the general population, although the evidence to assess a trend was scarce.^9^

Combination HIV prevention interventions, including antiretroviral therapy (ART), medical male circumcision, condom use, and behaviour change, have been rolled out across eastern and southern Africa.^1^ HIV prevention initiatives increasingly aim to prioritise age groups at which most HIV infections occur. Most prominently, the Determined, Resilient, Empowered, AIDS-free, Mentored, and Safe (DREAMS) partnership, which is supported by the USA's President's Emergency Plan For AIDS Relief (PEPFAR) and was established in 2015, is a large-scale intervention focused on adolescent girls and young women in 15 countries in eastern and southern Africa.^10^ Data about age-specific HIV incidence and its trends over the epidemic provide an evidence base for interventions, insight about how combined interventions affect the age distribution of new infections, and guidance to optimise future prevention efforts.

**Research in context**

**Evidence before this study**

We searched PubMed, MEDLINE, and grey literature for English studies published between database inception and Nov 15, 2019 using the search terms “HIV”, “AIDS”, “incidence”, “age-specific”, “age patterns”, “proportion”, and “new infections”. We selected articles that reported on age-specific HIV incidence and proportion of HIV infections by age in eastern and southern Africa. Our search showed that several studies have reported HIV incidence in broad age categories (10–15 year age groups), though many estimates cover a single time period. A 2019 systematic review found evidence that HIV incidence is decreasing in adolescent girls and young women in eastern and southern Africa, but there was scarce evidence to assess a trend over time. UNAIDS estimates the proportion of new HIV infections in eastern and southern Africa among 15–24-year-olds to be 50% among women and 30% among men.

**Added value of this study**

Our analysis provides a detailed assessment of changes in age patterns of HIV incidence in six population-based cohort studies in eastern and southern Africa, using a Bayesian model that jointly estimates HIV incidence and mortality using serosurvey and vital status data. We found that age-specific incidence has decreased over time in these six studies. 38–63% of new infections among women were in 15–24-year-olds, and 30–63% of new infections in men were in 20–29-year-olds. The mean age at infection has started to increase in some studies as incidence has lowered, though changes were slight (−0·2 to 2·8 years). Although lifetime incidence has declined in most studies and sexes, substantial burden remains among women in uMkhanyakude, South Africa.

**Implications of all the available evidence**

As the HIV epidemic has matured, the relative contribution of different ages to new HIV infections has changed, supporting the importance of continuing to update HIV prevention strategies to reflect the changing disease burden. Age-targeting interventions for HIV prevention to adolescent girls and young women aged 15–24 years, as in the PEPFAR-supported DREAMS partnership, is supported by their high burden of HIV incidence, but variability between studies suggests that this is too narrow of a target in settings where age-specific incidence is less concentrated.

Most estimates of HIV incidence in population-based cohort studies rely solely on longitudinal observation of HIV seroconversion—ie, HIV-negative individuals who are prospectively followed up and re-tested for HIV. Given high levels of migration in and out of populations^11^, ^12^ and of non-participation in HIV testing,^13^ the reliance on individuals with a minimum of two test results discards substantial information about previous HIV incidence among individuals who are observed as HIV-positive upon enumeration into the cohort, and could lead to biased estimates of incidence over time. To incorporate data from all participants, we used a Bayesian model to jointly reconstruct age-specific HIV incidence and mortality using population-based HIV testing data and vital status information.

We aimed to assess trends in age-specific incidence over time in population-based cohort studies in Malawi, South Africa, Tanzania, Uganda, and Zimbabwe, reporting changes in mean age at infection, proportion of new infections in different age groups, and cumulative incidence in successive birth cohorts.

## Methods

### Data sources

The ALPHA network is comprised of ten ongoing population-based demographic surveillance studies across southern and eastern Africa that conduct HIV serosurveys.^3^ Six of these studies have collected demographic and HIV data on individuals aged 15 years and older since at least the early 2000s and are included in this analysis: Karonga (Malawi), uMkhanyakude (South Africa), Kisesa (Tanzania), Masaka and Rakai (Uganda), and Manicaland (Zimbabwe).

Each study conducts demographic surveillance involving enumeration of all household residents, births, deaths, and migrations in geographically defined populations. This is usually done via household visits at regular intervals. HIV serosurveys are done at different frequencies for each study (between annually and about every 3 years) and have different testing protocols and participation rates. Details of data collection and serological testing in each study are described elsewhere.^5^, ^14^, ^15^, ^16^, ^17^, ^18^

Participants from each of the included cohorts gave written informed consent to participate and for their data to be analysed by researchers within the organisation collecting the data and by their collaborators.^3^, ^5^, ^14^, ^15^, ^16^, ^17^, ^18^ The conduct of each longitudinal study is regularly reviewed by the relevant local ethics committee and the appropriate approvals were obtained. The London School of Hygiene & Tropical Medicine's ethics committee approved the pooled analysis of the secondary data.

Further details on the ALPHA network have been published previously.^3^

### Model description

We used a Bayesian model to simultaneously reconstruct age-specific incidence and mortality by sex in each cohort. By jointly modelling HIV incidence and survival after seroconversion, we incorporated data from study participants who were HIV-positive at first study visit or participated in only one serosurvey. Modelling the distribution of survival by age at infection enables these data to inform the timing and age of HIV seroconversion. These additional data increase the precision of age-specific incidence trends compared with typical cohort-based estimates of HIV incidence, which use only observations from participants with a HIV-negative test at baseline and a follow-up test.^19^

We jointly fit the model to individual records from demographic surveillance (whether an individual is living in the study area and, if yes, whether they are alive) and serosurveillance (HIV status if available). The model was specified by three hazard functions across time and age:

λ(t, a): HIV incidence rate at time t and age a;ϕ(t, a, u)=ω(a-u, u)·h_ART_(t): HIV-related death rate for time t, age a, and duration of infection u; μ(t, a): natural (non-HIV) mortality rate at time t and age a

We modelled HIV incidence hazard as a generalised additive model with an interaction between time and age,^20^ logλ(*t,a*)=*f*(*t*) + *f*(*a*) + *f*(*t,a*), where *f*(*t*) and *f*(*a*) specify average time and age pattern for incidence and the interaction *f*(*t,a*) allows for changes in the incidence age pattern over time. We modelled non-HIV mortality additively with average time and age patterns,^21^ log μ(*t,a*)=*g*(*t*) + *g*(*a*). Each of the terms of the generalised additive models were represented by penalised B-splines (p-splines) with knots every 5 years in age and time.^22^ The p-splines used are flexibly modelled splines that penalise first-order differences between adjacent coefficients to smooth between terms.

We modelled HIV survival by age at infection with a Weibull hazard function (ω*(a – u,u)*) based on age at infection *a–u* and time since infection *u*.^23^ HIV mortality hazard is modified by the ratio *h*_ART_(*t*) in the ART era (table), such that in pre-ART era, *h*_ART_(*t*)=1, and in the ART era, *h*_ART_(*t*) is estimated from the data on the basis of observed declines in the mortality of people living with HIV after ART became available. Diffuse prior distributions were specified for all spline coefficients and the variance on differences in spline coefficients; smoothness penalties were shared for men and women within each study, but spline coefficients were a priori conditionally independent between sexes.

**Table:** Data availability and model inputs for individuals aged ≥15 years, ALPHA network 1989–2017

	**Year demographic surveillance started**	**n**	**Person-years**	**Serosurveys**	**HIV-positive tests, %**	**Year ART started**
Karonga (Malawi)	2002	43 056	271 608	4	6%	2005
Kisesa (Tanzania)	1994	66 204	297 548	8	6%	2005
Manicaland (Zimbabwe)	1998	29 434	141 116	6	18%	2005
Masaka (Uganda)	1989	30 649	181 234	25	7%	2004
Rakai (Uganda)	1994	61 887	289 135	17	14%	2004
uMkhanyakude (South Africa)	2000	102 936	690 116	14	25%	2005

Data are for the survival cohort, except HIV-positive test data (ever collected). Additional details are given in the appendix (pp 14–19). ART=antiretroviral therapy.

The data for each individual consist of the dates first observed in demographic surveillance and last observed alive in the study or date of death, and a collection of HIV status observations from rounds of HIV serosurveys. HIV status observations defined an interval in which each individual could have seroconverted. For a specific seroconversion date in this interval, the likelihood for an observed individual is the probability of surviving to time last observed or died, conditional on having survived HIV-free to time of seroconversion and survived with HIV from seroconversion to time last observed or died. The model integrates over all possible seroconversion times from last HIV-negative observation to first HIV-positive observation. There are four cases of seroconversion intervals: (1) those HIV-positive at first observation (left-censored), for whom the seroconversion interval starts at age 10 years (start of the model); (2) those HIV-negative at last observation (right-censored) could seroconvert anytime from time last observed HIV-negative until last observation or death in demographic surveillance; (3) those with a HIV-negative and HIV-positive test (interval-censored) who seroconverted between these two tests; and (4) those with no HIV testing data who could have seroconverted anytime between first observation to last observation or death. By considering all four cases described, we used all HIV serostatus information to inform HIV incidence estimates, rather than exclusively using the cohort of seroconverters.

We reconstructed HIV incidence from the introduction of HIV in each study population, but since demographic surveillance and HIV serosurveillance only began several years into the epidemic, we added auxiliary information to specify an early prevalence of 0% before the HIV epidemic started in each study.^24^ For this auxiliary information, we inserted a hypothetical cross-sectional HIV serosurvey of 451 adults, aged 15–60 years by 0·1 of a year, with all HIV-negative individuals (year in appendix p 17).

For computation, the model and data were discretised to 0·2 year time-steps. We estimated the model using Hamiltonian Monte Carlo, No-U-Turn samplers in the Stan programming language (version 2.19).^25^ We ran four independent chains for 500 iterations, and combined the latter 250 iterations of chains to calculate summary statistics of the posterior distribution.

Additional model details are given in the appendix (pp 3–14).

### Statistical analysis

We estimated (1) age-specific incidence over time, (2) mean age at infection over time, (3) proportion of infections occurring within 5-year age groups, (4) narrowest age ranges that capture 25%, 50%, and 75% of new infections, and (5) cumulative incidence (lifetime risk of infection) by birth cohort.

For analyses of age distributions of new HIV infections, we applied modelled age-specific incidence rates and prevalence to the age structure of the resident population in the study areas enumerated through demographic surveillance (regardless of whether they participated in HIV serosurveys), with no smoothing between surveillance. Since Manicaland does not conduct continuous demographic surveillance, we standardised to the national population.^26^ Modelled HIV prevalence was applied to produce estimates of the population susceptible by time and age, to which we applied incidence hazards to calculate new infections by age. Mean age at infection was weighted by the population susceptible in each age group *w*_t,a_ at each time *t:*

$$\left(\frac{\sum_{a}\lambda (t,a).a.w_{t,a}}{\sum_{a}\lambda (t,a).w_{t,a}}\right)$$

Cumulative incidence by birth cohort was calculated by cumulating the hazard across age-specific and time-specific incidence estimates by birth year:1 – exp(−*t*·Σ_(t,a)_ λ*(t,a)*).

We projected future cumulative incidence by birth cohort under two assumptions: (1) future age-specific incidence rates remain constant at the most recent estimated levels (2012 for Karonga and Manicaland, 2017 for uMkhanyakude, Rakai, Masaka, and Kisesa), and (2) age-specific HIV incidence rates continue to decline at the same study-specific and sex-specific rate estimated for the past 5 years among ages 15–54 years. Sex differences in the rate of incidence decline are reduced from the end of the data period, converging in 2022 after which the rate of decline is assumed to be the same in both sexes (appendix pp 31–32).

For each outcome, we present results for 2000–17 or end of the most recent HIV serosurveillance in each study (2012 in Karonga and Manicaland) for individuals aged 15–54 years due to limited serosurveillance in those younger than 15 years and older than 54 years. Results represent the median of 1000 posterior samples, with 95% credible intervals (95% CrIs) representing the 2·5th and 97·5th percentiles of these distributions. We used R statistical software (version 3.6) to analyse modelled outputs. Owing to substantial variability in the magnitude of HIV epidemics in the different studies, plotted results are presented side-by-side with different vertical axis ranges.

Analyses assessing sensitivity to assumptions about the type of HIV incidence hazard smoothing term, the population standardised to, and comparisons of modelled outputs to direct estimates from seroconverter cohorts are shown in the appendix (pp 34–53).

### Role of the funding source

The funder of the study had no role in study design, data collection, data analysis, data interpretation, or writing of the report.

## Results

**Figure 1:**
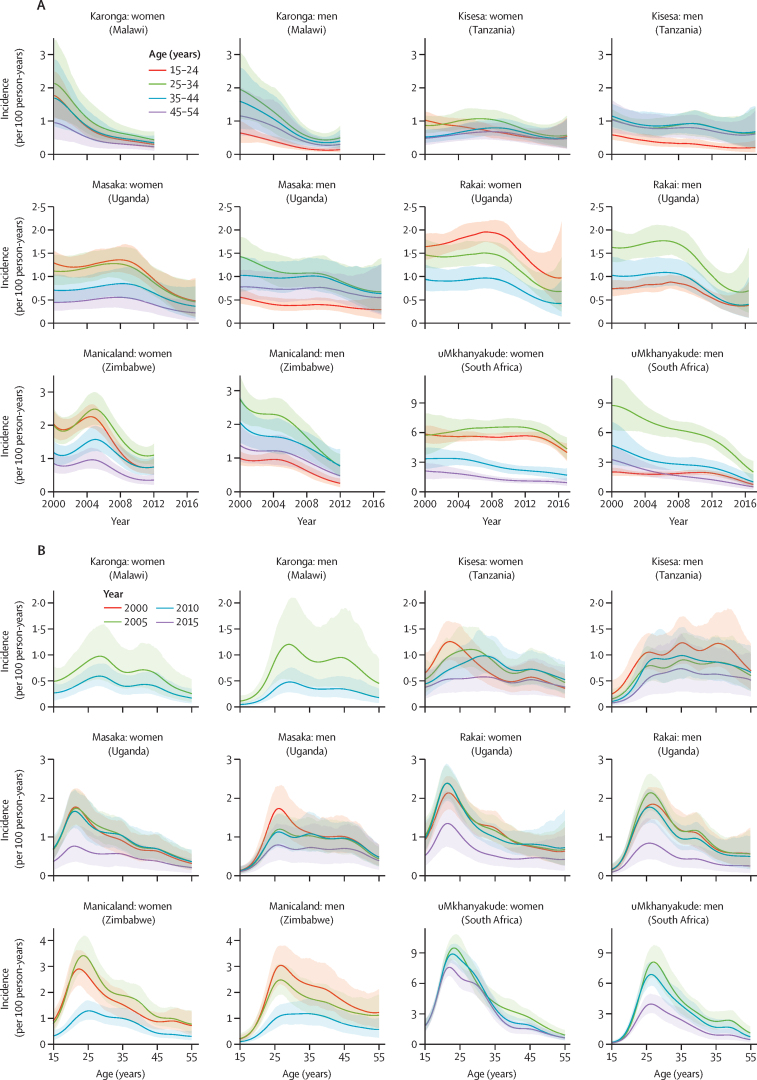
Age-specific HIV incidence modelled estimates in six ALPHA network studies by age group (A) and by year (B) (A) Rakai does not include a line for age 45–54 years because the cohort has largely not done HIV tests in individuals older than 49 years. (B) Karonga, Manicaland, and uMkhanyakude do not include results for 2000 and 2015, 2015, and 2000, respectively, due to different start and end dates of HIV testing rounds. More details on serosurvey years are given in the appendix (p 17).

Age-specific incidence decreased over time across most age groups in these studies, though decreases were observed at different times (figure 1A). In 2000 and 2005, HIV incidence generally peaked between ages 20–24 years for women and 25–29 years for men (figure 1B). Over time, age-specific incidence flattened in Karonga, Kisesa, Manicaland, and Masaka. In Rakai and uMkhanyakude, the peaked incidence age pattern did not change. Recent incidence declines among women in uMkhanyakude were greatest below age 30 years. HIV prevalence trends by age group are shown in the appendix (p 29).

**Figure 2:**
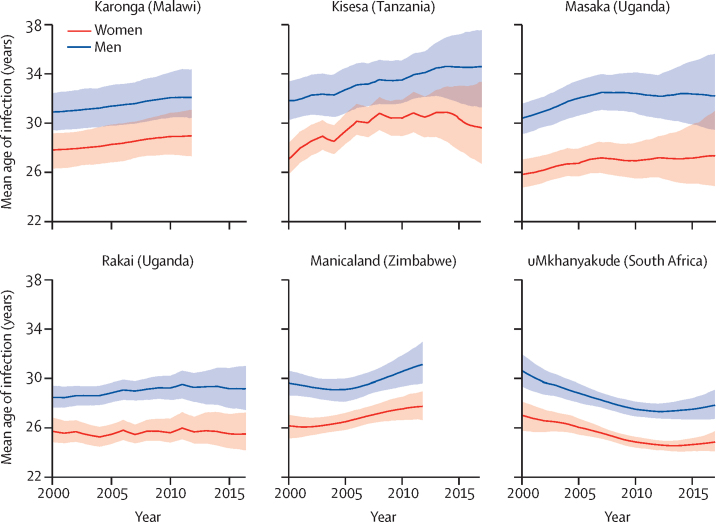
Mean age at infection in six ALPHA network studies by sex

The mean age at infection was higher in men than women in all studies, and the difference between the sexes remained similar over time (figure 2). In the most recent estimate (2012 for Karonga and Manicaland, 2017 for elsewhere), mean age at infection among men was 27·8 years (95% CrI 26·7–29·1) in uMkhanyakude, 29·2 (27·5–31·0) in Rakai, 31·1 (29·6–33·0) in Manicaland, 32·1 (30·4–34·4) in Karonga, 32·2 (29·5–35·6) in Masaka, and 34·6 (31·3–37·5) in Kisesa. Among women, most recent mean age at infection was 24·8 years (95% CrI 24·1–25·7) in uMkhanyakude, 25·5 (24·2–27·2) in Rakai, 27·3 (24·9–31·0) in Masaka, 27·7 (26·6–29·0) in Manicaland, 29·0 (27·3–31·1) in Karonga, and 29·6 (26·7–33·3) in Kisesa. The mean age at infection increased slightly from 2000 to most recent estimate in Kisesa (2·8 years in men, 2·5 in women), Masaka (1·8 years in men, 1·5 in women), Manicaland (1·5 years in men, 1·6 in women), Karonga (1·2 years in men, 1·2 in women), and Rakai men (0·7 years); women in Rakai showed little change (−0·2 years). Mean age at infection decreased between 2000 and 2017 in uMkhanyakude (−2·8 years in men, −2·2 in women), although the mean age at infection increased slightly between 2013 and 2017 (0·5 years in men, 0·3 in women).

**Figure 3:**
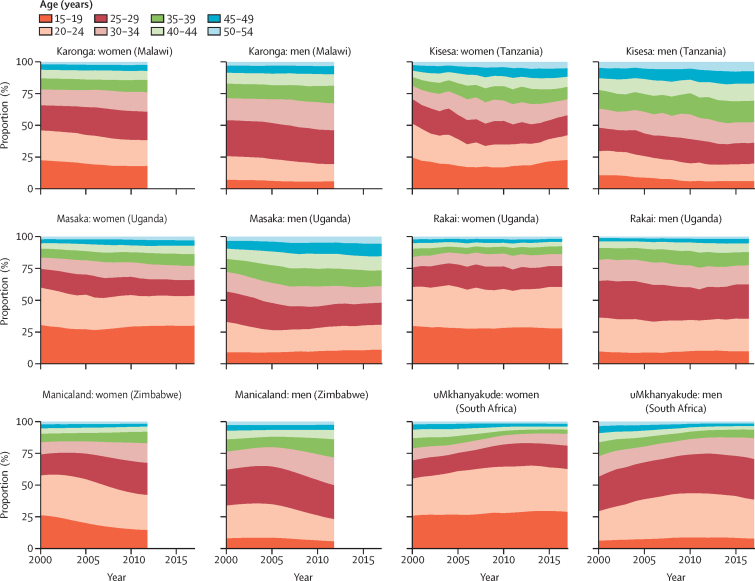
Proportion of new HIV infections in six ALPHA network studies by age group

The proportions of new infections occurring in each 5-year age group was relatively stable over time and broadly similar across studies (figure 3). In all studies, for those aged 15–19 years, a higher proportion of infections occur among women (14–29%) than men (5–11%), whereas men have a greater proportion of infections among those aged 25–34 years (23–40% among women and 30–48% among men). In Kisesa, age at infection was more evenly distributed across ages than in other studies.

38–63% (cohort medians) of the infections in women were among those aged 15–24 years (figure 3). This value was above 50% in the most recent year for three of six studies: uMkhanyakude (63%, 95% CrI 59–67), Rakai (60%, 52–69), and Masaka (53%, 37–66). In the other three studies, this proportion was below 50%: Karonga (38%, 28–46), Kisesa (41%, 27–55), and Manicaland (42%, 35–49). Among men in the most recent year, while 15–24-year-olds represented 19–39% of infections in men, men aged 20–29 years represented more than 50% of infections in uMkhanyakude (63%, 54–69) and Rakai (52%, 43–61), and less than 50% in Manicaland (44%, 32–54), Karonga (40%, 30–49), Masaka (36%, 25–47), and Kisesa (30%, 18–43). The uncertainty ranges for these estimates show substantial uncertainty around the proportion of infections attributable to each age group. Uncertainty was largest in the youngest age group and in smaller studies.

**Figure 4:**
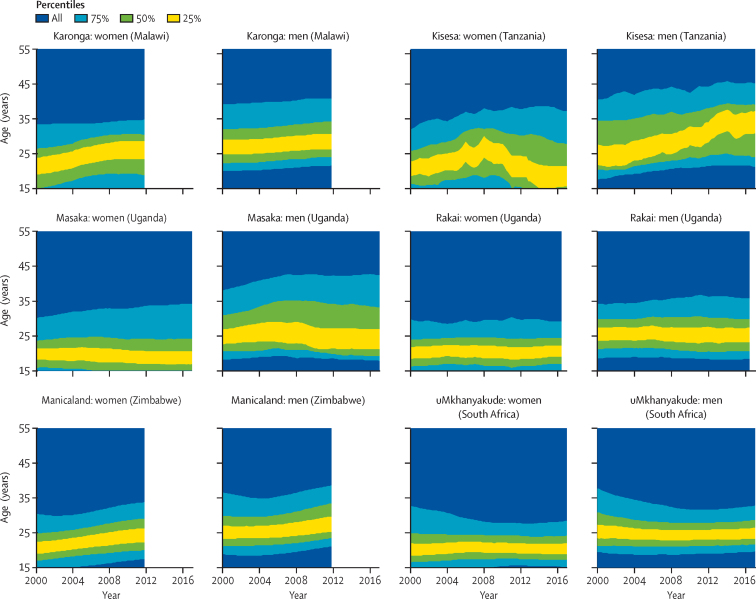
Narrowest age bands accounting for 25%, 50%, and 75% of new HIV infections in six ALPHA network studies

Across studies, the width of the age range within which 75% of new infections occurred in the most recent year was 13·2–22·2 years for women and 13·2–24·6 years for men (figure 4). For women in Rakai and uMkhanyakude, the narrowest band to capture 75% of infections lies, in most years, between ages 15 years and 30 years, the most recent estimate is 15·0–29·2 years in Rakai and 15·2–28·4 years in uMkhanyakude. Among women in Karonga, Kisesa, Manicaland, and Masaka, this 75% band extends slightly older at 15–35 years, with some changes over time in Manicaland and Masaka as infections in women have shifted to older ages. Most recent estimates are 15·0–34·8 years in Karonga, 15·0–37·2 years in Kisesa, 17·4–33·8 years in Manicaland, and 15·0–34·2 years in Masaka.

Among men in the most recent estimates, the narrowest band to capture 75% of infections is in ages 20–35 years in Rakai and uMkhanyakude (most recent estimates are 18·4–35·8 years in Rakai and 19·6–32·8 years in uMkhanyakude), closer to ages 20–40 years in Karonga and Manicaland (most recent estimates are 21·6–40·8 years in Karonga and 21·0–38·6 years in Manicaland), and somewhat wider in Kisesa and Masaka (most recent estimates are 21·2–45·2 years in Kisesa and 17·8–42·4 years in Masaka; figure 4).

**Figure 5:**
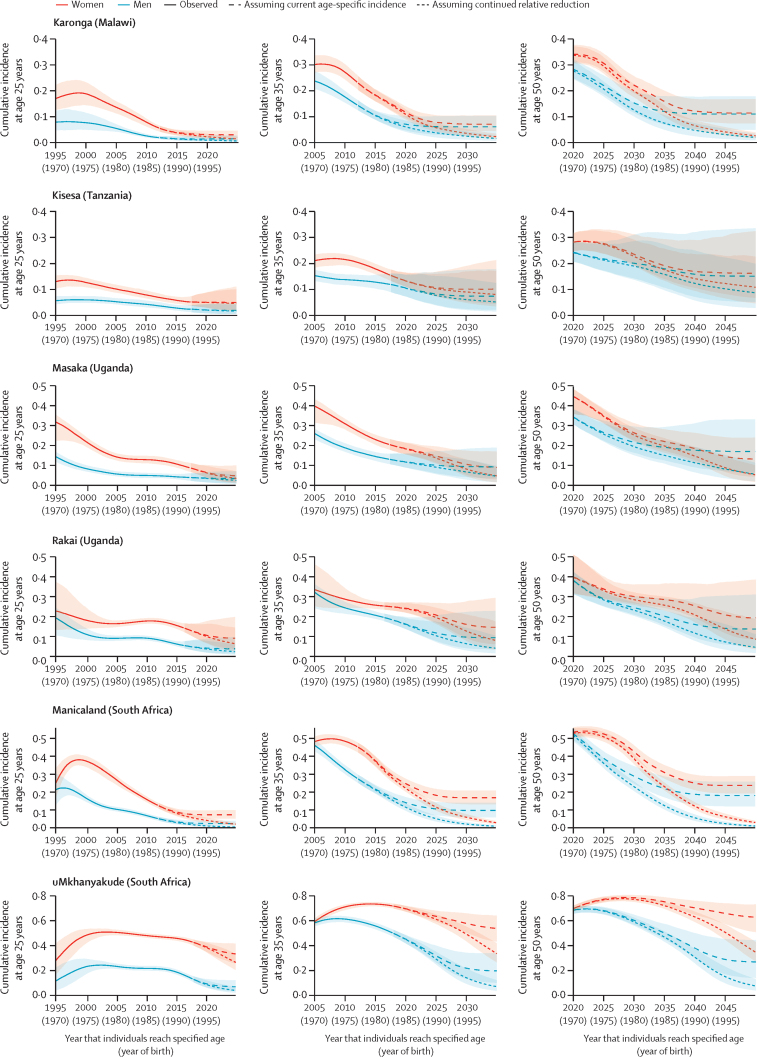
Cumulative incidence of HIV at ages 25, 35, and 50 years by birth cohort in six ALPHA network studies by sex Data are cumulative incidence of HIV at the specified ages, which were projected assuming current period age-specific incidence and assuming a continuation of the relative reduction seen in the past 5 years.

The lifetime risk of HIV infection, measured by cumulative incidence of HIV infection between ages 15 years and 49 years, has declined for more recent birth cohorts in all studies; however, declines in women in uMkhanyakude occur later than for all other studies and sexes (figure 5). For the birth cohort turning 50 years old in 2020, the percentage of women who were infected with HIV (either deceased or surviving) was 28% (95% CrI 25–32) in Kisesa, 34% (31–38) in Karonga, 40% (32–51) in Rakai, 45% (36–48) in Masaka, 53% (51–56) in Manicaland, and 70% (67–72) in uMkhanyakude. In uMkhanyakude, we projected that cumulative incidence will peak for the cohort turning 50 years old in 2029 at 79% (77–81). For men, the cumulative incidence for the cohort turning 50 years old in 2020 was similar to women in the same population—ranging from 24% (21–28) in Kisesa to 53% (49–56) in Manicaland and 69% (66–71) in uMkhanyakude. By contrast, HIV prevalence at the most recent estimate among 45–54 year olds in each study was lower in both sexes, at 14–26% in Kisesa, Karonga, Masaka, and Manicaland and 45–48% in uMkhanyakude (appendix p 29).

If incidence of HIV were to remain constant at 2017 levels, by 2050, the cohort of women turning 50 years old will have cumulative incidence from 11% (95% CrI 7–17) in Karonga to 24% (19–29) in Manicaland and 63% (51–73) in uMkhanyakude, representing relative reductions of 10–70% from 2020 levels. Notably, among women in uMkhanyakude, cumulative incidence at age 20 would be 13·5% when assuming constant incidence at 2017 levels. For men, birth cohort cumulative incidence at age 50 in 2050 will range from 11% (5–18) in Karonga to 18% (12–26) in Manicaland and 27% (14–44) in uMkhanyakude, a reduction of 36–65% since 2020.

If incidence continues to decline at the rate observed in the past 5 years in both sexes, the cohort turning 50 years old in 2050 will have a median risk of infection of less than 12% in all studies except uMkhanyakude, where the projected cumulative incidence will reduce to 34% (95% CrI 27–43) for women and 7% (4–13) for men.

## Discussion

Our modelling analysis showed that age-specific incidence of HIV has declined over time in all six population-based cohort studies, although the magnitude and timing of this decline varied across studies and between sexes. In studies with lower current epidemic levels and earlier declines in incidence—Karonga (Malawi), Kisesa (Tanzania), Manicaland (Zimbabwe), and Masaka (Uganda)—the age pattern of incidence flattened across ages over time and the mean age at infection was slightly older than in other studies and increased since 2000. In Rakai (Uganda) and uMkhanyakude (South Africa), where incidence until 2008 and 2012, respectively, was somewhat higher but has declined rapidly, the distribution of age at infection was younger and the historical peak of age-specific incidence was maintained. Early analyses of the most recent HIV serosurvey in Rakai reported extremely low incidence in young women (0·21 per 100 person-years among women aged 15–24 years), suggesting that Rakai might also be shifting into the first grouping of studies.^27^

In the past 5 years, HIV prevention strategies have increasingly focused on prioritising HIV prevention to age groups at the highest risk of HIV. The PEPFAR DREAMS partnership and South Africa's She Conquers initiative have made large investments in HIV prevention and risk reduction focused on adolescent girls and young women.^10^ Some stakeholders have raised concerns that these approaches neglect women aged 25–34 years with high HIV incidence rates, and they encourage the provision of HIV prevention for women of all ages.^28^ For men, questions persist about the short-term and long-term value of promoting voluntary medical male circumcision among different age groups.^29^

Our findings underscore the importance of providing effective HIV prevention for adults at risk of HIV across all ages. While there was some variation across study populations in the age distribution of new infections, 38–63% of current new infections among women are among those aged 15–24 years, and 14–29% among those aged 15–19 years. These data highlight the efficiency of focusing large-scale HIV primary prevention on adolescent girls and young women. However, such strategies in isolation would exclude roughly 40–60% of infections occurring among women older than 25 years. Other research has shown that women who are widowed or divorced, in serodiscordant partnerships, engaged in high-risk activity (such as sex work or transactional sex), or living in locations with high HIV prevalence and unsuppressed viral load, are at increased risk for acquiring HIV,^1^ which might focus optimal HIV prevention provision for these ages.

Our estimates of the age distribution of new infections from population-based cohort studies were broadly consistent with UNAIDS estimates for eastern and southern Africa, which have guided age-targeted intervention strategies. UNAIDS reported that, in 2019, 50% of new infections in adult women and 30% in adult men were among those aged 15–24 years,^1^ compared with 38–63% for women aged 15–24 years and 19–39% for men aged 15–24 years across our study populations.

The mean age at infection increased slightly in most studies by 0·7 to 2·8 years since 2000, which might reflect both natural HIV epidemic dynamics and interventions focused on young adults. In uMkhanyakude, the population with the largest epidemic and in which incidence peaked and declined most recently of the populations studied here, we estimated that the mean age at infection decreased throughout the 2000s but increased from 2013, later than other study populations. These findings are consistent with South Africa's later HIV epidemic, with previous modelling work showing that mean age at infection would decrease through the 2000s due to saturation of infections among older ages.^30^ The older mean age at infection in populations with lower overall incidence is consistent with foundational findings from infectious disease dynamics; for example, the relationship between force of infection and age at infection for vaccine preventable childhood infections.^31^ Although simple formulaic relationships do not directly apply to highly structured age patterns of HIV risk, heterogeneity in mixing, and complex interventions, the disease dynamics perspective could help to guide optimal interventions across epidemic settings into the future.

Although HIV prevalence in adults aged 45–54 years peaked at 14–26% in most studies and at 45–48% in uMkhanyakude, the cumulative effect of the epidemic on birth cohorts passing through adulthood during the late 1990s and early 2000s was much more severe. 28–53% of the birth cohorts turning 50 years old in 2020, excluding uMkhanyakude, ultimately became infected with HIV. In uMkhanyakude, up to 70% (95% CrI 67–72) of women were infected in the most affected cohorts to date, which is anticipated to increase to 79% (77–81) for women turning 50 years old in 2029. Although HIV point prevalence is lower for men than for women, the peak cumulative proportion infected was similar between sexes. However, we project that, in uMkhanyakude, the female:male ratio in cumulative incidence at 50 years old will increase over time. Lifetime risk of infection decreased substantially for more recent birth cohorts. In the absence of a reversal of these trends, our findings support projections that the number of young people living with HIV will decrease over time.^32^ However, further reductions in incidence are needed to control HIV transmission, particularly in uMkhanyakude where estimated age-specific incidence rates still resulted in 13·5% cumulative incidence by age 20 among adolescent girls and young women when assuming constant incidence at 2017 levels.

Our study has some limitations. First, the geographically localised nature of these population-based cohort studies, which are mostly rural populations under surveillance for long periods that were initially selected as highly HIV-affected populations, limits the generalisability of our findings. However, the consistency of age-specific incidence dynamics seen across these populations gives us some confidence that our conclusions are likely to be similar to other southern and eastern African populations. Health and demographic surveillance in urban populations in sub-Saharan Africa are being expanded^33^ and will provide vital resources for understanding incidence patterns in urban areas. Second, the six studies we included did not systematically include HIV testing for individuals younger than 15 years, so trends in younger ages were not characterised here. Third, our cumulative incidence projections by birth cohort reflect two simplified assumptions. Mechanistic modelling could provide improved projections of future incidence trends. Finally, our analyses only assess HIV acquisition, instead of risk of transmission, and can thus only provide half of the information to guide interventions for HIV prevention. Phylogenetic studies have helped to inform who is transmitting the virus.^34^

Decreases in age-specific incidence across almost all ages suggest that the HIV epidemic in these study populations has contracted substantially. Successive birth cohorts entering adulthood in the 2000s have experienced substantially lowered lifetime probability of HIV infection. Our findings emphasise the importance of providing appropriate HIV prevention to adults across all age ranges and continuing to adapt interventions to different contexts as infections shift over time.

## Data sharing

The ALPHA network dataset presented here represents harmonised data from independently conducted longitudinal population-based cohort studies. The pooled ALPHA network dataset is maintained by the London School of Hygiene & Tropical Medicine (https://alpha.lshtm.ac.uk/), but access is conditional on receiving approval from each study to analyse the data. Contact details for the study sites are in the appendix (p 20).

## Declaration of interests

SG, MT, ES, and JWE report grants from the Gates Foundation. SG also reports grants from the Wellcome Trust, UK Medical Research Council/Department for International Development, US National Institutes of Health, and WHO, and dividends on ordinary shares in AstraZeneca and GlaxoSmithKline, outside of the submitted work. JWE also reports grants from the UNAIDS, US NIH, and WHO, and personal fees from WHO. LM reports grants from the London School of Hygiene & Tropical Medicine. All other authors declare no competing interests.
