# Supplementary material for: Age patterns of HIV incidence in eastern and southern Africa: a modelling analysis of observational population-based cohort studies
Source: Lancet HIV. 2021 Jun 28;8(7):e429–39. doi: 10.1016/S2352-3018(21)00069-2 (PMC8258368; doi:10.1016/S2352-3018(21)00069-2)
Supplement: Supplementary appendix [file mmc1.pdf]

THE LANCET
HIV

Supplementary appendix

This appendix formed part of the original submission and has been peer reviewed. We post it as supplied by the authors.

Supplement to: Risher KA, Cori A, Reniers G, et al. Age patterns of HIV incidence in eastern and southern Africa: a modelling analysis of observational population-based cohort studies. *Lancet HIV* 2021; **8:** 429–39.

Age patterns of HIV incidence in eastern and southern Africa: a modelling analysis of observational population-based cohort studies — Supplementary appendix

Table of Contents

S1 Supplemental Methods ... *3*

S1.1 Likelihood details ... **3**

S1.2 Specification of hazard functions ... **7**

HIV incidence hazard $\lambda_{\theta_\lambda}(t, a)$ ... 8

Non-HIV mortality hazard $\mu_{\theta_\mu}(t, a)$ ... 10

Excess HIV mortality hazard $\rho_{\theta_\rho}(t, a, u)$ ... 11

S1.3 Model implementation ... **13**

S1.4 Population under demographic surveillance ... **14**

S1.5 HIV serosurvey data by study ... **17**

S1.6 Data sharing ... **20**

S2 Supplemental Model Outputs ... *20*

S2.1 Data to reproduce main figures ... **20**

S2.2 HIV trends among age 15-54 ... **20**

S2.3 Estimate for proportion HIV positive upon entry to population. ... **23**

S2.4 Age distribution of HIV infections ... **24**

S2.5 HIV incidence trend by single-year age ... **26**

S2.6 HIV prevalence by age ... **29**

S2.7 Cumulative HIV incidence by birth cohort ... **30**

S2.8 Sex ratio in incidence projections **31**

S2.9 Population pyramid of HIV population **32**

S3 Comparison to Direct Estimates ***34***

S3.1 Age-specific HIV prevalence by serosurvey round **34**

S3.2 HIV incidence rate and cumulative incidence 15-54 .... **43**

S3.3 Mean age of HIV infection **45**

S4 Sensitivity Analyses ***46***

S4.1 National reference populations **46**

S4.2 Alternative specifications for $\tilde{\lambda}(t, a)$ **50**

S5 STROBE statement ***54***

References ***57***

S1 Supplemental Methods

S1.1 Likelihood details

The data for each participant consists of (A) survivorship episodes recorded through longitudinal demographic surveillance and (B) a collection of HIV status observations resulting from HIV tests conducted during cross-sectional HIV sero-surveillance rounds during the period (Figure S1).

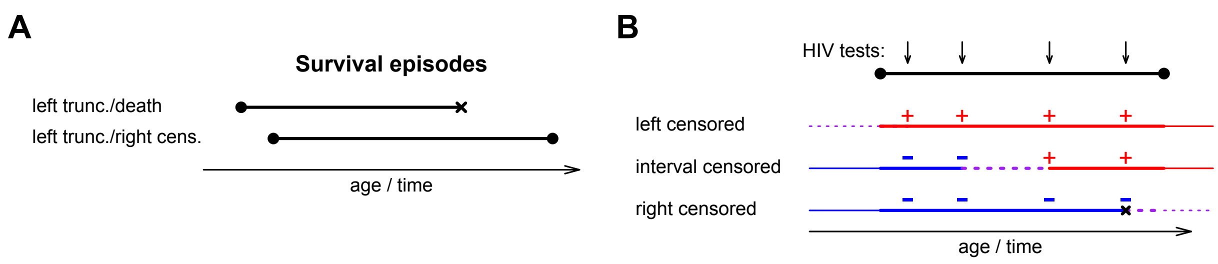

Figure S1: Summary of data available for each individual participating in population cohort study.

The relevant data for an individual i of a given sex in a given study can be summarised by a vector:

$$Y_i = \{t_i^B, t_i^F, t_i^E, \delta_i, t_i^N, t_i^P, \gamma_i\}$$

where

- t_i^B = date of birth,
- t_i^F = date of entry into cohort (first observed alive),
- t_i^E = date of exit from cohort either due to death or right censoring,
- δ_i = indicator variable indicating death (=1) or censoring (=0) at t_i^E,
- t_i^N = earliest date when the individual could have HIV seroconverted (either the date of last HIV-negative test or age 10),
- t_i^P = latest date when the individual could have HIV seroconverted (either the date of the first HIV-positive test or t_i^E),

- γ_i = indicator of whether the person was known HIV-positive (=1) or censored (=0) at t_i^P.

Survival episodes are left truncated and start (t_i^F) on the date when an individual was first observed in the study population (Figure S1A). Survival episodes end (t_i^E) either on the observed date of death ($\delta_i = 1$) or when the person was last observed alive in the population (right-censored observations; $\delta_i = 0$).

The collection of HIV status observations define an interval in which each person could have seroconverted, classified as one of the following cases (Figure S1B):

1) **Left-censored:** the individual was HIV positive at the first HIV test, indicating seroconversion happened sometime before the first observation. For these individuals, $t_i^N = t_i^B + 10$, t_i^P is the date of first HIV-positive test, and $\gamma_i = 1$.
2) **Interval-censored:** the individual has an observed HIV-negative test and a subsequent HIV positive test. t_i^N is the date of last HIV-negative test, t_i^P is the date of first HIV-positive test, and $\gamma_i = 1$.
3) **Right-censored:** the most recent HIV test was HIV-negative, but the individual may have seroconverted after the last sero-survey. t_i^N is the date of last HIV-negative test, t_i^P is the date of censoring or death (t_i^E), and $\gamma_i = 0$.
4) **No HIV data** (not illustrated in Figure S1B): these individuals could have seroconverted anytime from age 10 to last observed/death, or never seroconverted. For this case, $t_i^N = t_i^B + 10$, $t_i^P = t_i^E$, and $\gamma_i = 0$.

The likelihood for Y_i is expressed in terms of three hazard functions:

- $\lambda_{\theta_\lambda}(t, a)$: the HIV incidence rate at time t and age a,
- $\mu_{\theta_\mu}(t, a)$: the non-HIV mortality rate at time t and age a, and

- $\rho_{\theta_\rho}(t, a, u)$: the excess mortality rate due to HIV for a person with duration of infection u at time t and age a.

In the above θ_λ, θ_μ, and θ_ρ denote a generic collection of parameters determining the respective hazard functions—the spline coefficients and corresponding hyper-parameters. These terms are further defined below, but for brevity are omitted from the notation in subsequent description of the likelihood.

The following survival functions are defined based on these hazards:

- $\Lambda(t, a) = e^{-\int_0^a \lambda(t-\tau, a-\tau)\, d\tau}$: the probability that a person aged a at time t escapes HIV infection from birth to time t,
- $\Phi(t, a) = e^{-\int_0^a \mu(t-\tau, a-\tau)\, d\tau}$: the probability that an HIV-negative person aged a at time t survives from birth to time t (*i.e.* escapes death from non-HIV causes), and
- $\Psi(t, a, u) = e^{-\int_0^u \rho(t-\tau, a-\tau, \tau)\, d\tau}$: the probability that an individual infected at time $t - u$ avoids HIV death for duration u (up to time t and age a).

The total mortality hazard for HIV infected individuals is $\rho(t, a, u) + \mu(t, a)$, the sum of the excess mortality due to HIV and the hazard of non-HIV mortality. The probability of surviving (avoiding HIV and non-HIV death) from infection at time $t - u$ to time t and age a may be conveniently factored as:

$$
\begin{aligned}
e^{-\int_0^u (\rho(t-\tau, a-\tau, \tau) + \mu(t-\tau, a-\tau))\, d\tau} &= e^{-\int_0^u \rho(t-\tau, a-\tau, \tau)\, d\tau} \times e^{-\int_0^u \mu(t-\tau, a-\tau)\, d\tau} \\
&= e^{-\int_0^u \rho(t-\tau, a-\tau, \tau)\, d\tau} \times e^{-[\int_0^a \mu(t-\tau, a-\tau)\, d\tau - \int_0^{a-u} \mu(t-\tau, a-\tau)\, d\tau]} \\
&= \Psi(t, a, u) \times \frac{\Phi(t, a)}{\Phi(t-u, a-u)}.
\end{aligned}
$$

To develop the likelihood for an individual Y_i, initially leave aside left truncation of survivorship data (that is assume $t_i^F = t_i^B + 10 = t_i^{F_0}$).

First, consider the case of a person who is known to have seroconverted, that is $\gamma_i = 1$. Given a known date of seroconversion $s \in \{t_i^N, t_i^P\}$, the probability of Y_i is

$$P(Y_i|\boldsymbol{\theta}, t_i^{F_0}, s) = P(\text{Escaped infection and death up to } s) \times P(\text{Infected at } s)$$

$$\times P(\text{Survived up to } t_i^E \mid \text{Infected at } s) \times P(\text{Died at } t_i^E \mid \text{Infected at } s)^{\delta_i}$$

$$= \Lambda(s, s - t_i^B) \cdot \Phi(s, s - t_i^B) \cdot \lambda(s, s - t_i^B) \cdot \Psi(t_i^E, t_i^E - t_i^B, t_i^E - s)$$

$$\cdot \frac{\Phi(t_i^E, t_i^E - t_i^B)}{\Phi(s, s - t_i^B)} \cdot \left(\rho(t_i^E, t_i^E - t_i^B, t_i^E - s) + \mu(t_i^E, t_i^E - t_i^B)\right)^{\delta_i}$$

$$= \Phi(t_i^E, t_i^E - t_i^B) \cdot \Lambda(s, s - t_i^B) \cdot \lambda(s, s - t_i^B) \cdot \Psi(t_i^E, t_i^E - t_i^B, t_i^E - s) \cdot$$

$$\cdot \left(\rho(t_i^E, t_i^E - t_i^B, t_i^E - s) + \mu(t_i^E, t_i^E - t_i^B)\right)^{\delta_i}$$

Integrating over all possible seroconversion dates $s \in \{t_i^N, t_i^P\}$ gives the likelihood:

$$P(Y_i|\boldsymbol{\theta}, t_i^{F_0}, s \leq t_i^E) = \int_{t_i^N}^{t_i^P} P(Y_i|\boldsymbol{\theta}, t_i^{F_0}, s)\, ds$$

For the case $\gamma_i = 0$, individual i was not observed HIV+. The likelihood can be expressed as the sum of the probabilities that the individual seroconverted in the interval $s \in \{t_i^N, t_i^P = t_i^E\}$ and the probability of avoiding HIV infection to time t_i^E:

$$P(Y_i|\boldsymbol{\theta}, t_i^{F_0}) = P(Y_i|\boldsymbol{\theta}, t_i^{F_0}, s \leq t_i^E) + P(Y_i|\boldsymbol{\theta}, t_i^{F_0}, s > t_i^E)$$

The case $P(Y_i|\boldsymbol{\theta}, t_i^{F_0}, s \leq t_i^E)$ is as defined above. The probability of survival to t_i^E without infection is

$$P(Y_i|\boldsymbol{\theta}, t_i^{F_0}, s > t_i^E) = P(\text{Escaped infection and death up to } t_i^E) \times P(\text{Died at } t_i^E)^{\delta_i}$$

$$= \Lambda(t_i^E, t_i^E - t_i^B) \cdot \Phi(t_i^E, t_i^E - t_i^B) \cdot \mu(t_i^E, t_i^E - t_i^B)^{\delta_i}.$$

To handle the cases $\gamma_i = 1$ and $\gamma_i = 0$ simultaneously, the likelihood contribution may be expressed as

$$P(Y_i|\boldsymbol{\theta}, t_i^{F_0}) = P(Y_i|\boldsymbol{\theta}, t_i^{F_0}, s \leq t_i^E) + (1 - \gamma_i) \cdot P(Y_i|\boldsymbol{\theta}, t_i^{F_0}, s > t_i^E).$$

The assumption that $t_i^F = t_i^{F_0}$ is relaxed by accounting for left truncation by dividing by the probability of surviving from $t_i^{F_0}$ to t_i^F:

$$P(Y_i|\boldsymbol{\theta}) = \frac{1}{P(\text{Survive to } t_i^F)} \cdot P\left(Y_i \middle| \boldsymbol{\theta}, t_i^{F_0}\right)$$

where

$$\begin{aligned}
&P\left(\text{Survive to } t_i^F\right) \\
&\qquad = P\left(\text{Escape infection and death to } t_i^F\right) \\
&\qquad + \int_{t_i^B+10}^{t_i^F} P(\text{Escaped infection and death to } s) \times P(\text{Infected at } s) \\
&\qquad \times P\left(\text{Survived up to } t_i^F \;\middle|\; \text{Infected at } s\right) ds \\
&\qquad = \Phi\left(t_i^F, t_i^F - t_i^B\right) \\
&\qquad \cdot \left(\Lambda\left(t_i^F, t_i^F - t_i^B\right) + \int_{t_i^B+10}^{t_i^F} \Lambda\left(s, s - t_i^B\right) \cdot \lambda\left(s, s - t_i^B\right) \cdot \Psi\left(t_i^F, t_i^F - t_i^B, t_i^F - s\right) ds\right)
\end{aligned}$$

Finally, the likelihood for all participants $i = 1, \dots, N_C$ of sex and study cohort C is the product of the individual likelihood contributions:

$$L\left(\boldsymbol{\theta} \middle| Y_1, \dots, Y_{N_C}\right) = \prod_{i=1}^{N_C} P(Y_i|\boldsymbol{\theta}).$$

S1.2 Specification of hazard functions

The three hazard functions $\lambda_{\theta_\lambda}(t, a)$, $\mu_{\theta_\mu}(t, a)$, and $\rho_{\theta_\rho}(t, a, u)$ were defined on the time range $t \in [t_0, 2018]$ and age range $a \in [10, 100)$ years. Time t_0 is the first year in which HIV incidence is allowed to be non-zero in each study population, either 1970, 1975, or 1980 depending on study site (see Section S1.4). Results for HIV incidence and new infections were summarised for ages 15 to 54 and years 2000 to 2017 or 2012 for Karonga and Manicaland based on most recently available data.

HIV incidence hazard $\lambda_{\theta_\lambda}(t,a)$

HIV incidence rate $\lambda_{\theta_\lambda}(t,a)$ was composed as a piecewise function

$$\lambda_{\theta_\lambda}(t,a) = \begin{cases} \lambda_{10}(t) & a = 10 \\ 0 & a \in (10,15). \\ \tilde{\lambda}(t,a) & a \geq 15 \end{cases}$$

The component of interest is the function $\tilde{\lambda}(t,a)$ defining age/time-specific HIV incidence rate. This was represented by a generalized additive model

$$\log \tilde{\lambda}(t,a) = f_t(t) + f_a(a) + f_{ta}(t,a).$$

The terms $f_t(t)$ and $f_a(a)$ specify average time trend and age pattern for incidence, respectively, and the interaction $f_{ta}(t,a)$ allows for a change in the incidence age pattern over time. The functions $f_t(t)$ and $f_a(a)$ are represented as penalised cubic B-splines ('p-splines')[1] with evenly spaced knots every five years and corresponding coefficients β_i^t, $i = \{1, \dots, I\}$ and β_j^a $j = \{1, \dots, J\}$. Letting $B_3(x)$ define the appropriate cubic B-spline basis functions, $f_t(t)$ and $f_a(a)$ are expressed as

$$f_t(t) = \sum_{i=1}^{I} \beta_i^t B_3(t)$$

$$f_a(t) = \sum_{j=1}^{J} \beta_j^a B_3(a).$$

The first order differences in β_i^t and β_j^a were penalised

$$\beta_i^t - \beta_{i-1}^t \sim N(0, \sigma_{\beta^t})$$

$$\beta_j^a - \beta_{j-1}^a \sim N(0, \sigma_{\beta^a}),$$

with prior distributions

$$\sigma_{\beta^t} \sim \text{half-Cauchy}(0, 2.5)$$

$$\sigma_{\beta^a} \sim \text{half-Cauchy}(0, 2.5).$$

The interaction term $f_{ta}(t, a)$ was modelled as a bivariate B-spline surface with a evenly spaced knots on a 5 year by 5 year lattice with coefficients β_{ij}^{at}, $i = 1, \ldots, I$, $j = 1, \ldots, J$,

$$f_{ta}(t, a) = \sum_{i=1}^{I} \sum_{j=1}^{J} \beta_{ij}^{at} B_3(t) B_3(a).$$

Differences between neighbouring spline coefficients are penalised using the conditional densities

$$\beta_{ij} | \boldsymbol{\beta}_{-ij} \sim N \left(\frac{\beta_{i-1,j} + \beta_{i,j-1} + \beta_{i+1,j} + \beta_{i,j+1}}{4}, \frac{\sigma_{\beta^{at}}^2}{4} \right),$$

and adjusted appropriately at the boundaries, resulting in the improper multivariate normal prior

$$p(\vec{\boldsymbol{\beta}}) \propto \sigma_{\beta ta}^{(I-1)\cdot(J-1)} \, exp \left\{ \frac{-1}{2\sigma_{\beta ta}^2} \vec{\boldsymbol{\beta}}' \boldsymbol{P} \vec{\boldsymbol{\beta}} \right\}$$

for singular precision matrix $\boldsymbol{P}$ of dimension $I \cdot J \times I \cdot J$ with rank $(I - 1) \cdot (J - 1)$.

The parameter $\sigma_{\beta ta}^{(I-1)\cdot(J-1)}$ had a prior distribution

$$\sigma_{\beta^{ta}} \sim \text{half-Cauchy}(0, 2.5).$$

In sensitivity analysis, we considered alternative specifications for the HIV incidence rate with only the additive components $\log \tilde{\lambda}(t, a) = f(t) + f(a)$, implying no change in the relative age pattern of HIV incidence over time, and only the interaction component $\log \tilde{\lambda}(t, a) = f(t, a)$, implying isotropic smoothing over age and time. See Supplementary Results Section S4.2 for results.

The term $\lambda_{10}(t)$ in the piecewise expression for $\lambda(t, a)$ allows for a proportion of those entering the model at age 10 to be already HIV positive resulting from long-term survival of mother-to-child transmission (MTCT) or infected sexually before age 15. We did not explicitly model the convolution of several processes determining the proportion entering as HIV positive at age 15— HIV prevalence among pregnant women, rates of mother-to-child

transmission, survival following MTCT, effects of PMTCT, effects of paediatric ART on child survival with HIV, and early sexual transmission. These processes are not independently identifiable from the population cohort data. Instead, the function $\lambda_{10}(t)$ empirically captures the time-varying survivorship outcome of these several processes, estimable by the excess proportion who are HIV positive upon cohort enrolment at age 15 above what would be expected based on cumulative incidence among this age group.

The function $\lambda_{10}(t)$ is defined as penalised cubic B-spline analogous to $f_t(t)$ with knots every five years and corresponding coefficients $\beta_i^{\lambda_{10}}, i = \{1, \dots, I\}$

$$\log \lambda_{10}(t) = \sum_{i=1}^{I} \beta_i^{\lambda_{10}} B_3(t)$$

First order differences in the spline coefficients were penalised by

$$\beta_i^{\lambda_{10}} - \beta_{i-1}^{\lambda_{10}} \sim N\left(0, \sigma_{\beta^t}\right),$$

noting that the penalty variance σ_{β^t} is shared with the penalty variance for the incidence time trend $f_t(t)$ defined above.

Non-HIV mortality hazard $\mu_{\theta_\mu}(t, a)$

The non-HIV mortality was modelled as a generalized additive model

$$\log \mu_{\theta_\mu}(t, a) = g_t(t) + g_a(a)$$

where each of the functions $g_t(t)$ and $g_a(a)$ were represented by penalised cubic l B-splines with evenly spaced knots every 5-years, with coefficients $\gamma_i^t$and γ_j^a. The function $g(t)$ was defined such that non-HIV mortality is assumed to be constant before the start of demographic surveillance in each cohort (see Table S2), that is if demographic surveillance began at time t_{start}, then

$$g_t(t) = \begin{cases} g_t(t_{start}) & \text{if } t < t_{start} \\ g_t(t) & \text{if } t \geq t_{start} \end{cases}.$$

Thus $g_t(t)$ is only defined on a subset of knots γ_i^t, $i \in \{I_{start}, \dots, I\}$. For identifiability, the sum-to-zero constraint was applied $\sum_1^J \gamma_j^a = 0$. First order knot differences were penalised

$$\gamma_i^t - \gamma_{i-1}^t \sim N(0, \sigma_{\gamma^t})$$

$$\gamma_j^a - \gamma_{j-1}^a \sim N(0, \sigma_{\gamma^a})$$

with prior distributions

$$\sigma_{\gamma^t} \sim \text{half-Cauchy}(0, 2.5)$$

$$\sigma_{\gamma^a} \sim \text{half-Cauchy}(0, 2.5).$$

Excess HIV mortality hazard $\rho_{\theta_\rho}(t, a, u)$

The excess HIV mortality hazard $\rho_{\theta_\rho}(t, a, u)$ is composed of a Weibull distribution $\omega(a_0, u)$ describing the natural survival distribution as a function of age at HIV infection a_0 and duration of infection u and a function $h_{ART}(t)$ describing the relative reduction in the HIV mortality rate over time after ART is available:

$$\rho_{\theta_\rho}(t, a, u) = \omega(a - u, u) \cdot h_{ART}(t).$$

The Weibull hazard function is parameterised by the shape parameter α and scale parameter η_{a_0}, depending on age of infection a_0

$$\omega(a_0, u) = \frac{\alpha^\omega}{\eta_{a_0}} \cdot \left(\frac{u}{\eta_{a_0}}\right)^{\alpha^\omega - 1}$$

$$\log \eta_{a_0} = \beta_0^\omega + \beta_1^\omega \cdot (a_0 - 30)/10$$

$$\beta_0^\omega \sim N(2.55, \text{sd} = 0.25)$$

$$\beta_1^\omega \sim N(-0.2, \text{sd} = 0.05)$$

$$\alpha^\omega \sim \text{Gamma}(\text{shape} = 12, \text{rate} = 6)$$

Informative prior distributions for the time from infection to death are derived based on Weibull regression results reported by Todd et al[2] and uses parameterisation used by Bellan

et al.[3] The Gamma(shape $= 12$, rate $= 6$) prior distribution for the shape parameter α^ω has a mean of 2.0 with 95% of the mass between 1.0 and 3.3. The prior mean for β_1^ω implies an 18% reduction in HIV survival per ten-year increase in age at seroconversion. The joint prior distribution for $\{\alpha^\omega, \beta_0^\omega, \beta_1^\omega\}$ implies median HIV survival of 14.7 years with 95% of prior range from 8.3 to 23.9 years for someone infected at age 15, 12.0 years (95% range 6.9–19.2) for someone infected at age 25, 9.8 years (5.7–15.8) when infected at age 35, and 8.1 years (4.6–13.2) when infected at age 45 (Figure S2).

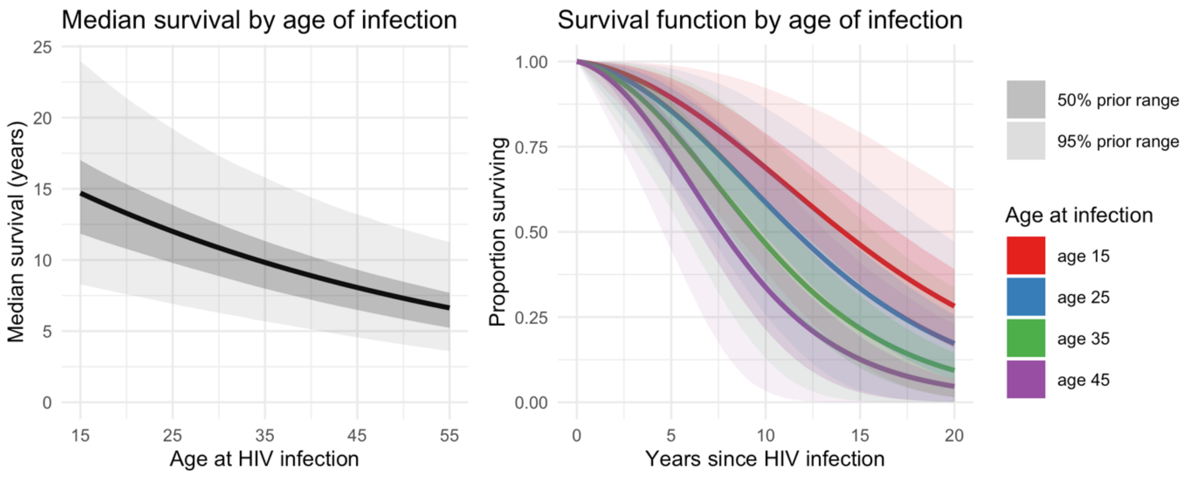

Figure S2: Joint prior distribution for HIV survival by age of infection. (Left) Median survival by age of infection. Solid line represents the prior mean and shaded areas reflect 50% and 95% of prior mass. (Right) Proportion surviving by duration of HIV infection for persons infected at age 15, 25, 35, or 45.

The effect of ART on excess HIV mortality is modelled by $h_{ART}(t)$, representing the relative reduction in HIV mortality at time t after ART becomes available in the population, that is

$$h_{ART}(t) = \begin{cases} 1 & \text{if } t < t_{\text{ART}} \\ h^+_{\text{ART}}(t) & \text{if } t \geq t_{\text{ART}} \end{cases}.$$

The function $h^+_{ART}(t)$ is defined to satisfy three properties: (1) continuity of $h(t)$, that is $h_{ART}(t_{ART}) = 1$, (2) monotonically decreasing over time, such that the effect of ART on reducing mortality increases, and (3) positivity, to ensure $\rho(t, a, u) > 0$. This is achieved by defining $\log h_{ART}(t)$ as an integral function

$$\log h^{+}_{ART}(t) = \int_{t_{ART}}^{t} dh(t)\ dt$$

where $dh(t)$ is a discrete first-order random walk with strictly negative terms, that is

$$dh(t) = dh_x, t \in [x, x + 0.2)$$

where the constants $dh_x \leq 0$ and first-order differences are penalised

$$dh_x - dh_{x-0.2} \sim N(0, \sigma_{ART}).$$

The prior distribution for σ_{ART} is

$$\sigma_{ART} \sim \text{half-Cauchy}(0, 2.5).$$

S1.3 Model implementation

For computation, the model was discretised to $dt = 0.2$ year steps in time and age. The observation dates $t_i^B, t_i^F, t_i^E, t_i^N, t_i^P$ for each individual were rounded to the nearest 0.2 year time step and each of the hazard functions $\lambda(t, a)$, $\mu(t, a)$, and $\rho(t, a, u)$ were converted piecewise-constant functions, enabling the integrals defined in section S1.1 to be calculated as discrete sums. This discretisation enabled computational tractability of the integrals and facilitated binning of individuals into discrete birth cohorts, enabling cumulative hazards and survivorship functions to be computed once for each cohort and re-used in individual likelihood computation for all individuals in that birth cohort. We also tested a finer discretisation interval of $dt = 0.1$ years and results were negligibly different.

The model was estimated independently for each study site. Within each study site, the coefficients determining the hazard functions ($\beta_i^t, \beta_j^a, \beta_{ij}^{ta}, \beta_i^{\lambda_{10}}, \gamma_i^t, \gamma_j^a, \alpha^\omega, \beta_0^\omega, \beta_1^\omega$, and dh_x) were estimated separately for each sex, but the smoothing hyper-parameters $\sigma_{\beta^t}, \sigma_{\beta^a}, \sigma_{\beta^{at}}, \sigma_{\gamma^t}, \sigma_{\gamma^a}$ and σ_{ART} were shared for both sexes in each study site.

Demographic surveillance and HIV sero-surveillance only began several years into the epidemic in each study site, when HIV prevalence was already relatively high. To incorporate expert knowledge that HIV prevalence and cumulative incidence were low during the late 1970s and early 1980s, we added auxiliary data implying low HIV prevalence five years after the model start t_0 (see Table S2). A single pseudo-observation of an HIV negative adult in the year $t_0 + 5$ was input at every 0.1 year interval between ages 15 and 60 (451 pseudo-individuals total).

S1.4 Population under demographic surveillance

Table S1a and b show a description of the age and sex distribution of the adult population under demographic surveillance at four timepoints during the study period in each of the ALPHA studies.

Table S1a: Distribution of adult population under demographic surveillance by age and sex at four timepoints (2002, 2007, 2012, and 2017) for each cohort study, showing n and (%).

		Female				Male			
	**Age**	**2002**	**2007**	**2012**	**2017**	**2002**	**2007**	**2012**	**2017**
**Karonga**	15-19	1118 (18.8)	1659 (18.3)	1916 (19.0)		1133 (21.4)	1571 (19.7)	1844 (21.4)	
	20-24	1036 (17.4)	1497 (16.5)	1479 (14.6)		974 (18.4)	1445 (18.1)	1257 (14.6)	
	25-29	817 (13.7)	1303 (14.4)	1377 (13.6)		774 (14.6)	1156 (14.5)	1168 (13.6)	
	30-34	610 (10.2)	1040 (11.5)	1178 (11.7)		537 (10.1)	965 (12.1)	985 (11.4)	
	35-39	488 (8.2)	679 (7.5)	967 (9.6)		453 (8.5)	644 (8.1)	868 (10.1)	
	40-44	359 (6.0)	639 (7.1)	651 (6.4)		316 (6.0)	544 (6.8)	564 (6.6)	
	45-49	349 (5.9)	458 (5.1)	600 (5.9)		244 (4.6)	372 (4.7)	503 (5.8)	
	50-54	228 (3.8)	430 (4.8)	421 (4.2)		179 (3.4)	282 (3.5)	342 (4.0)	
	55+	947 (15.9)	1343 (14.8)	1508 (14.9)		692 (13.1)	999 (12.5)	1073 (12.5)	
**Kisesa**	15-19	892 (17.0)	1294 (18.0)	1688 (19.8)	1600 (19.2)	1089 (21.0)	1498 (22.1)	1783 (22.6)	1709 (22.2)
	20-24	678 (12.9)	1122 (15.6)	1177 (13.8)	1146 (13.7)	815 (15.7)	1002 (14.8)	1172 (14.9)	1213 (15.7)
	25-29	762 (14.5)	869 (12.1)	1082 (12.7)	991 (11.9)	656 (12.6)	802 (11.8)	826 (10.5)	784 (10.2)
	30-34	597 (11.4)	935 (13.0)	923 (10.8)	862 (10.3)	519 (10.0)	825 (12.2)	795 (10.1)	688 (8.9)
	35-39	558 (10.6)	610 (8.5)	950 (11.1)	824 (9.9)	507 (9.8)	592 (8.7)	862 (10.9)	796 (10.3)
	40-44	414 (7.9)	600 (8.3)	580 (6.8)	702 (8.4)	431 (8.3)	532 (7.9)	589 (7.5)	677 (8.8)
	45-49	348 (6.6)	453 (6.3)	562 (6.6)	557 (6.7)	305 (5.9)	441 (6.5)	521 (6.6)	483 (6.3)
	50-54	233 (4.4)	343 (4.8)	438 (5.1)	508 (6.1)	220 (4.2)	321 (4.7)	411 (5.2)	446 (5.8)
	55+	777 (14.8)	980 (13.6)	1122 (13.2)	1153 (13.8)	656 (12.6)	761 (11.2)	924 (11.7)	918 (11.9)
**Manicaland**	15-19	812 (17.4)	1166 (14.7)	782 (14.4)		419 (12.6)	999 (19.6)	785 (22.0)	
	20-24	866 (18.6)	1530 (19.3)	743 (13.7)		946 (28.4)	1268 (24.9)	659 (18.5)	
	25-29	730 (15.7)	1047 (13.2)	784 (14.4)		665 (20.0)	813 (15.9)	495 (13.9)	
	30-34	570 (12.2)	1000 (12.6)	667 (12.3)		355 (10.7)	614 (12.0)	418 (11.7)	
	35-39	555 (11.9)	803 (10.1)	633 (11.6)		303 (9.1)	404 (7.9)	403 (11.3)	
	40-44	538 (11.6)	762 (9.6)	500 (9.2)		227 (6.8)	340 (6.7)	260 (7.3)	
	45-49	405 (8.7)	752 (9.5)	462 (8.5)		175 (5.3)	267 (5.2)	217 (6.1)	
	50-54	124 (2.7)	633 (8.0)	476 (8.7)		123 (3.7)	228 (4.5)	174 (4.9)	
	55+	58 (1.2)	243 (3.1)	394 (7.2)		119 (3.6)	168 (3.3)	154 (4.3)	

Table S1b: Distribution of adult population under demographic surveillance by age and sex at four timepoints (2002, 2007, 2012, and 2017) for each cohort study, showing n and (%).

		Female				Male			
	**Age**	**2002**	**2007**	**2012**	**2017**	**2002**	**2007**	**2012**	**2017**
**Masaka**	15-19	933 (22.9)	844 (20.6)	1022 (22.1)	989 (22.3)	973 (25.3)	959 (25.9)	1058 (26.1)	1006 (26.5)
	20-24	570 (14.0)	485 (11.8)	540 (11.7)	549 (12.4)	538 (14.0)	443 (12.0)	540 (13.3)	488 (12.9)
	25-29	481 (11.8)	451 (11.0)	447 (9.7)	403 (9.1)	420 (10.9)	334 (9.0)	374 (9.2)	339 (9.0)
	30-34	375 (9.2)	441 (10.8)	447 (9.7)	390 (8.8)	373 (9.7)	346 (9.4)	351 (8.7)	307 (8.1)
	35-39	358 (8.8)	322 (7.9)	424 (9.2)	387 (8.7)	326 (8.5)	320 (8.6)	344 (8.5)	308 (8.1)
	40-44	261 (6.4)	346 (8.4)	308 (6.7)	360 (8.1)	236 (6.1)	290 (7.8)	321 (7.9)	291 (7.7)
	45-49	191 (4.7)	259 (6.3)	345 (7.5)	273 (6.1)	211 (5.5)	200 (5.4)	257 (6.3)	281 (7.4)
	50-54	205 (5.0)	186 (4.5)	265 (5.7)	287 (6.5)	140 (3.6)	199 (5.4)	184 (4.5)	208 (5.5)
	55+	709 (17.4)	764 (18.6)	817 (17.7)	806 (18.1)	631 (16.4)	609 (16.5)	623 (15.4)	565 (14.9)
**Rakai**	15-19	1325 (21.1)	1651 (20.5)	1970 (20.9)	2870 (20.4)	1257 (23.7)	1490 (21.5)	1883 (22.3)	2686 (21.3)
	20-24	1115 (17.8)	1468 (18.3)	1429 (15.2)	2351 (16.7)	905 (17.0)	1122 (16.2)	1174 (13.9)	1915 (15.2)
	25-29	947 (15.1)	1313 (16.3)	1416 (15.0)	1926 (13.7)	861 (16.2)	1044 (15.1)	1150 (13.6)	1775 (14.0)
	30-34	596 (9.5)	953 (11.9)	1248 (13.3)	1755 (12.5)	690 (13.0)	1019 (14.7)	1088 (12.9)	1459 (11.5)
	35-39	488 (7.8)	579 (7.2)	906 (9.6)	1531 (10.9)	503 (9.5)	725 (10.5)	988 (11.7)	1465 (11.6)
	40-44	402 (6.4)	431 (5.4)	568 (6.0)	979 (7.0)	350 (6.6)	500 (7.2)	715 (8.5)	1124 (8.9)
	45-49	308 (4.9)	401 (5.0)	436 (4.6)	603 (4.3)	223 (4.2)	329 (4.8)	496 (5.9)	809 (6.4)
	50-54	260 (4.1)	302 (3.8)	387 (4.1)	534 (3.8)	122 (2.3)	202 (2.9)	284 (3.4)	468 (3.7)
	55+	830 (13.2)	944 (11.7)	1050 (11.2)	1516 (10.8)	402 (7.6)	495 (7.1)	675 (8.0)	937 (7.4)
**uMkhanyakude**	15-19	4785 (21.2)	4539 (19.9)	4479 (18.5)	3492 (15.8)	4788 (29.8)	4640 (29.0)	4632 (26.5)	3702 (23.3)
	20-24	3120 (13.8)	3516 (15.4)	3483 (14.4)	2756 (12.5)	2612 (16.2)	2932 (18.3)	3220 (18.4)	2637 (16.6)
	25-29	2641 (11.7)	2301 (10.1)	2979 (12.3)	2583 (11.7)	1827 (11.4)	1672 (10.5)	2325 (13.3)	2042 (12.8)
	30-34	2079 (9.2)	2104 (9.2)	2095 (8.6)	2337 (10.6)	1378 (8.6)	1383 (8.7)	1545 (8.8)	1801 (11.3)
	35-39	1926 (8.5)	1744 (7.6)	1932 (8.0)	1760 (8.0)	1212 (7.5)	1073 (6.7)	1273 (7.3)	1204 (7.6)
	40-44	1802 (8.0)	1684 (7.4)	1600 (6.6)	1575 (7.1)	1013 (6.3)	975 (6.1)	934 (5.3)	1051 (6.6)
	45-49	1249 (5.5)	1619 (7.1)	1626 (6.7)	1364 (6.2)	822 (5.1)	799 (5.0)	887 (5.1)	740 (4.7)
	50-54	1071 (4.7)	1176 (5.2)	1568 (6.5)	1422 (6.4)	623 (3.9)	688 (4.3)	715 (4.1)	725 (4.6)
	55+	3930 (17.4)	4144 (18.2)	4466 (18.4)	4826 (21.8)	1803 (11.2)	1823 (11.4)	1978 (11.3)	2004 (12.6)

Demographic surveillance dates for the ALPHA dataset analysed in this manuscript for each study are: Karonga from 15 June 2002 to 1 January 2018; Kisesa from 7 January 1994 to 27 July 2017; Manicaland from 15 March 1998 to 14 December 2013; Masaka from 12 November 1989 to 25 April 2017; Rakai from 1 November 1994 to 3 August 2016; and uMkhanyakude from 1 January 2000 to 31 December 2017. Note: demographic surveillance in Karonga and Manicaland extends beyond the end of their most recent serosurveillance, which ended in 2012.

S1.5 HIV serosurvey data by study

Table S2 describes the assumed start year t_0 for HIV incidence in each study population, the year in which demographic surveillance was established (t_{start}), the year of the first HIV serosurvey, the year of the most recent HIV serosurvey, the number of population-wide serosurveys conducted, and the year in which ART first became available to the study population. The year $t_0 + 5$ in which the zero-prevalence prior was applied was 1975 for Masaka, Rakai, and Kisesa (all around Lake Victoria), in 1980 for Karonga and Manicaland (further south, and based on early prevalence surveys in Karonga), and in 1985 for uMkhanyakude in South Africa.

Table S2: Date of epidemic start, first demographic surveillance and HIV serosurveys for each cohort study.

Study name (Country)	Epidemic start (t_0)[a]	Demographic surveillance start (t_{start})[b]	First HIV survey	Most recent HIV survey	Number of HIV surveys	ART start (t_{ART})
Karonga (Malawi)	1975	2002	2007[c]	2011	4[c]	2005
Kisesa (Tanzania)	1970	1994	1994	2016	8	2005
Manicaland (Zimbabwe)	1975	1998	2002	2012	6	2005
Masaka (Uganda)	1970	1989	1989	2018	25	2004
Rakai (Uganda)	1970	1994	1995	2016	17	2004
uMkhanyakude (S Africa)	1980	2000	2003	2017	14	2005

[a] first year of model estimation, with zero-prevalence pseudo data incorporated at year $t_0 + 5$; [b] start year of demographic surveillance; [c] for Karonga study, HIV serosurvey data were incorporated from four population serosurveys conducted prior to establishment of the current demographic surveillance population.

HIV serosurvey data were used for resident adults (age 15+ years). Table S3 summarises the number of tests conducted and the age range of the study population in each decade, and Table S4 summarises the same among HIV-positive tests. Of the six studies included, only Masaka includes a paediatric population for HIV testing, which are not included in these analyses. Other studies primarily include individuals of reproductive age in testing, though some studies include older individuals (Table S3). We used only HIV tests that were administered in the context of a survey testing round in this analysis, excluding any HIV test results reported based on links to clinical records, non-representative special studies, or self-reported HIV status. Any individuals who were HIV-positive in one visit and have a follow-up test that is HIV-negative were excluded from analyses (n= 114).

Table S3: Number and age of participants in HIV serosurveys in each study cohort by decade, ALPHA Network.

	Pre 2000		2000-2009		2010-	
Study cohort	med (IQR) [95%][1]	N	med (IQR) [95%][1]	N	med (IQR) [95%][1]	N
Karonga	40 (25-54) [16-70]	299	29 (21-42) [15-73]	25585	30 (21-42) [15-72]	20741
Karonga - pre data	31 (21-44) [15-69]	24303	35 (27-46) [17-72]	779		
Kisesa	26 (20-34) [15-52]	14558	30 (21-43) [15-73]	21297	31 (21-46) [15-76]	21716
Manicaland	26 (20-35) [16-52]	8860	27 (20-38) [15-54]	38976	30 (21-41) [15-58]	21243
Masaka	31 (20-48) [15-76]	32600	31 (20-47) [15-76]	55049	34 (21-49) [15-77]	25241
Rakai	27 (21-37) [15-56]	28844	27 (21-35) [15-48]	51333	28 (21-36) [15-47]	39718
uMkhanyakude			26 (19-43) [15-75]	60230	33 (20-54) [15-81]	87226

[1] Table denotes the median age of HIV testing participants, interquartile range (IQR) of age at test, 2.5th and 97.5th percentiles [95%] of age at test, and number of tests conducted in the study population (N).

Table S4 Number and age of HIV-positive participants in HIV serosurveys in each study cohort by decade, ALPHA Network.

	Pre 2000		2000-2009		2010-	
Study cohort	med (IQR) [95%][1]	N	med (IQR) [95%][1]	N	med (IQR) [95%][1]	N
Karonga	33 (27-36) [23-40]	7	36 (30-45) [20-61]	1771	37 (32-45) [20-63]	1207
Karonga – pre data	33 (25-41) [17-60]	354	35 (29-40) [19-58]	104		
Kisesa	29 (25-35) [18-47]	901	32 (26-40) [17-62]	1267	37 (30-46) [19-66]	1448
Manicaland	30 (25-37) [18-50]	2015	33 (27-40) [19-53]	6965	37 (31-45) [18-57]	3445
Masaka	30 (25-39) [18-64]	2701	33 (27-41) [18-61]	3353	38 (30-47) [18-64]	2001
Rakai	29 (24-35) [17-52]	4678	31 (26-37) [19-47]	6402	34 (28-40) [19-48]	5252
uMkhanyakude			31 (24-40) [17-58]	12388	35 (27-46) [18-66]	23879

[1] Table denotes the median age of HIV-positive testing participants, interquartile range (IQR) of age at test, 2.5th and 97.5th percentiles [95%] of age at test, and number of positive tests conducted in the study population (N).

Additional HIV tests from the Karonga study area prior to the establishment of the health and demographic surveillance system (HDSS), consisting of HIV tests conducted retrospectively on samples collected between 1982-2004 were included (“Karonga pre-data” in Table S3 and S4).[4,5]

In years beyond the HDSS collection dates (start dates presented in Table 1 of main manuscript) or more recent years with only partial data collection, we assume exponential growth of the population for the study population to which results are standardised. This assumption applies to: 2000-2003 in Karonga, 2017 in Masaka, 2016-17 in Rakai, and 2017 in Kisesa. uMkhanyakude dramatically increased the population under surveillance in 2017, so for continuity we only include individuals that are under surveillance prior to this shift. In-migrants to the continuously surveilled area will be excluded in 2017, though we anticipate this will have minimal impact on the results presented.

S1.6 Data sharing

Contact information for the study sites are as follows: Karonga - Amelia (Mia) Crampin (http://meiru.lshtm.ac.uk/); Kisesa – Mark Urassa (via ALPHA https://alpha.lshtm.ac.uk/); Manicaland – Simon Gregson (http://www.manicalandhivproject.org/data-access.html); Masaka (via ALPHA https://alpha.lshtm.ac.uk/); Rakai – Tom Lutalo (tlutalo@rhsp.org); and uMkhanyakude – Africa Health Research Institute data repository (https://data.ahri.org).

S2 Supplemental Model Outputs

S2.1 Data to reproduce main figures

Spreadsheets containing the data to recreate all figures in the main manuscript can be found at the following link (https://github.com/krisher1/ALPHA-age-patterns-of-HIV-incidence/blob/main/data_for_figures_alpha_age_patterns.xlsx?raw=true).

S2.2 HIV trends among age 15-54

Figure S3, Figure S4, and Figure S5 show the HIV prevalence, HIV incidence rate, and cumulative HIV incidence probability, respectively, for ages 15-54 years for each sex and study based on the joint Bayesian model for HIV incidence and mortality presented in the main manuscript. HIV prevalence ranged from less than 15% in Kisesa, Masaka and Karonga, to 10-25% in Rakai and Manicaland, to 20-45% in uMkhanayakude. From 2000 to 2017, prevalence remained relatively stable in Kisesa, Masaka and Rakai, decreased in Manicaland and Karonga, and increased in uMkhanyakude. Within each study, trends were similar for both sexes and men had consistently lower estimated prevalence than women. Trends in HIV incidence rate were more variable, but decreased in the final years of the study period in all six studies. Period cumulative incidence (probability of infection) between ages 15-54 was lowest in Kisesa and Masaka, followed by Rakai, Karonga, Manicaland and

uMkhanyakude. In uMkhanyakude in the early 2000s, cumulative incidence was about 80% over ages 15-55 in both sexes.

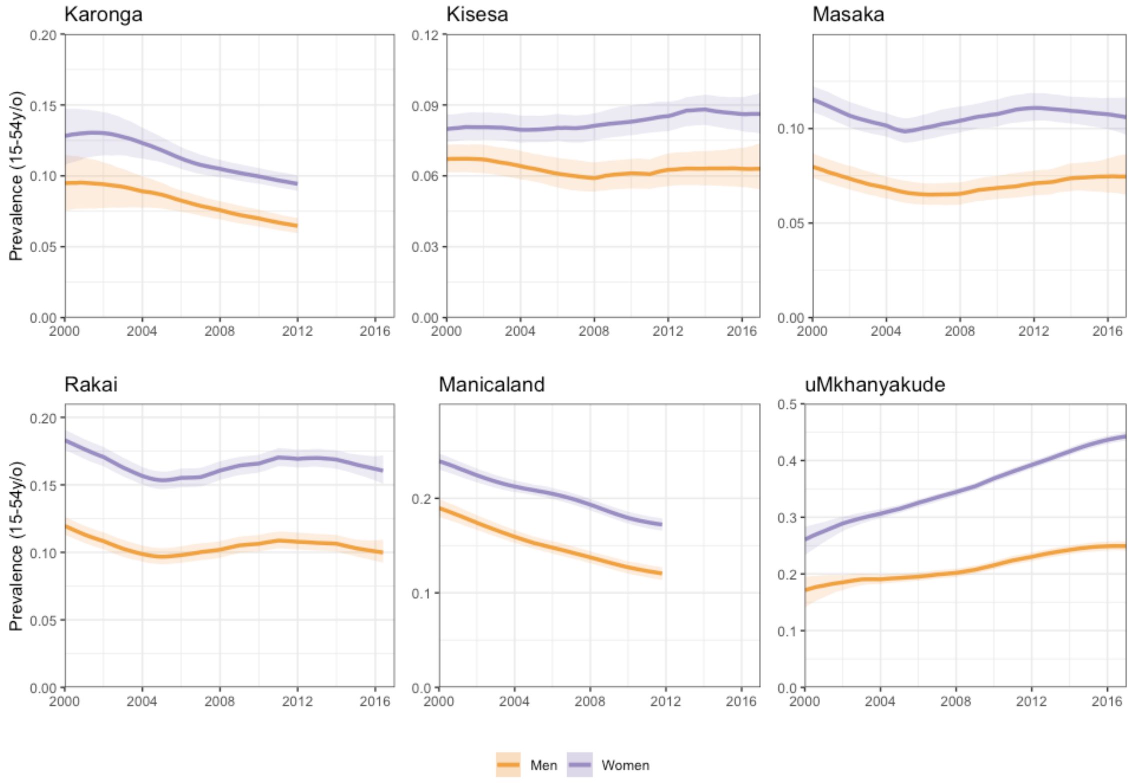

Figure S3: Estimated HIV prevalence for 15-54 year olds from 2000-2017 in six studies.

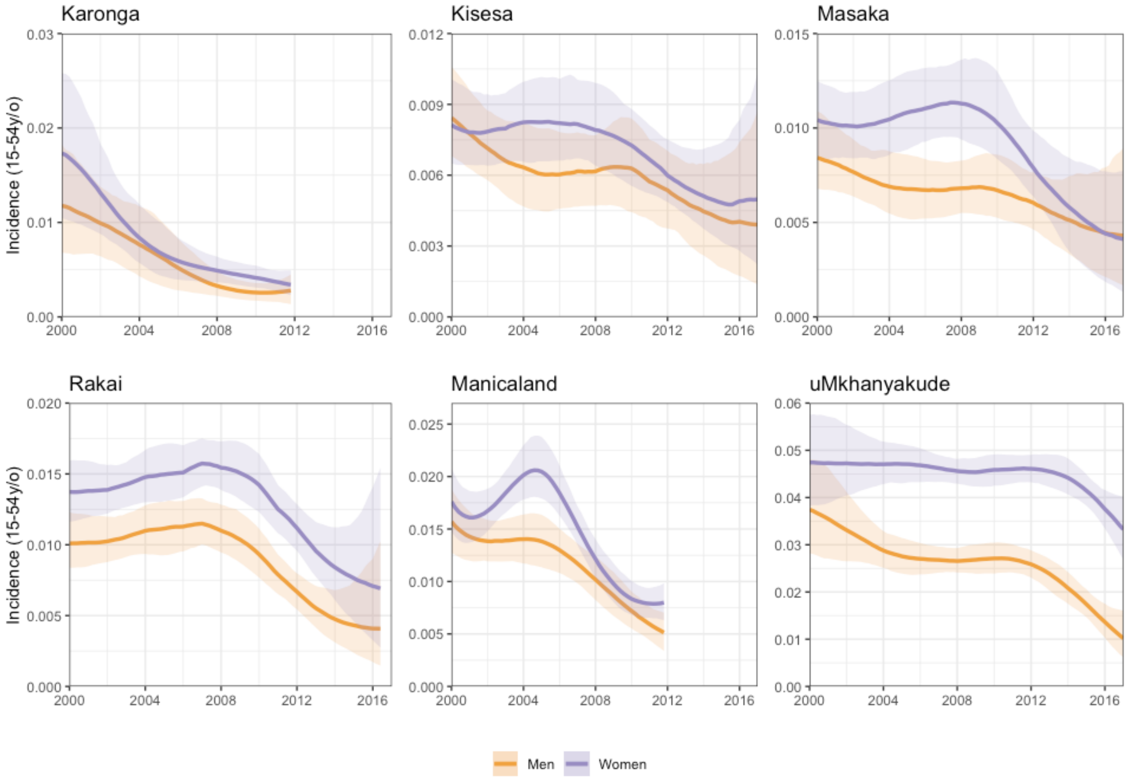

Figure S4: Estimated HIV incidence rate for 15-54 year olds from 2000-2017 in six studies.

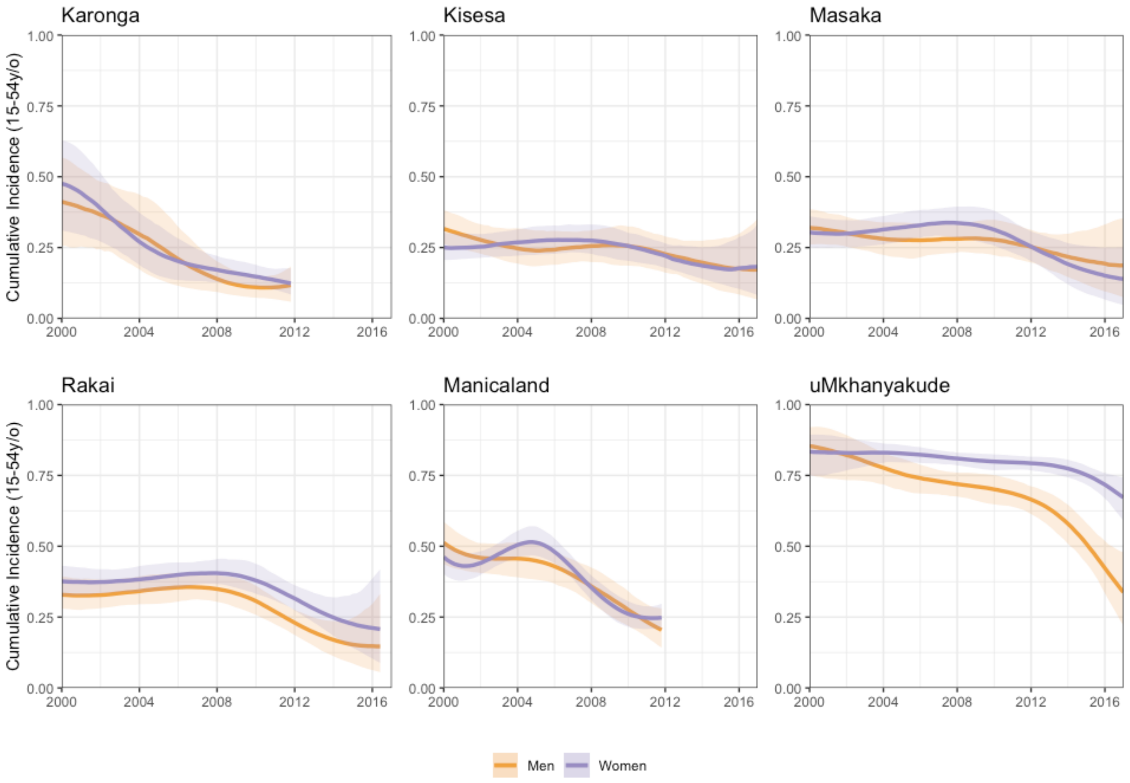

Figure S5: Estimated cumulative HIV incidence between ages 15-54 years from 2000-2017 in six studies.

S2.3 Estimate for proportion HIV positive upon entry to population.

Figure S6 shows the estimate for the function $\lambda_{10}(t)$ determining the proportion who are HIV positive upon entry to the model population at age 10. This trend captures long-term survivors of mother-to-child transmission and early sexual transmission, but is not directly interpretable as incidence rate for newborns or early sexual transmission as both MTCT rate and child survival with HIV vary over time.

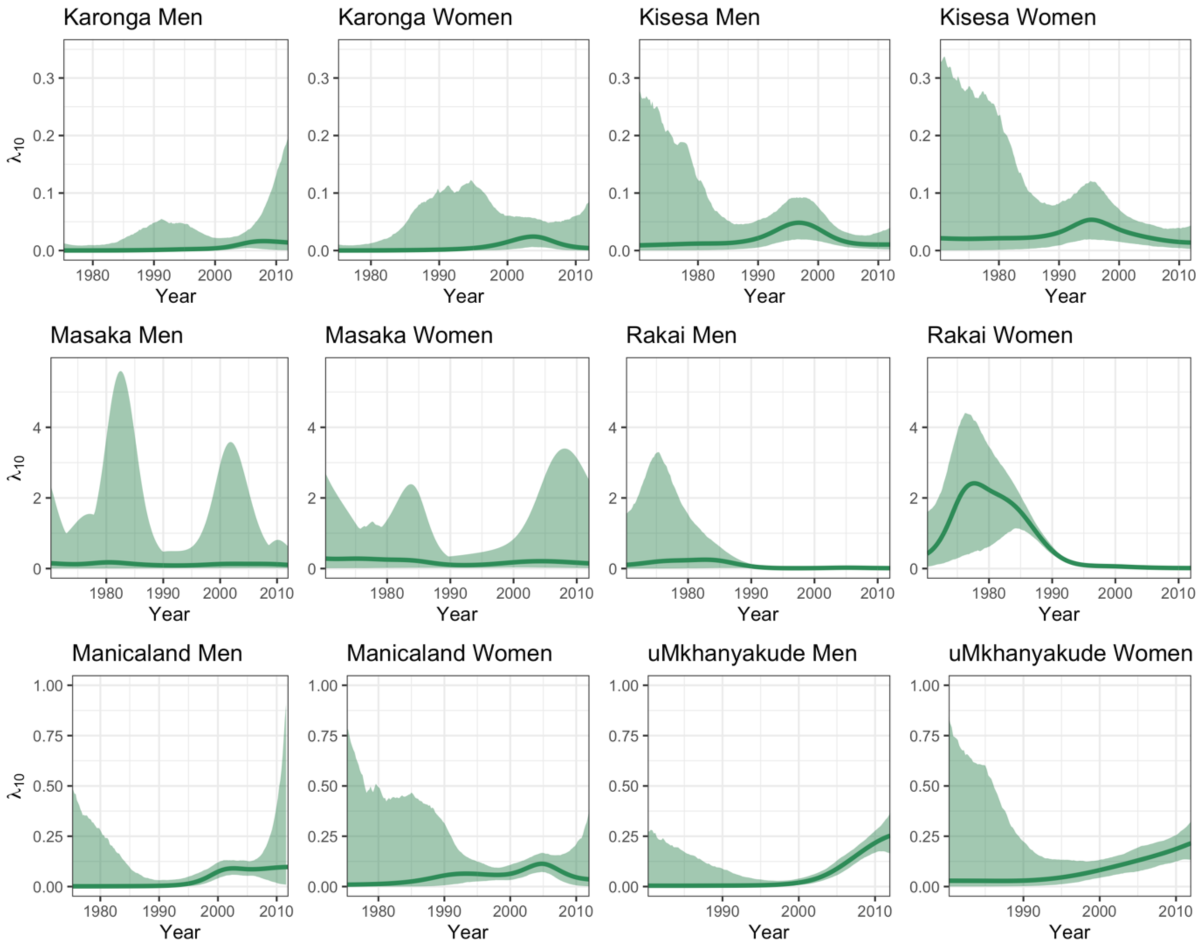

Figure S6: Estimate for $\lambda_{10}(t)$, an annual rate, determining the proportion HIV positive at age 10 upon entry to the model population.

S2.4 Age distribution of HIV infections

Figure S7 shows the estimated number of new HIV infections by five-year age group. The number of infections has decreased in the most recent years in all studies (with the possible exception of final years in which incidence rates are poorly informed by data resulting in large uncertainty). Presented jagged patterns across years in Kisesa and Rakai are reflective of variations in the size of the population in the study areas between demographic surveillance rounds, not changes in incidence patterns.

Figure S8 shows the distribution of new infections by age group in the most recent year available. Figure S9 shows quantiles of the distribution of new infections over time. The median age at infection changed minimally over time in most studies, though the percentiles across ages changes somewhat in studies such as uMkhanyakude, where the middle 40th, 60th and 80th percentiles narrow in both men and women starting around 2008.

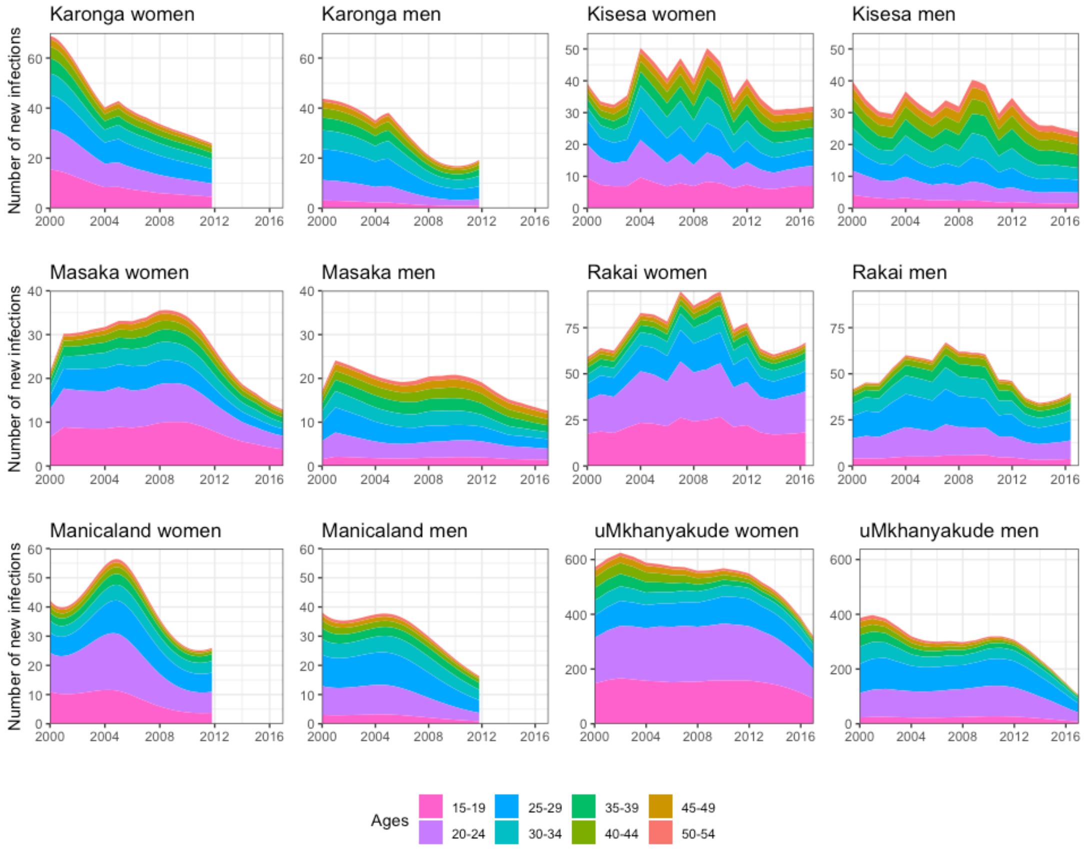

Figure S7: Number of new infections by five-year age group from 2000-2017 in six studies.

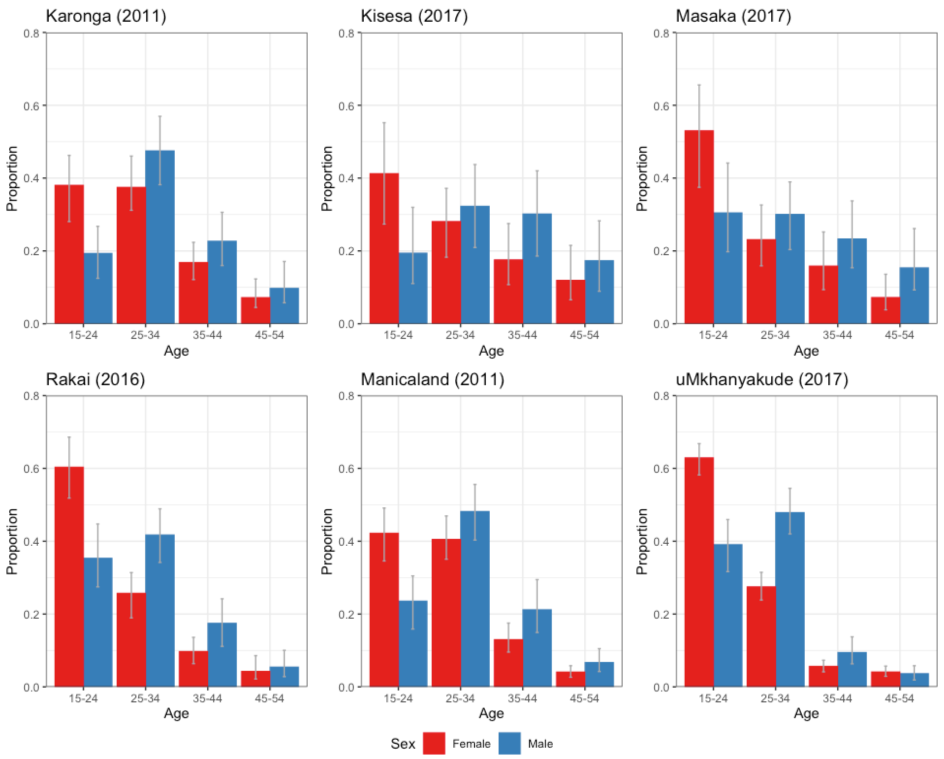

Figure S8: Proportion infected by ten-year age group for each sex at most recently observed time point in six studies.

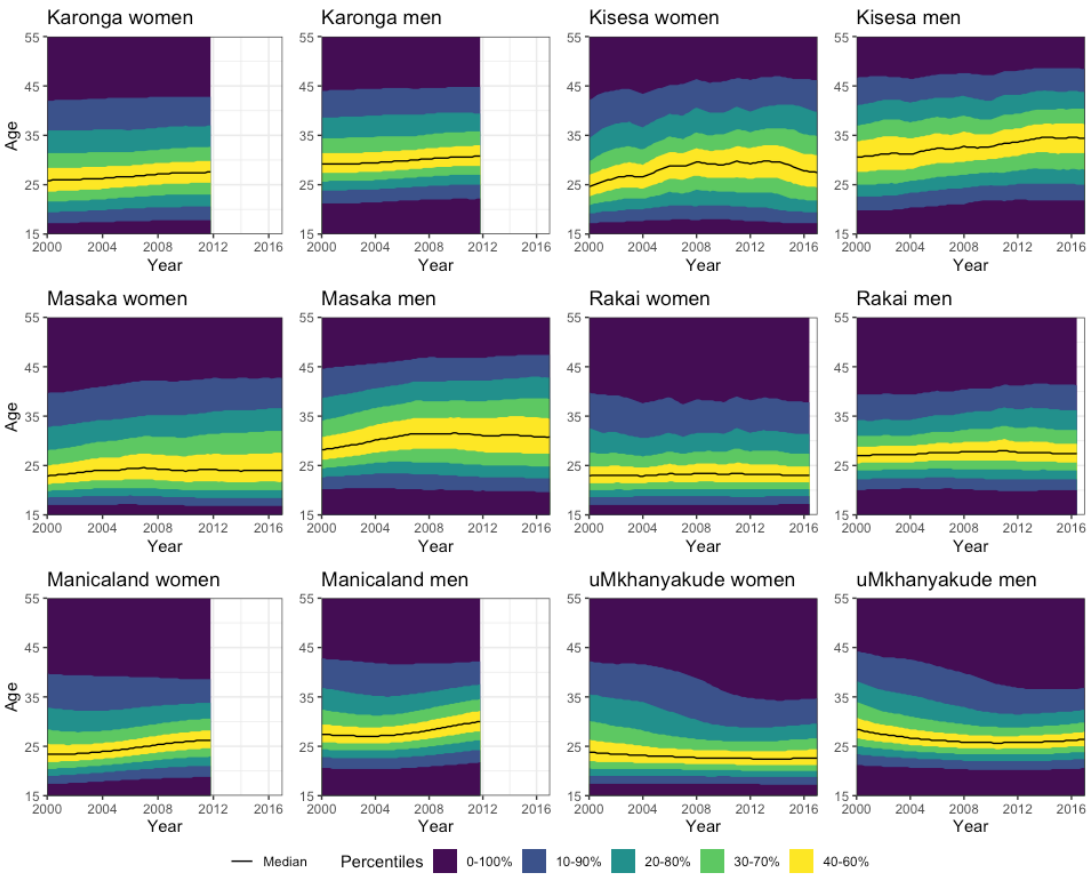

Figure S9: Median age at infection and percentile bands from 2000-2017 in six studies.

S2.5 HIV incidence trend by single-year age

Figure S10 through Figure S13 show the estimated HIV incidence trend by single-year of age from 15-54 in ten-year age bands (15-24, 25-34, 35-44, 45-54). Note that Rakai only collect HIV serosurvey data on individuals aged 15-49, so incidence in 50-54 age group is exclusively extrapolated from the trend at younger ages and mortality data.

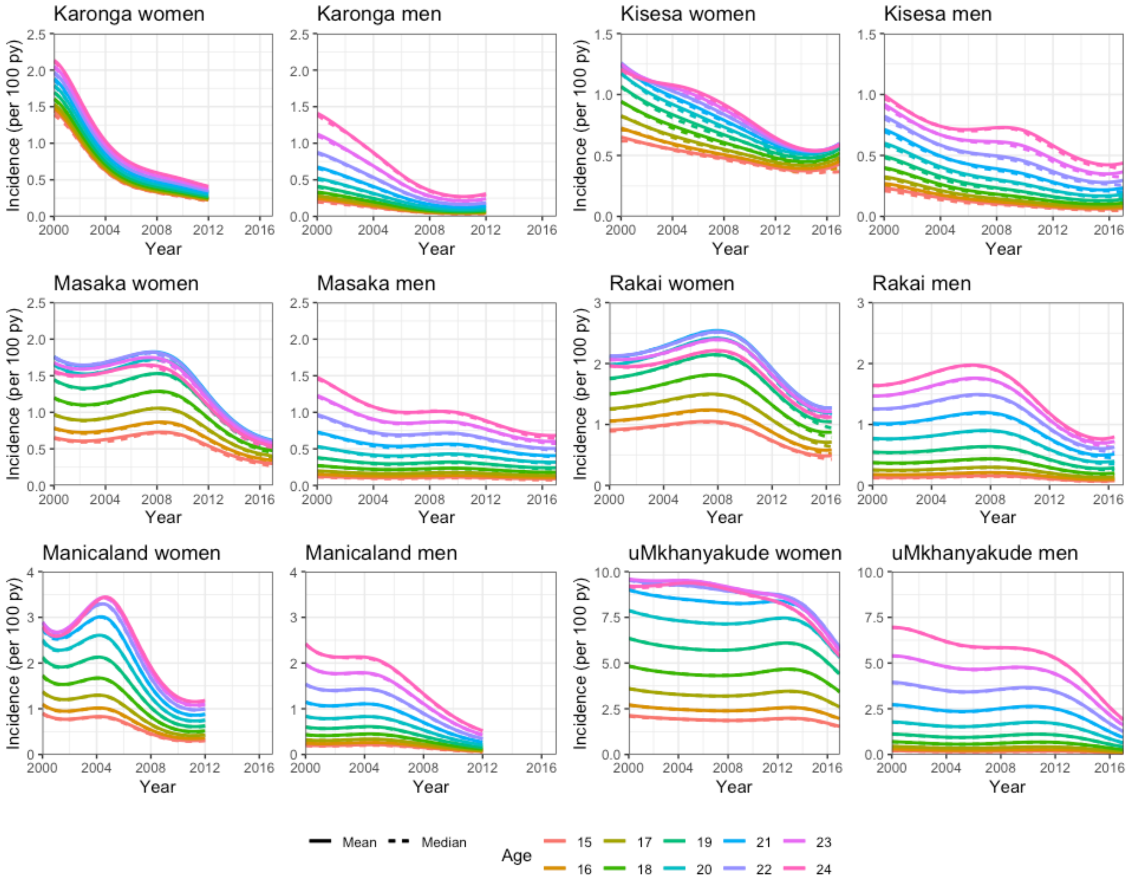

Figure S10: Estimated HIV incidence rate by single age 15-24.

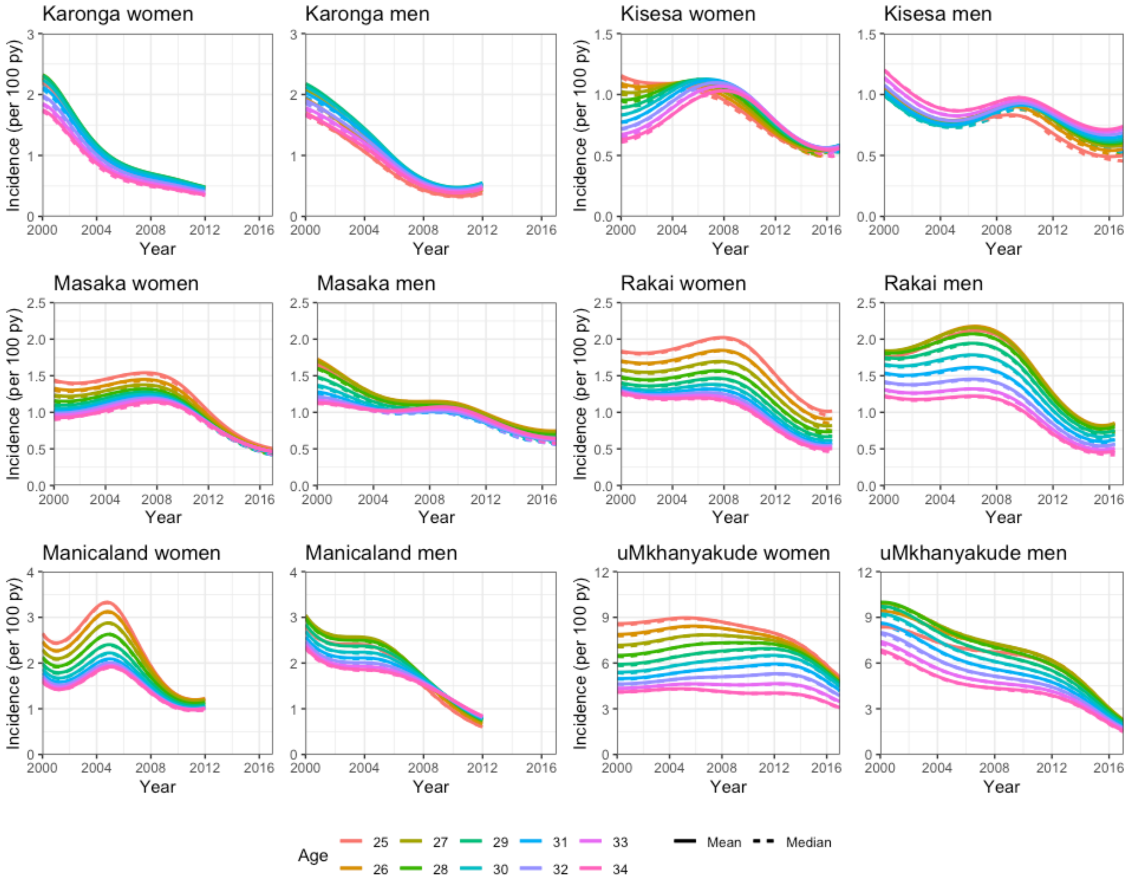

Figure S11: Estimated HIV incidence rate by single age 25-34.

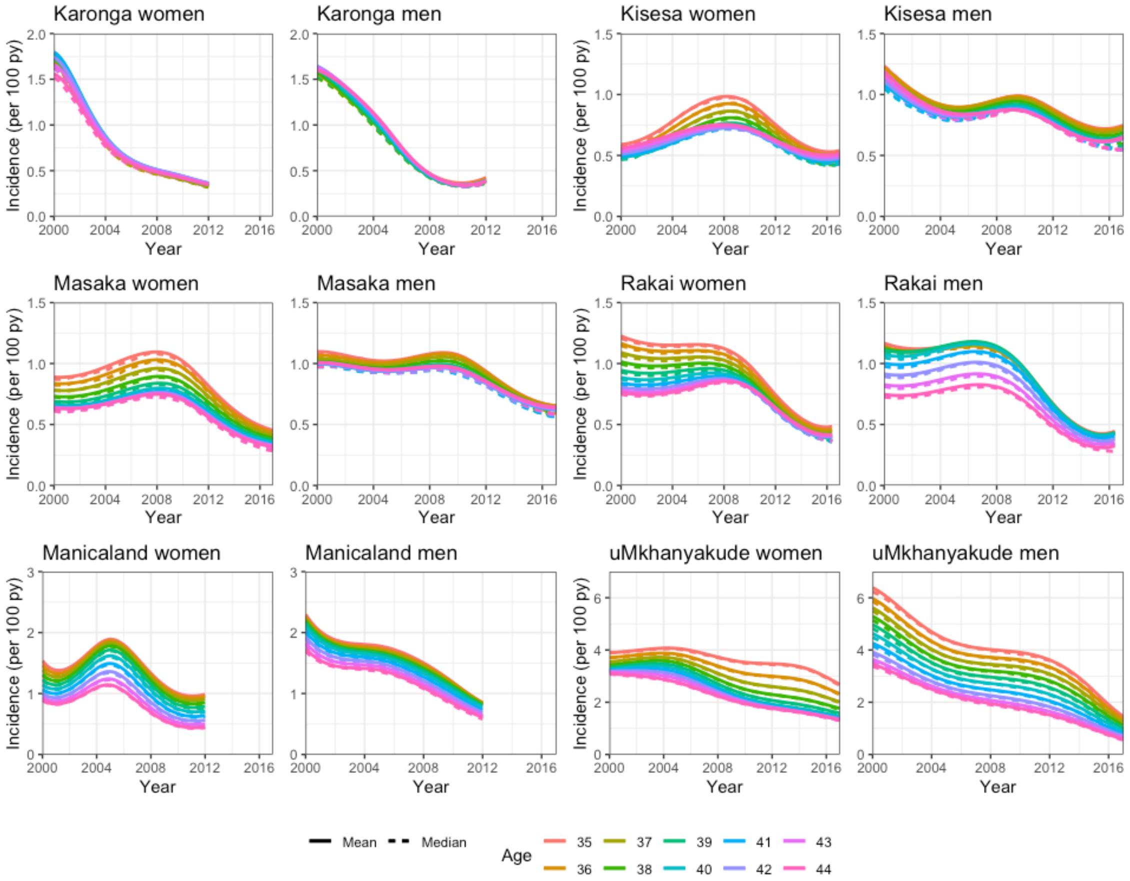

Figure S12: Estimated HIV incidence rate by single age 35-44.

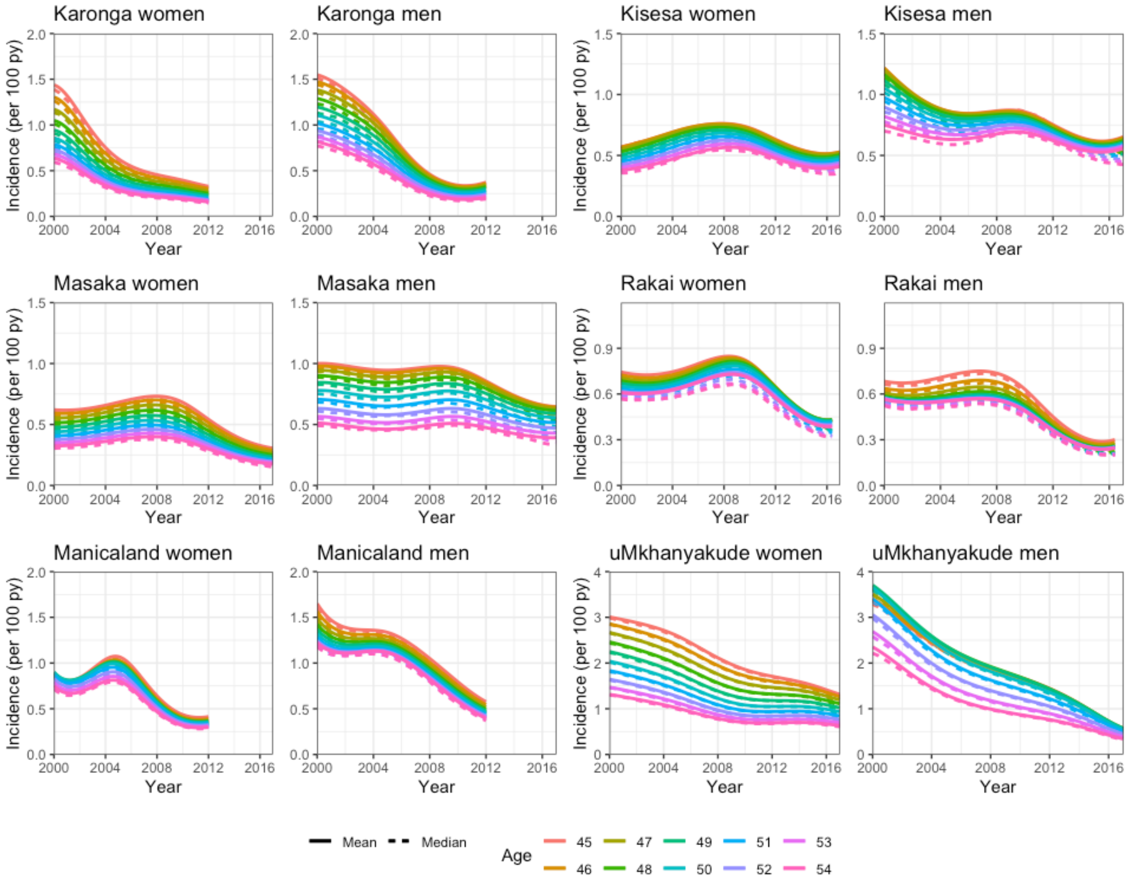

Figure S13: Estimated HIV incidence rate by single age 45-54.

S2.6 HIV prevalence by age

Figure S14 shows the estimated HIV prevalence by 10-year age-group from 2000-2017. Across studies and sexes, we largely see an increasing prevalence in ages 45-54 and flat or decreasing prevalence among 15-24 year olds. In uMkhanyakude, prevalence in ages 25-34 continues to increase for women, but in all other studies and sexes these ages see decreasing prevalence. Among 35-44 year olds, prevalence increases over the period in uMkhanyakude, and women in all other studies, while men outside of uMkhanyakude show either stable or decreasing prevalence in this age group.

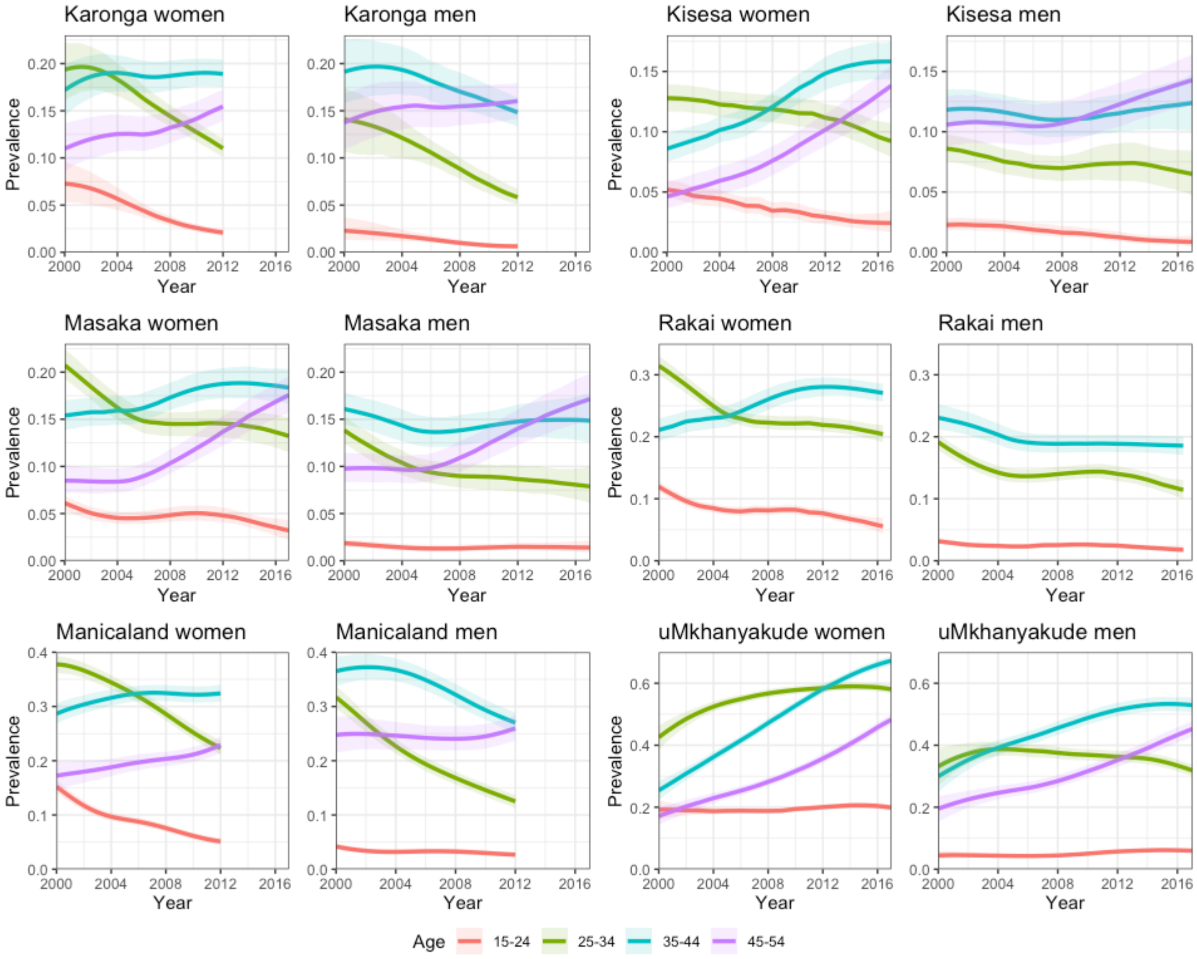

Figure S14: Estimated HIV prevalence by 10-year age group in 6 ALPHA network studies.

S2.7 Cumulative HIV incidence by birth cohort

Figure S15 shows the cumulative HIV incidence by birth cohort for individuals born in 1970, 1975, 1980, 1985, 1990, and 1995. When comparing cumulative incidence in adolescence and adulthood (starting at age 15) for successive birth cohorts in the studies, cohorts born more recently are on average experiencing reduced cumulative incidence across the life course. The cohort turning age 15 in 1985 had lower cumulative incidence in younger ages in the Southern African studies where the epidemic is inferred to have started later (uMkhanyakude, Manicaland and Karonga), where there was no or little HIV circulating in 1985 when this cohort turned 15. Cumulative incidence in men remains low until age 20 or so across all studies, while in women it starts to increase younger. Among uMkhanyakude women, consistent with the late (2014) decrease in overall incidence, each cohort's cumulative incidence only began to decline in the three most recent years.

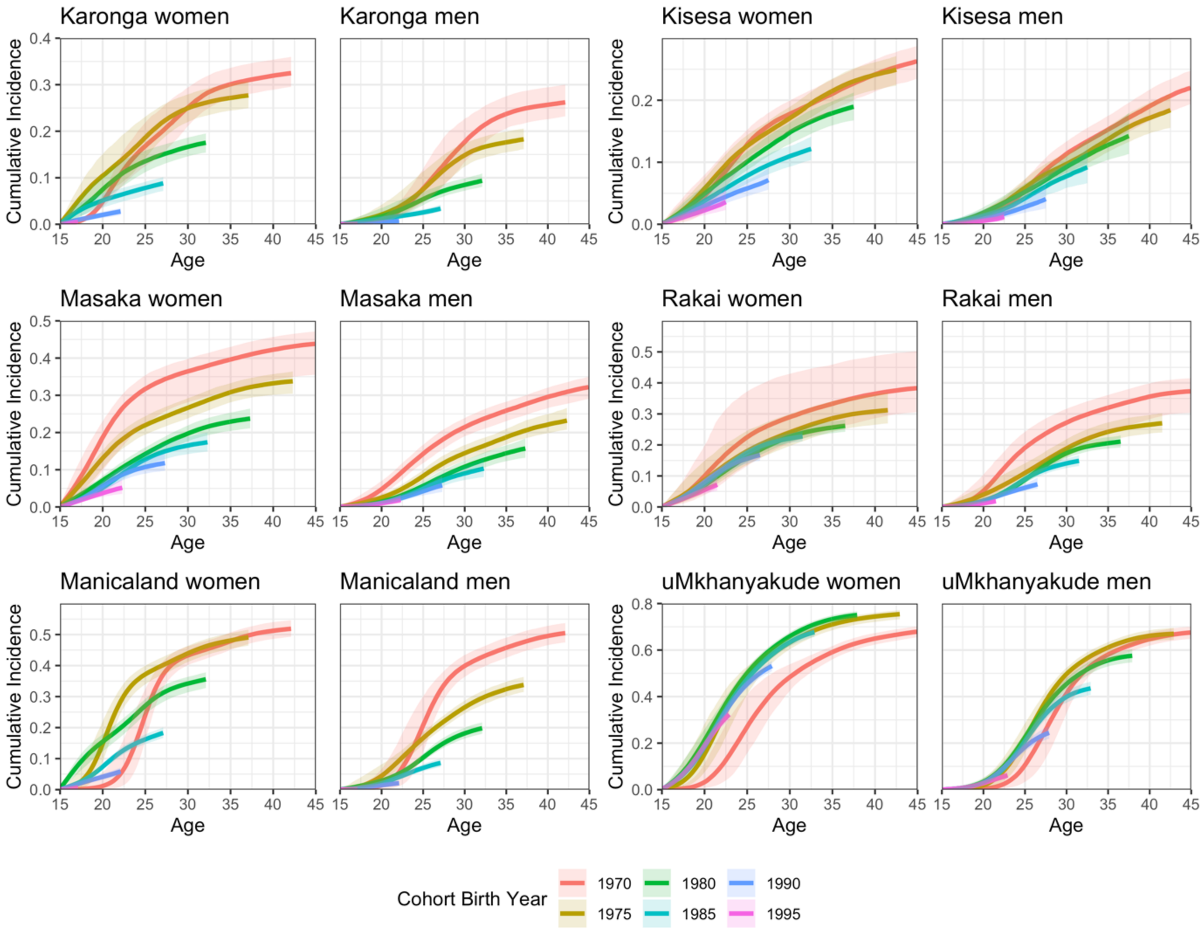

Figure S15: Cumulative HIV incidence probability by age for successive birth cohorts.

S2.8 Sex ratio in incidence projections

Figure S16 shows the sex ratio of birth cohort and period cumulative incidence in the two projection scenarios presented in the main text Figure 5. The projection scenarios are: 1) future age-specific incidence rates remain constant at the most recent estimated levels (2012 in Karonga and Manicaland and 2017 in other settings), and 2) that the age-specific HIV incidence rates continue to decline at the same study- and sex-specific rate estimated for the past 5 years among ages 15-54 years, with sex-specific declines converging in 2022, after which the rate of decline is assumed to be the same in both sexes, to avoid strongly imbalanced sex ratio in projections.

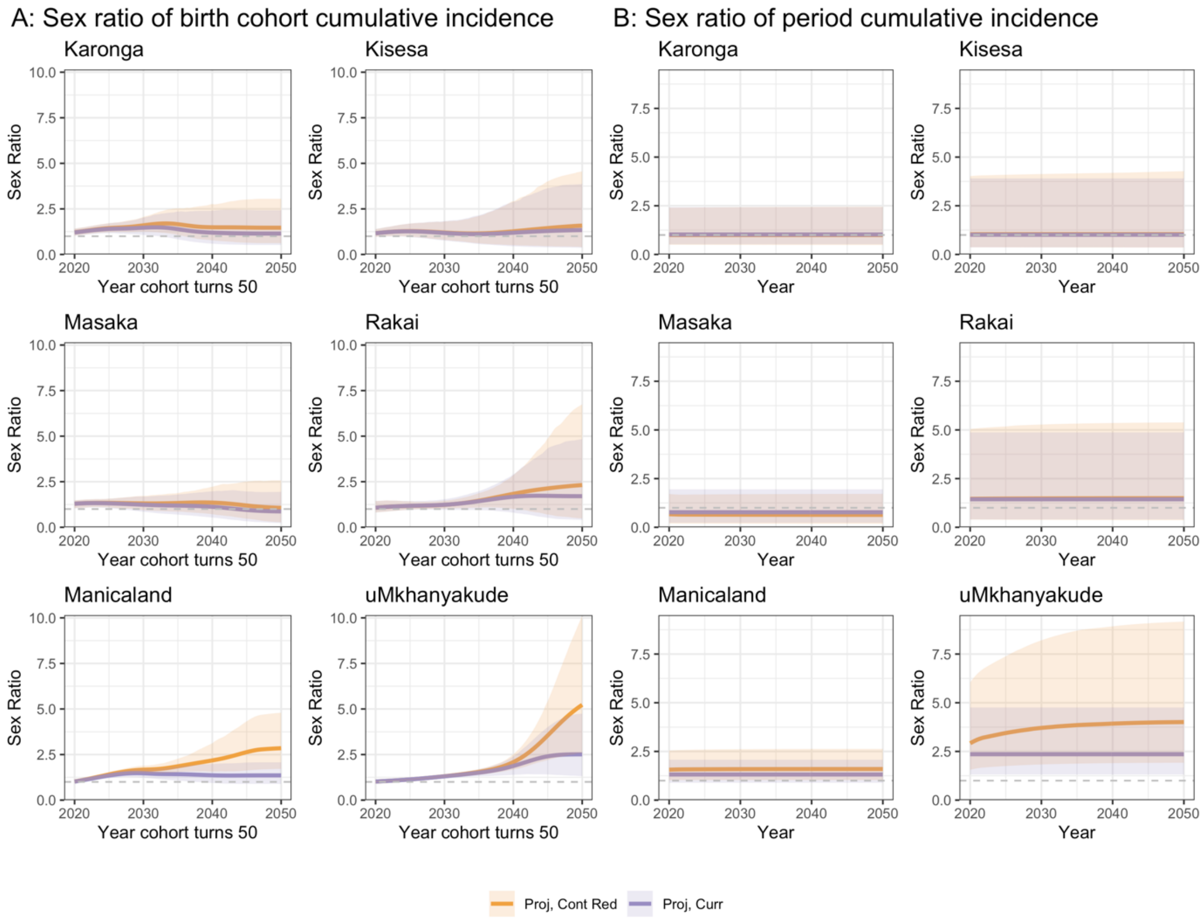

Figure S16: Sex ratio (women:men) for incidence projections showing A) ratio of birth cohort cumulative incidence and B) ratio of period cumulative incidence. Projected assuming current period age-specific incidence (Proj. Curr), and projected assuming continued relative reduction observed in the past five years (Proj. Cont Red). Dashed horizontal grey line at 1 (equality between the sexes). (N.B. panel A and B have different vertical axis ranges).

S2.9 Population pyramid of HIV population

Figure S17 shows the study population age structure by sex over time disaggregated by modelled HIV prevalence and incidence. The estimated average age of new infections is denoted by horizontal dashed lines. The population pyramids demonstrate that these adult populations have a high density of younger adults, with a very large 15-19 year old age group in Masaka, Kisesa and uMkhanyakude, in particular. uMkhanyakude and Kisesa have previously documented high out-migration in young ages.[6,7] Again, the Manicaland population, due to incomplete census in the study area, is standardised to the national population, and as such shows substantially more balance across ages.

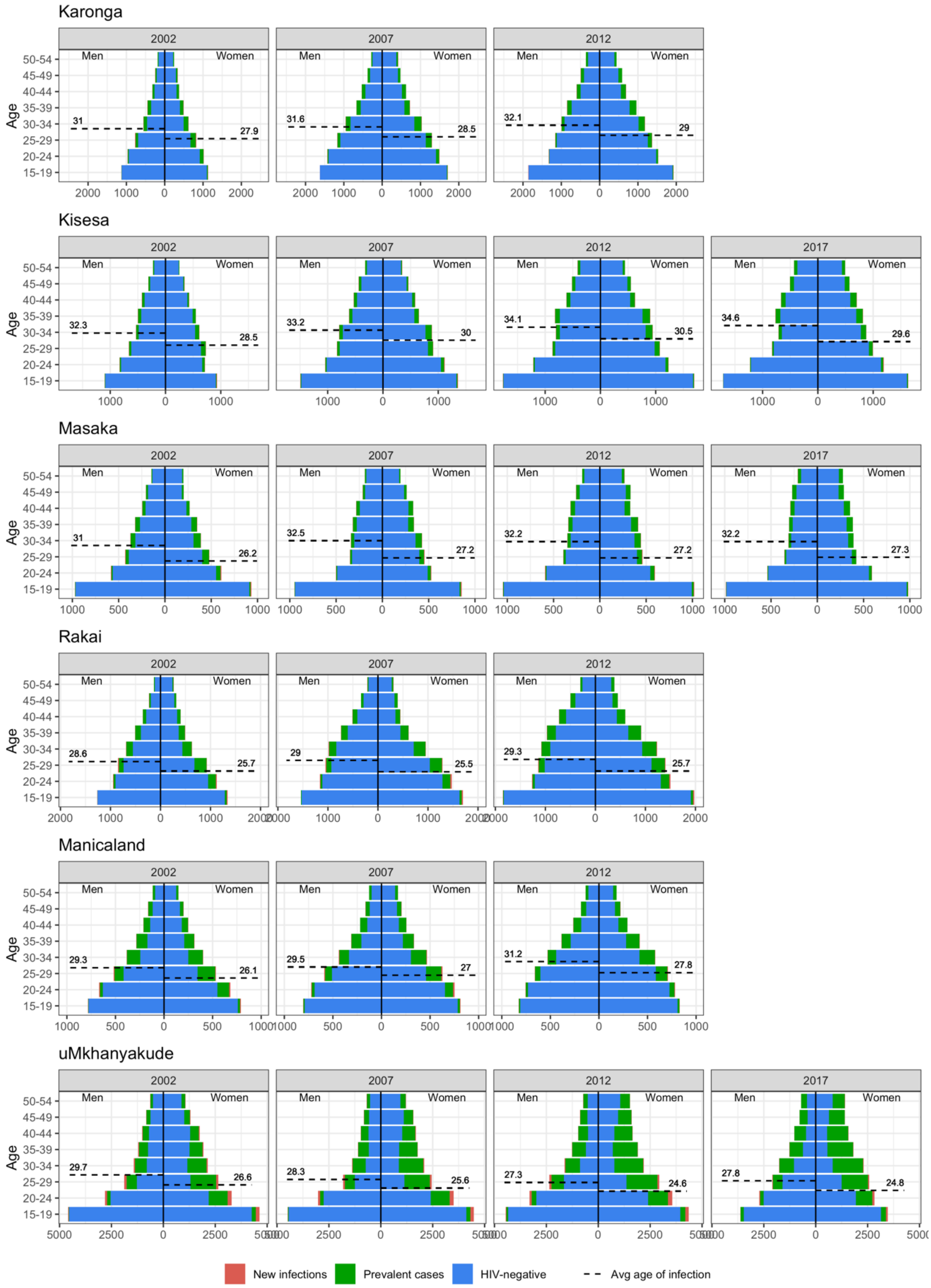

Figure S17: Population pyramids showing modelled number susceptible, prevalent and new HIV cases by 5-year age group in years 2002, 2007, 2012 and 2017. Horizontal dashed line denotes the estimated average age of new HIV infection. "New infections" reflects new infections in the prior 1-year period, not the entire 5-year period.

S3 Comparison to Direct Estimates

This section compares results of the joint model for HIV incidence and prevalence to direct estimates based on HIV serosurvey data in each study round.

S3.1 Age-specific HIV prevalence by serosurvey round

Figure S18 through Figure S26 present the model posterior predictive distribution for HIV prevalence for each HIV serosurvey round in each cohort study by sex and five-year age group. Black points represent the observed HIV prevalence based on the mean prevalence among all participants in the age group at the time of the survey. Posterior predictive distributions were by generated by sampling synthetic observations based with the same composition of age and testing dates of study participants for each sample from the posterior distribution. The year listed is the midpoint of each testing round. Horizontal green bars represent the mean of the posterior predictive distribution. Green shaded areas represent 80% and 95% predictive ranges, respectively.

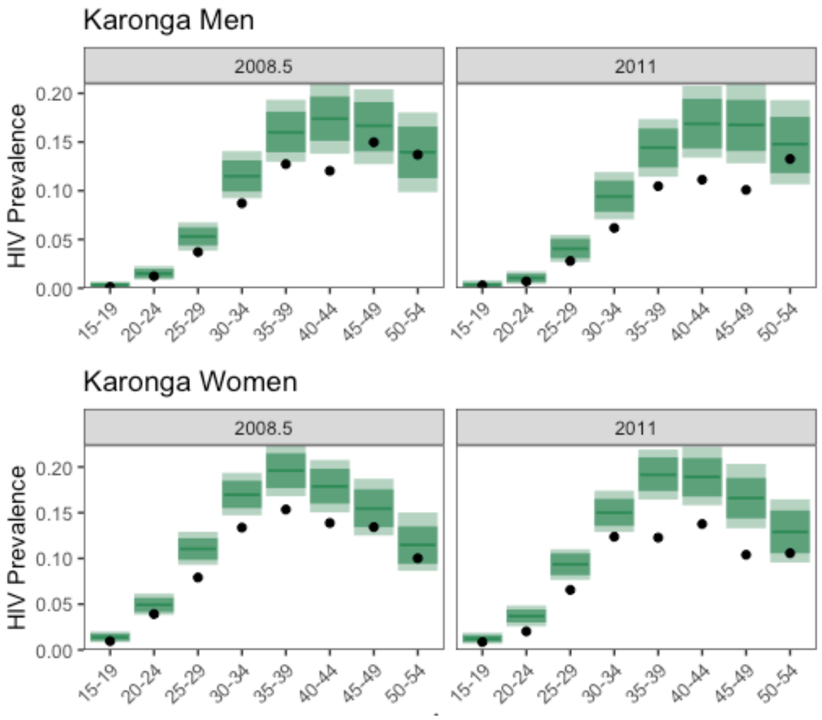

Figure S18: Posterior predictive distributions for age-specific prevalence in each survey round: Karonga Men and Women.

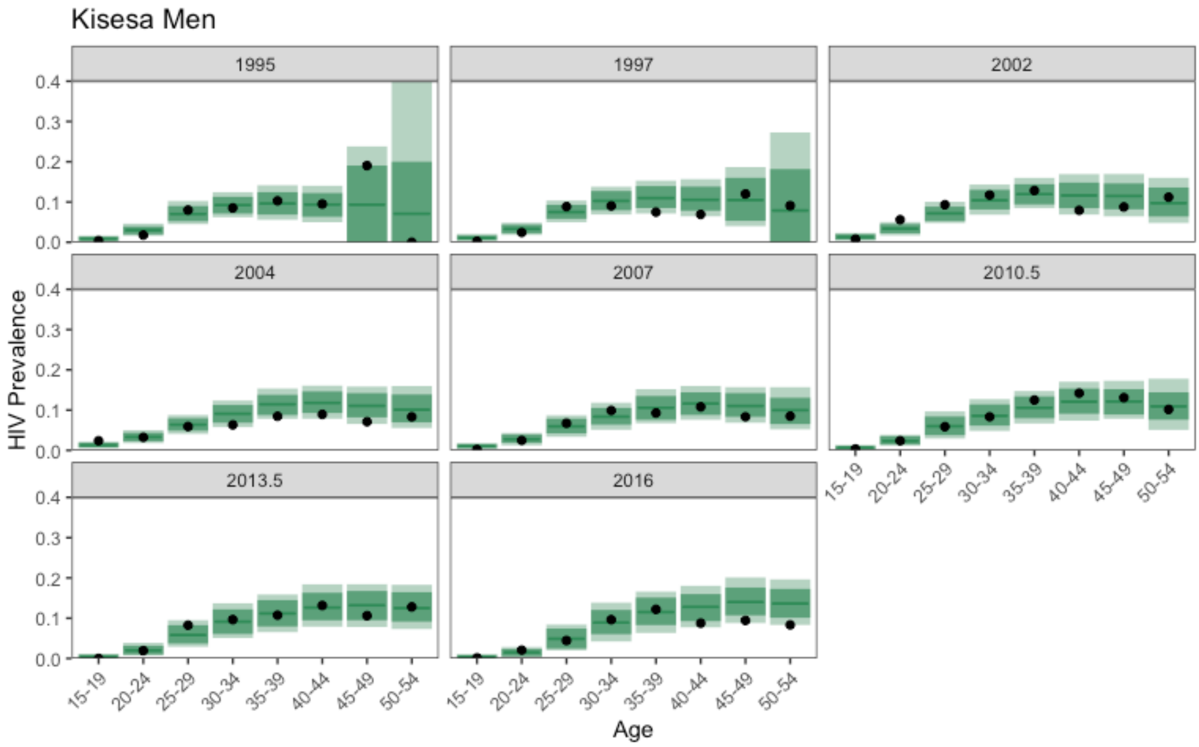

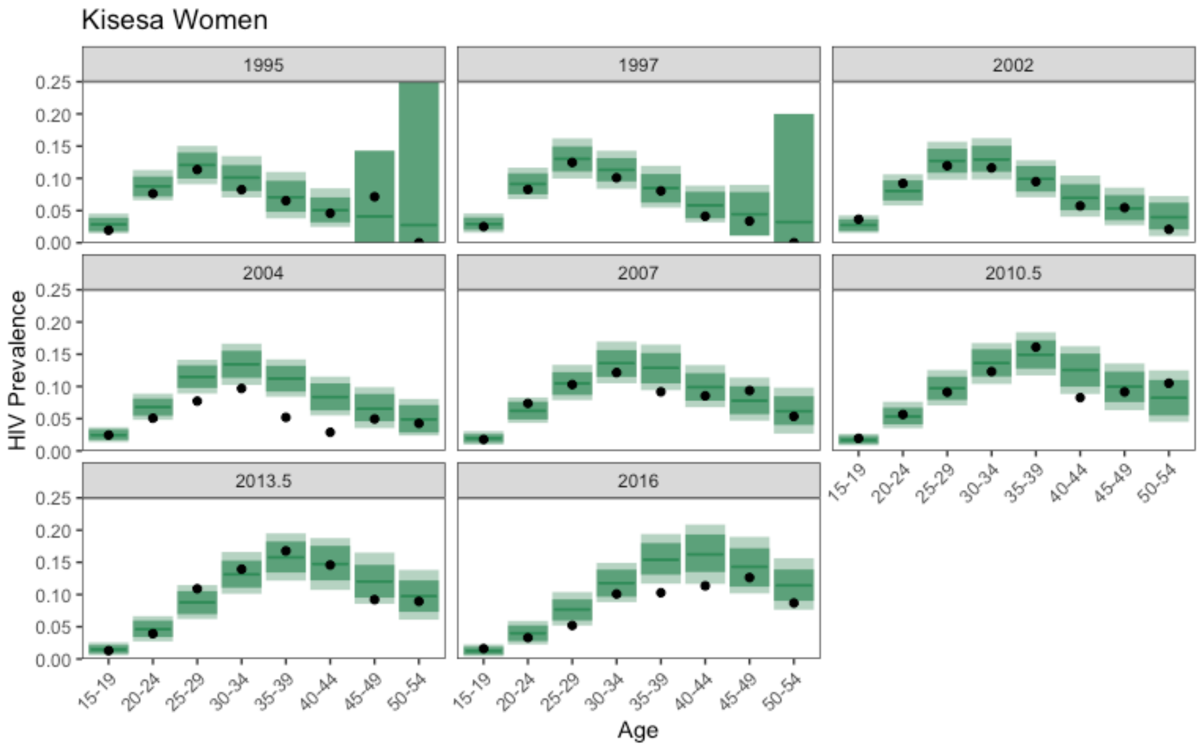

Figure S 19: Posterior predictive distributions for age-specific prevalence in each survey round: Kisesa Men and Women.

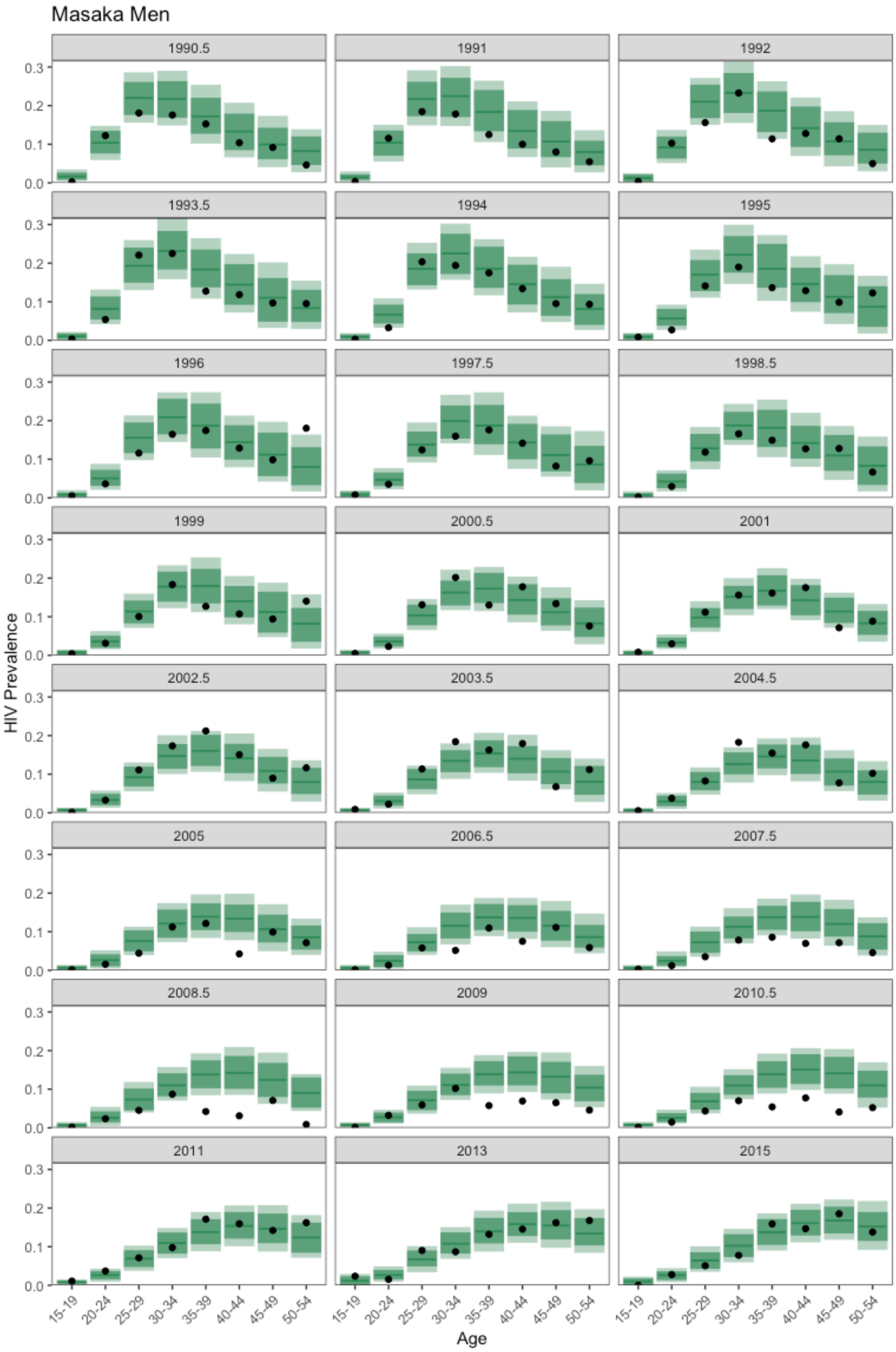

Figure S20: Posterior predictive distributions for age-specific prevalence in each survey round: Masaka Men.

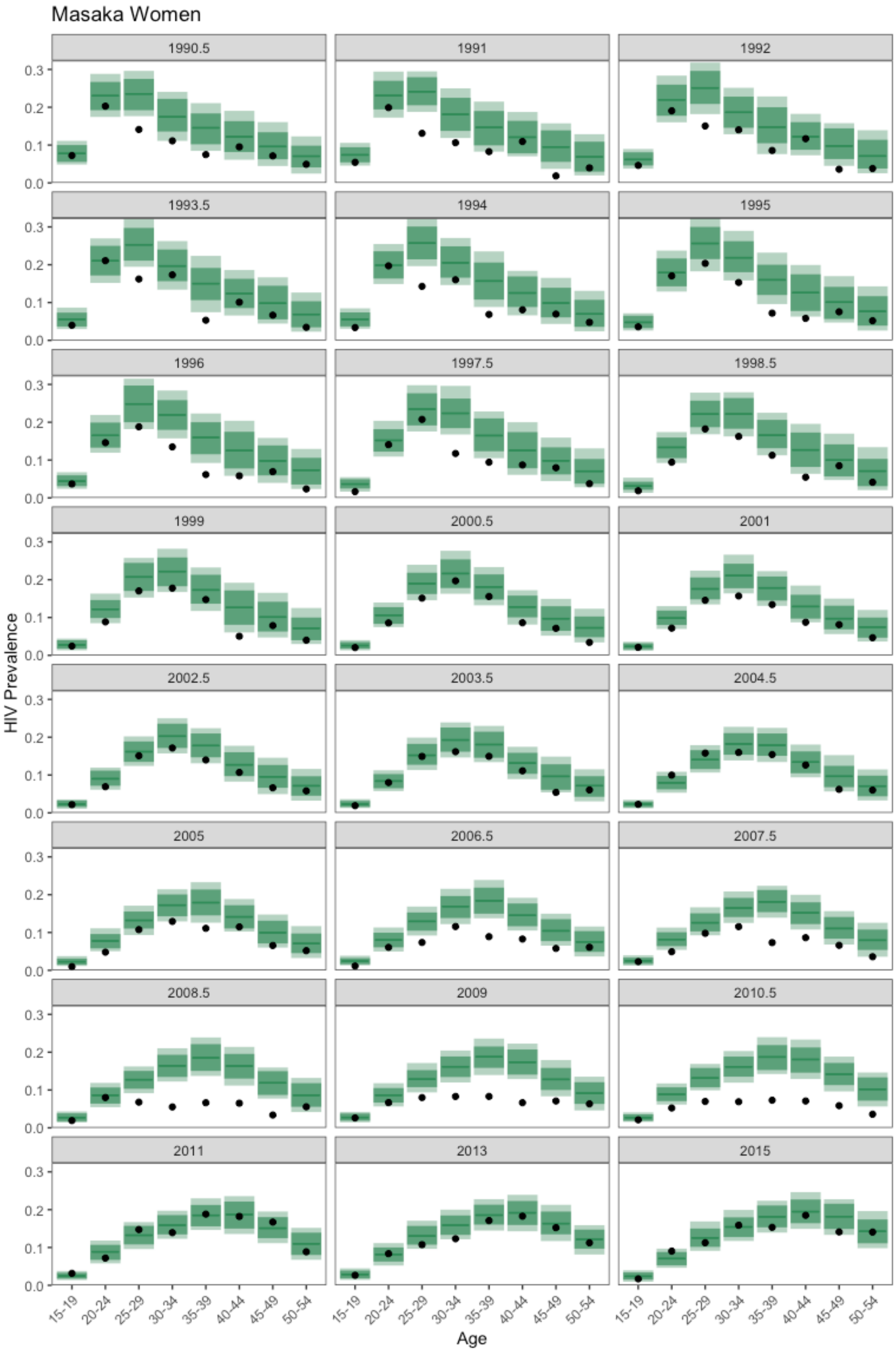

Figure S21: Posterior predictive distributions for age-specific prevalence in each survey round: Masaka Women.

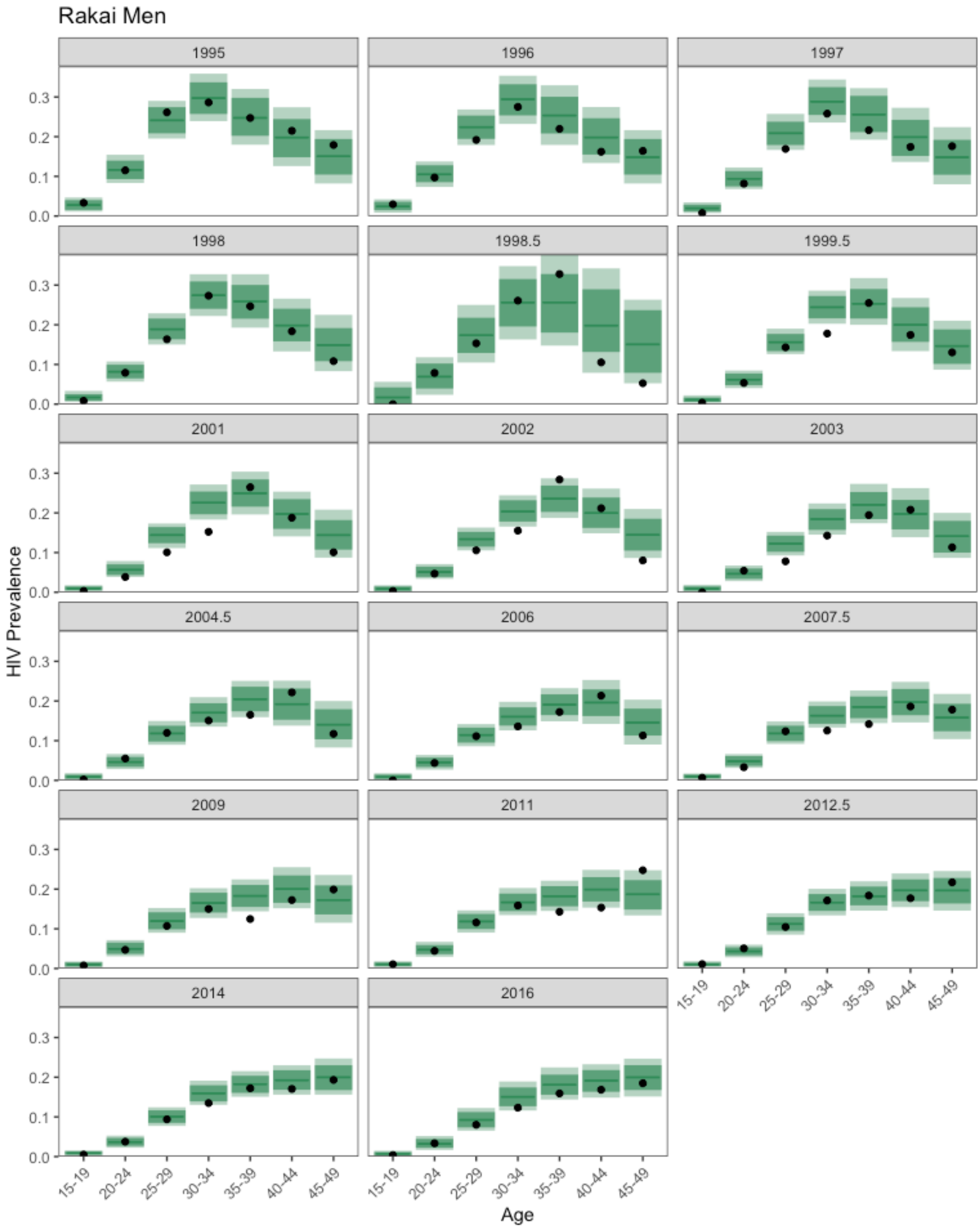

Figure S22: Posterior predictive distributions for age-specific prevalence in each survey round: Rakai Men.

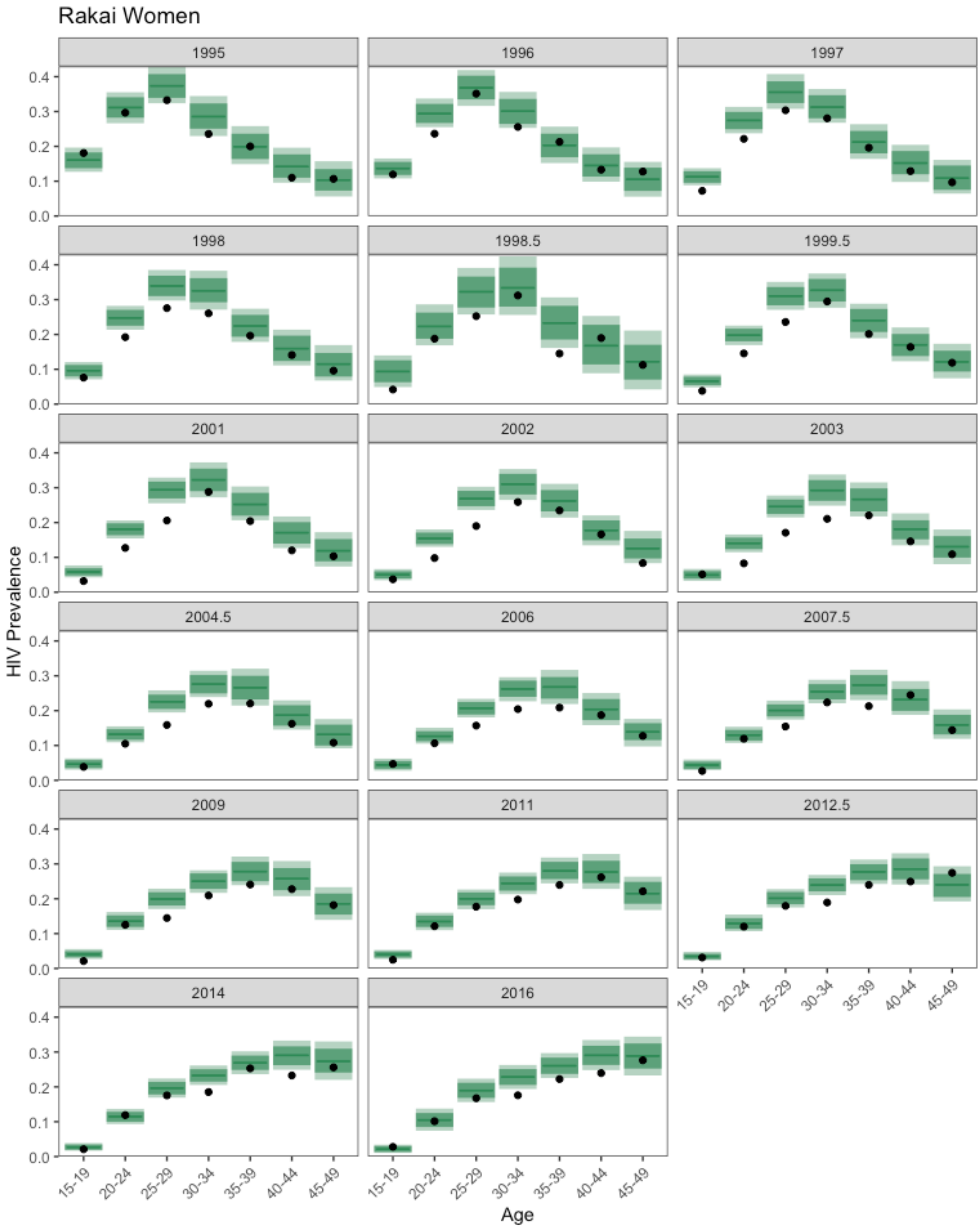

Figure S23: Posterior predictive distributions for age-specific prevalence in each survey round: Rakai Women.

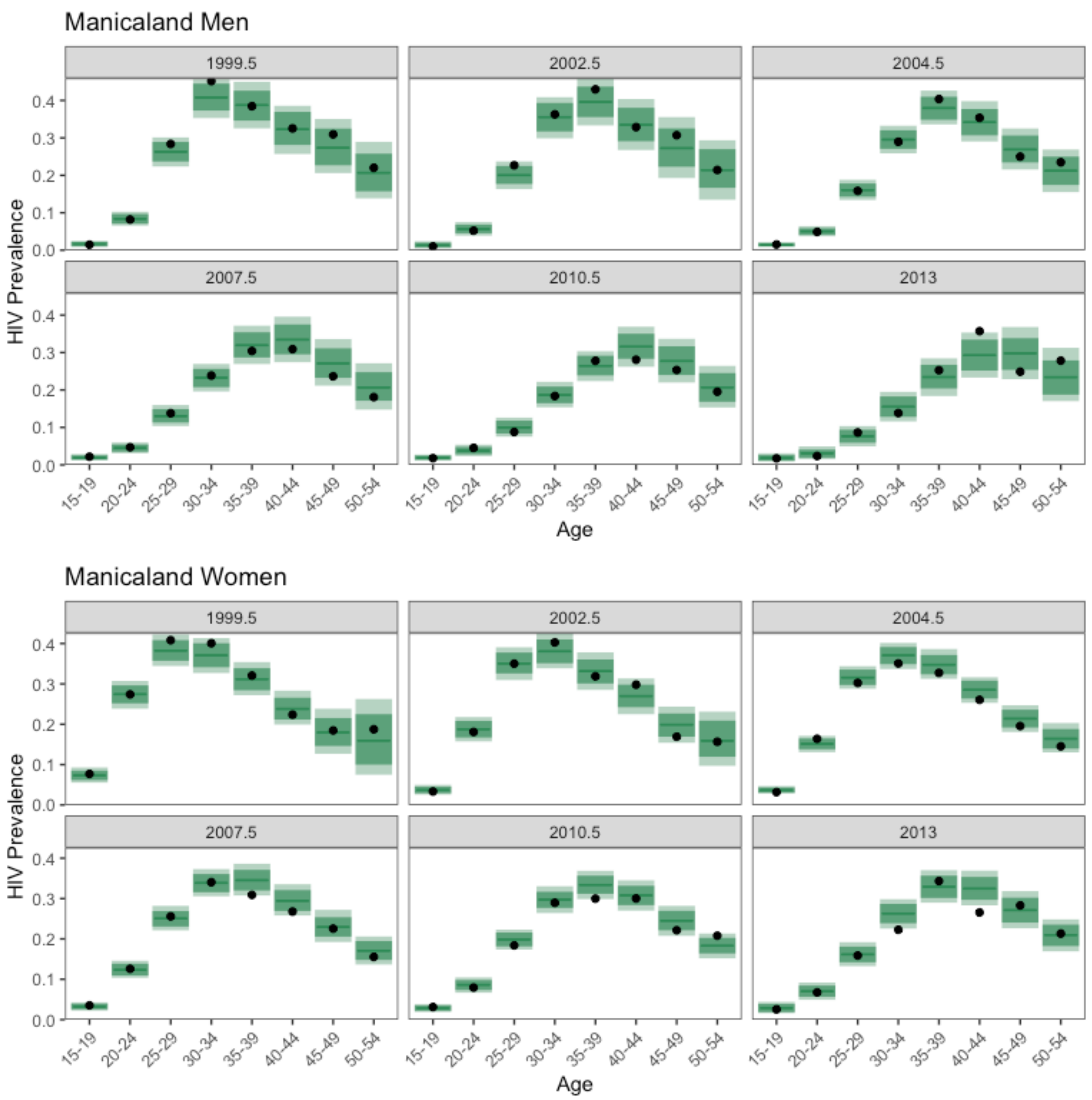

Figure S24: Posterior predictive distributions for age-specific prevalence in each survey round: Manicaland Men and Women.

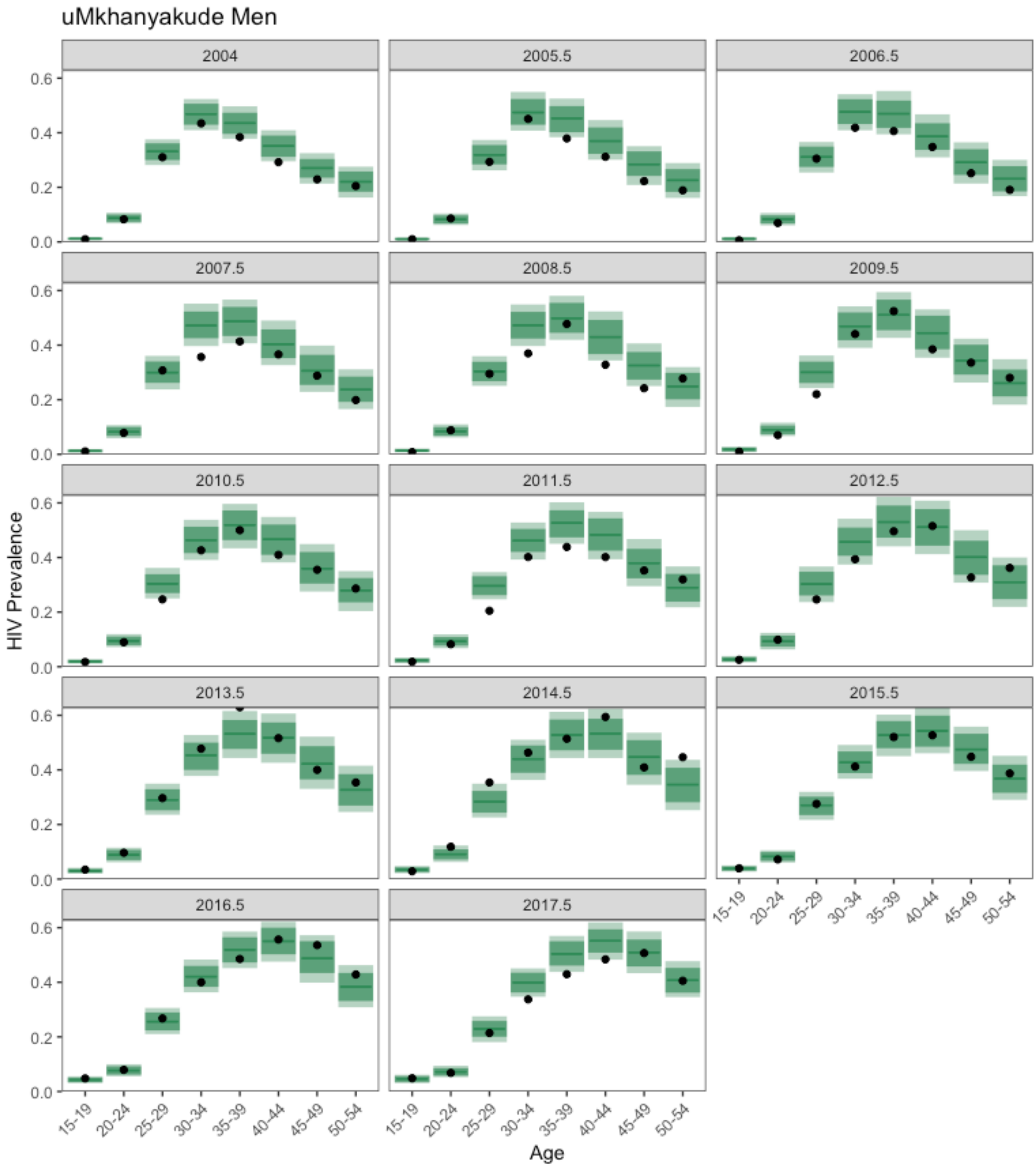

Figure S25: Posterior predictive distributions for age-specific prevalence in each survey round: uMkhanyakude Men.

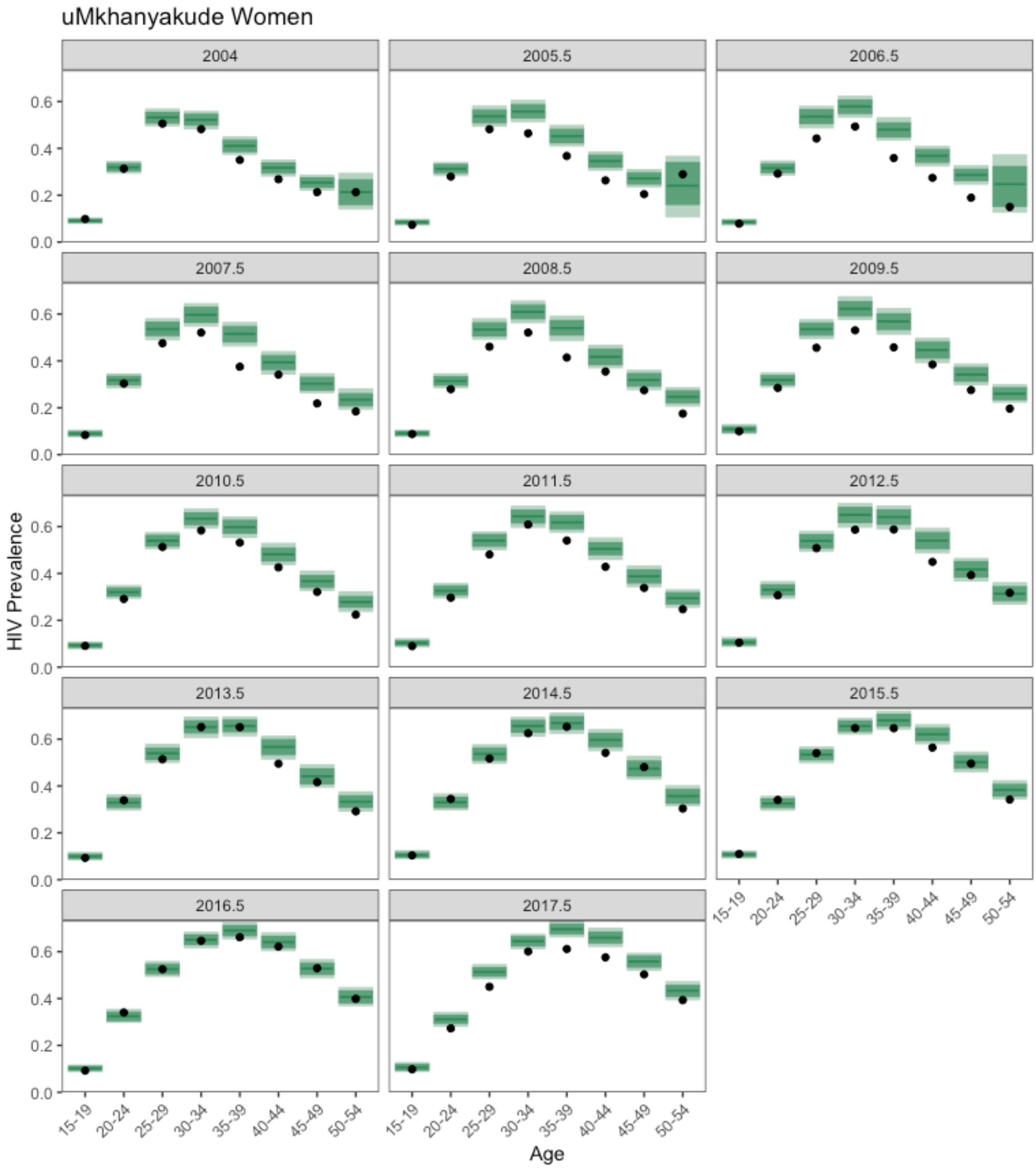

Figure S26: Posterior predictive distributions for age-specific prevalence in each survey round: uMkhanyakude Women.

S3.2 HIV incidence rate and cumulative incidence 15-54

Figure S27 compares the estimated trend in HIV incidence rate among age 15-54 to annual direct HIV incidence using only data among HIV seroconverters (black). Direct incidence estimates and 95% confidence intervals are results from pooling 70 random imputations of the date of HIV seroconversion date between the date of the last HIV negative and first HIV positive test.

Figure S28 compares cumulative incidence (lifetime risk of infection) from the model to cumulative incidence from the directly observed seroconverter cohort data. Period cumulative incidence direct estimates (black) were calculated based on Kaplan-Meier estimates averaged over 70 seroconversion date imputations.

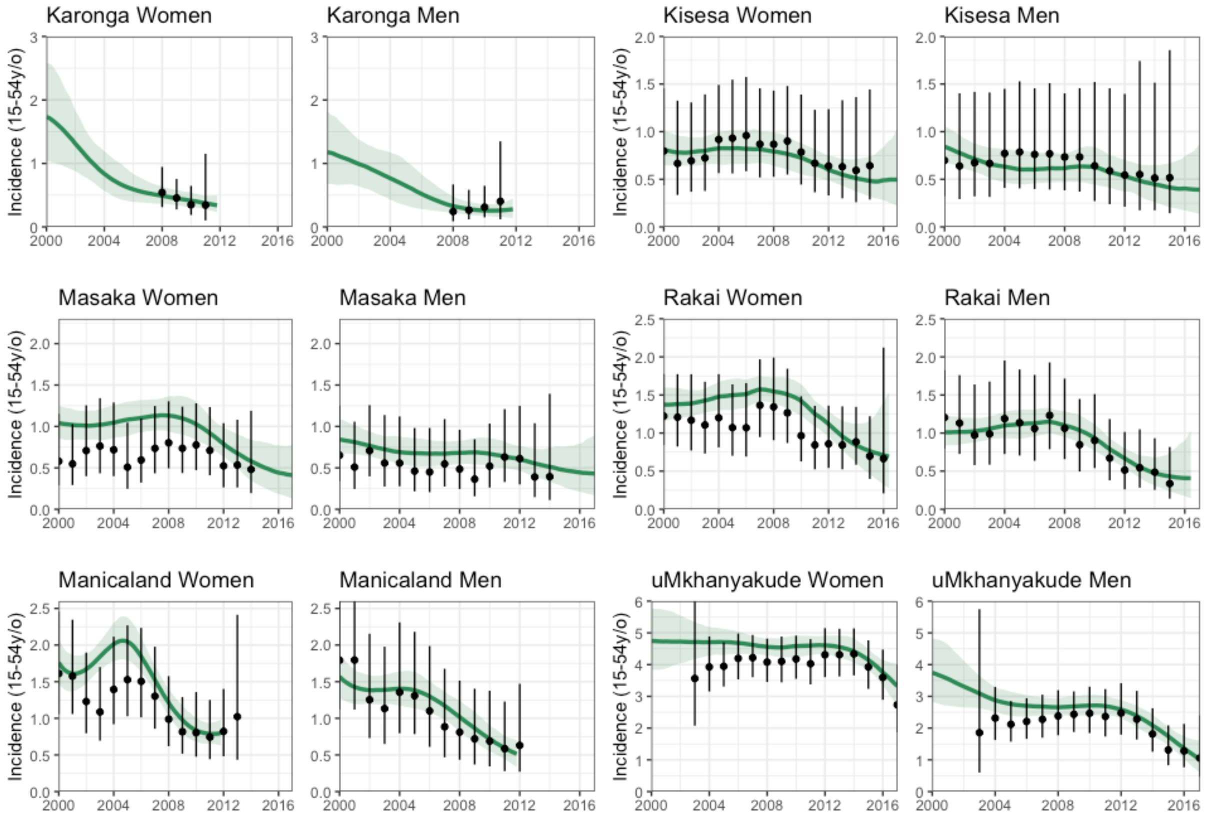

Figure S27: Incidence in ages 15-54 comparing modelled ALPHAEpi (green) to empirical estimates (black).

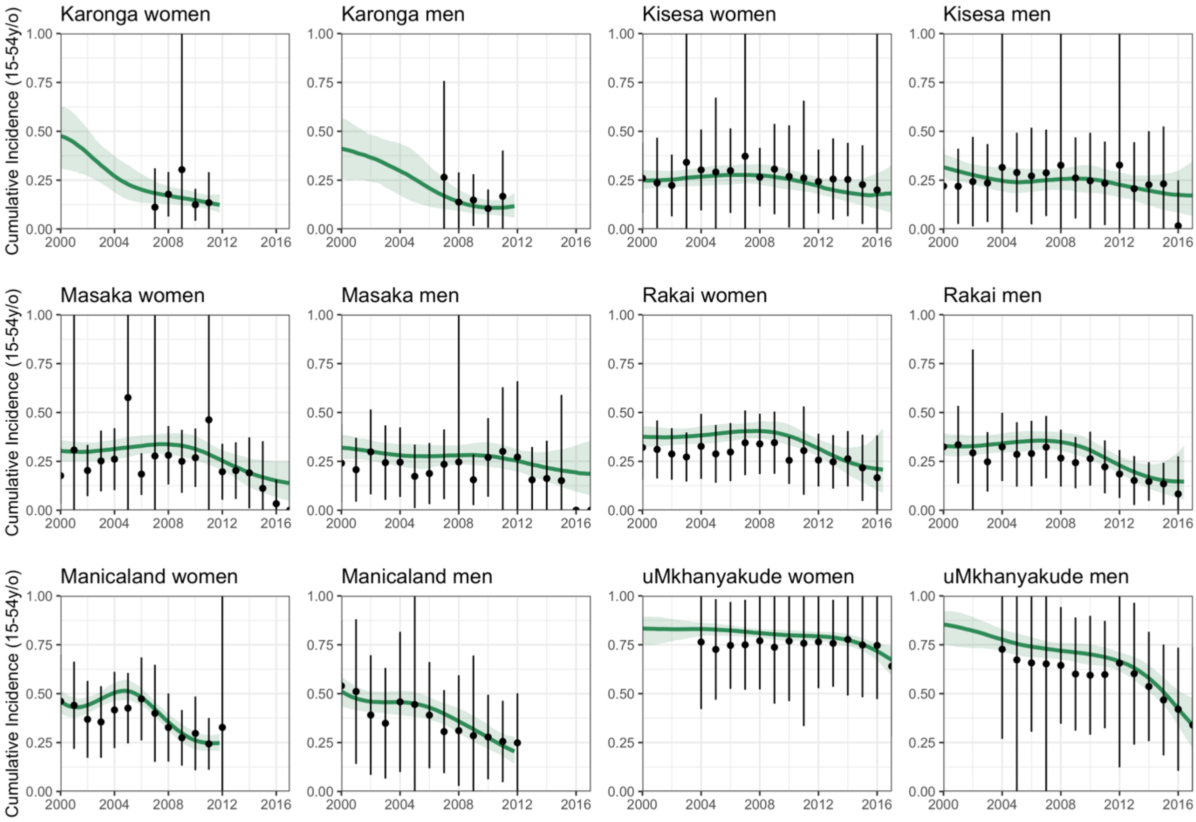

Figure S28: Cumulative incidence from ages 15-54 comparing modelled ALPHAEpi (green) to empirical estimates (black).

S3.3 Mean age of HIV infection

Figure S29 compares model estimates for the average age of HIV infection over time ('ALPHAEpi'; red line) with (1) a crude average of the age at infection among observed seroconverters in each study round ('Seroconverters', black line), and (2) estimates of the average age of infection based on fitting a Bayesian generalized additive model to seroconverter data only (*i.e.* excluding seroprevalent and survival data incorporated in ALPHAEpi; "BG"; blue lines). The average age of infection from the Bayesian GAMs applied to the seroconverter cohort is similar to that estimated using the joint model. The empirical average age at observed seroconversion, in contrast, was higher than the modelled average age of infection due to the overrepresentation of older ages among participants in the seroconverter cohort relative to the enumerated household population in the study area.

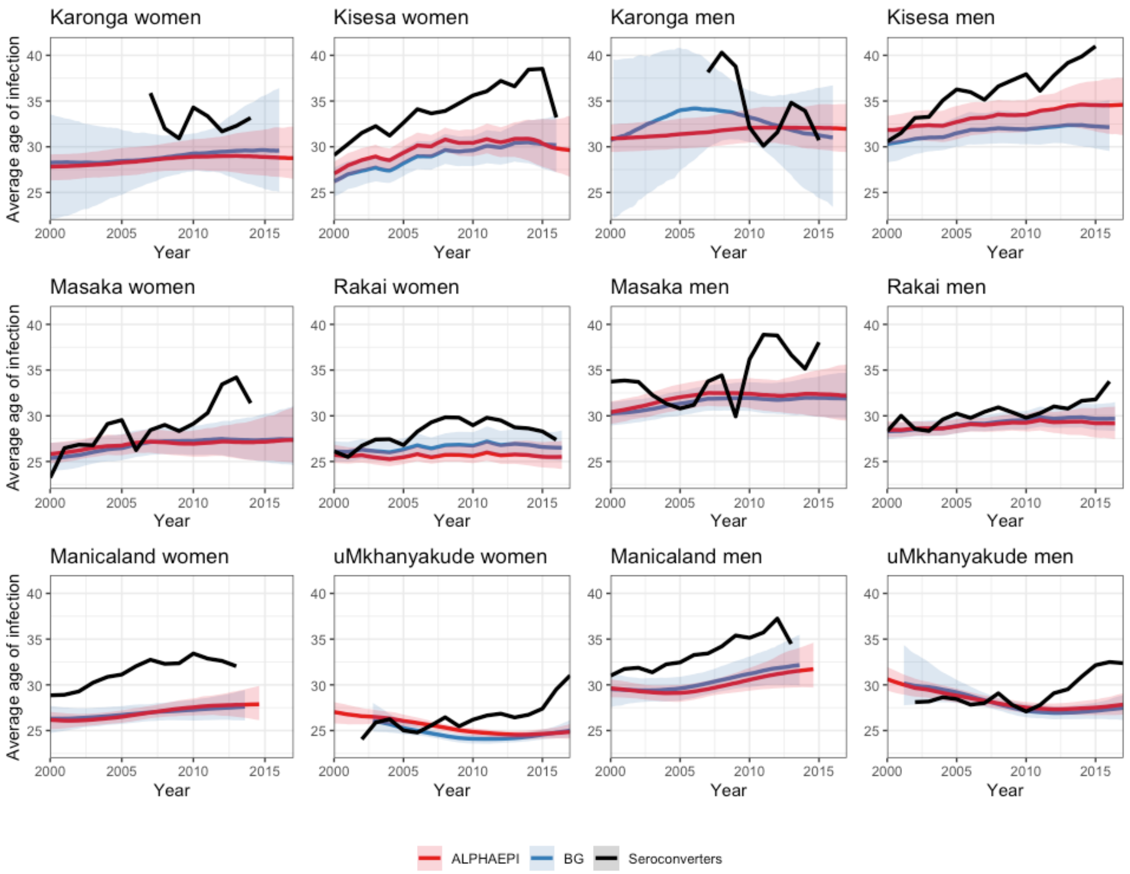

Figure S29: Average age of infection comparing results from the ALPHAEpi model, seroconverter cohort Bayesian GAMs (BG), and average age of seroconverters in 70 imputed datasets.

S4 Sensitivity Analyses

S4.1 National reference populations

The first sensitivity analysis investigated the consequences of standardising age-specific incidence and prevalence estimates to the population study distribution compared to the national population distribution. Figure S30 compares the pyramid of the resident population in each study area to the national population distribution (UN World Population Prospects 2019 Revision). For Manicaland, we only present results standardised to the national population of Zimbabwe because the Manicaland study does not conduct continuous demographic surveillance. These studies are rural areas with high out-migration among young men (and sometimes women). This is reflected by a larger proportion of the population age 15-19 years and smaller proportion among age 20-39 years compared to the national population

To assess the impact of standardising to the study population, Figure S31 shows estimates for the average age of HIV infection when standardised to the national population and Figure S32 shows the age distribution of new infections over time. The average age of infection results were similar overall whether standardising to the national population or the study population. The average age of infection was slightly lower when standardising to the national population, except for uMkhanyakude where it was slightly higher.

The proportion of infections occurring in different age groups is similar when standardised to the national population as when standardised to the study populations (Figure S32). Using the national standard, the proportion of women's infections occurring among 15-24 year olds, is similar for all studies. Among men, the proportions among 20-29 year old men in the most recent year are quite similar, except in Masaka where the proportion was somewhat higher (45%, 95% CI 32–56%).

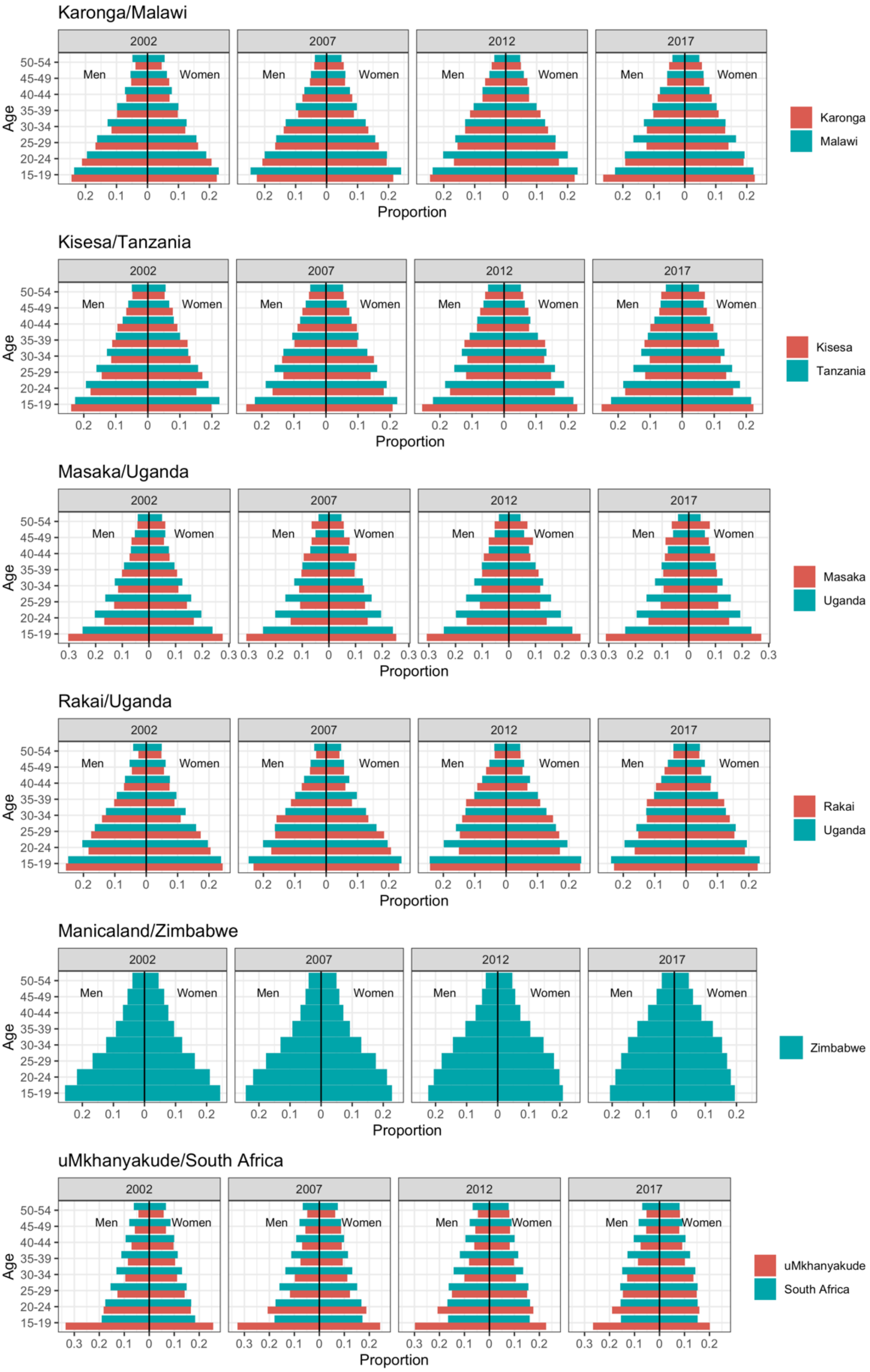

Figure S30: Comparison of the population resident in studies to the corresponding national populations (UN World Population Prospects 2019 revision) in 2002, 2007, 2012, and 2017.

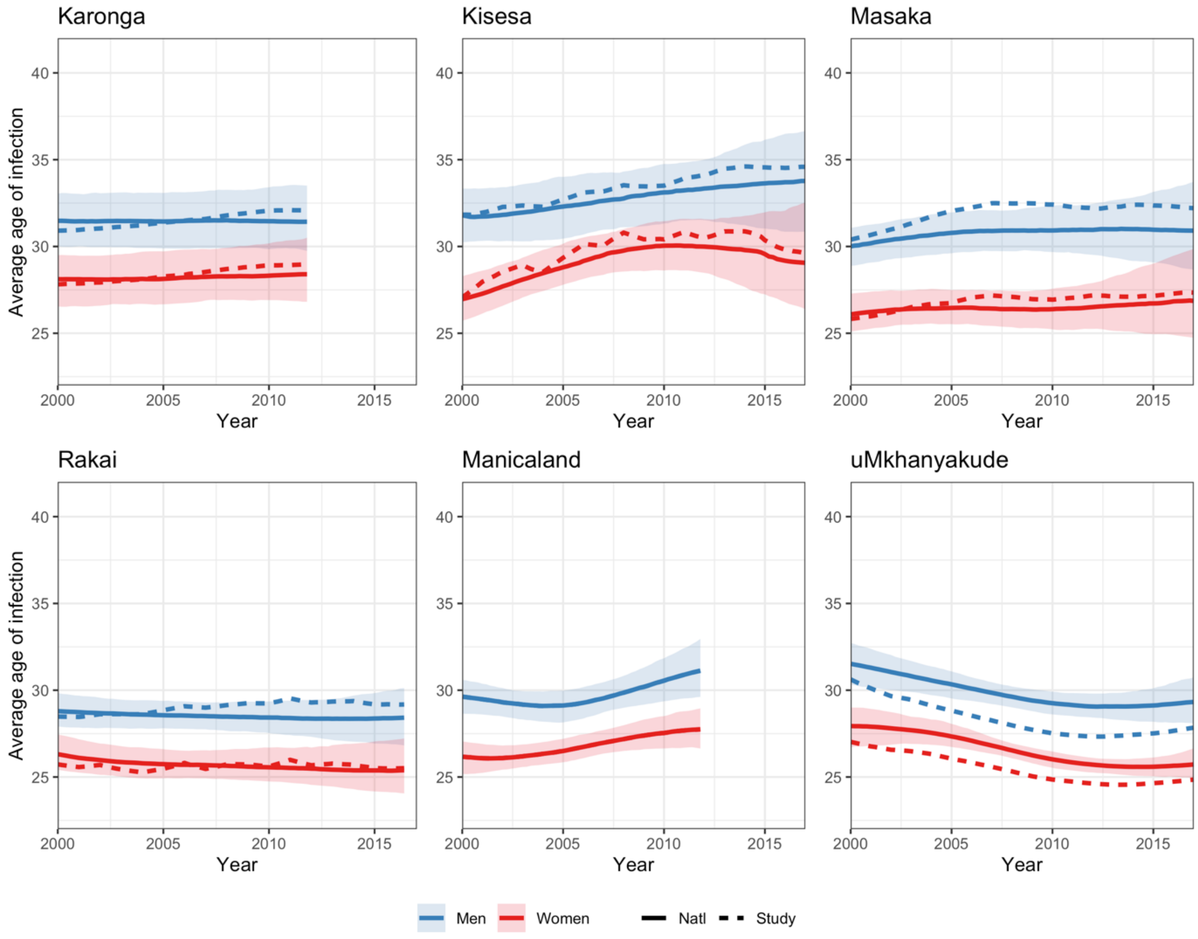

Figure S31: Average age of infection in six studies by sex over time, weighted with national population instead of study population. Dashed lines reflect estimates when weighted based on the study population distributions as presented in the primary results.

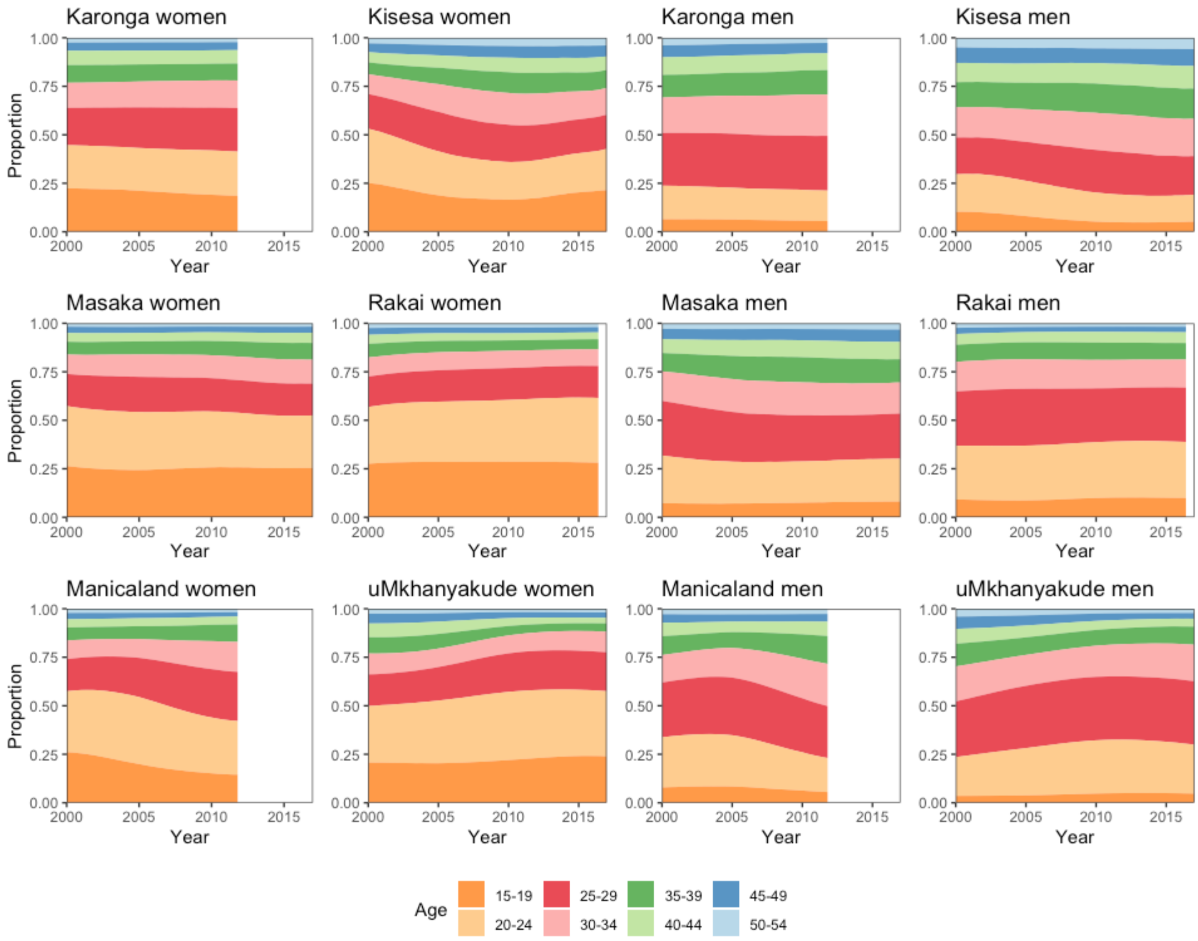

Figure S32: Proportion of infections in each five-year age group when standardised to national population distribution instead of study population distribution.

S4.2 Alternative specifications for $\tilde{\boldsymbol{\lambda}}(\boldsymbol{t}, \boldsymbol{a})$

In this analysis, we considered alternative specifications for the incidence hazard function:

- **Additive:** $\tilde{\lambda}(t, a) = f_t(t) + f_a(a)$
- **Isotropic:** $\tilde{\lambda}(t, a) = f_{ta}(t, a)$
- **Interaction:** $\tilde{\lambda}(t, a) = f_t(t) + f_a(a) + f_{ta}(t, a)$

The ‘interaction’ model was the specification presented in the primary results. The ‘additive’ model assumes that the age pattern of incidence is fixed over time. The ‘isotropic’ model allows for an interaction between age and time, but assumes equal smoothing in the age dimension and the time dimension. The interaction term (described in primary methods) assumes a main overall age pattern and time pattern, but allows an isotropically smoothed interaction for deviation from the main time and age trends.

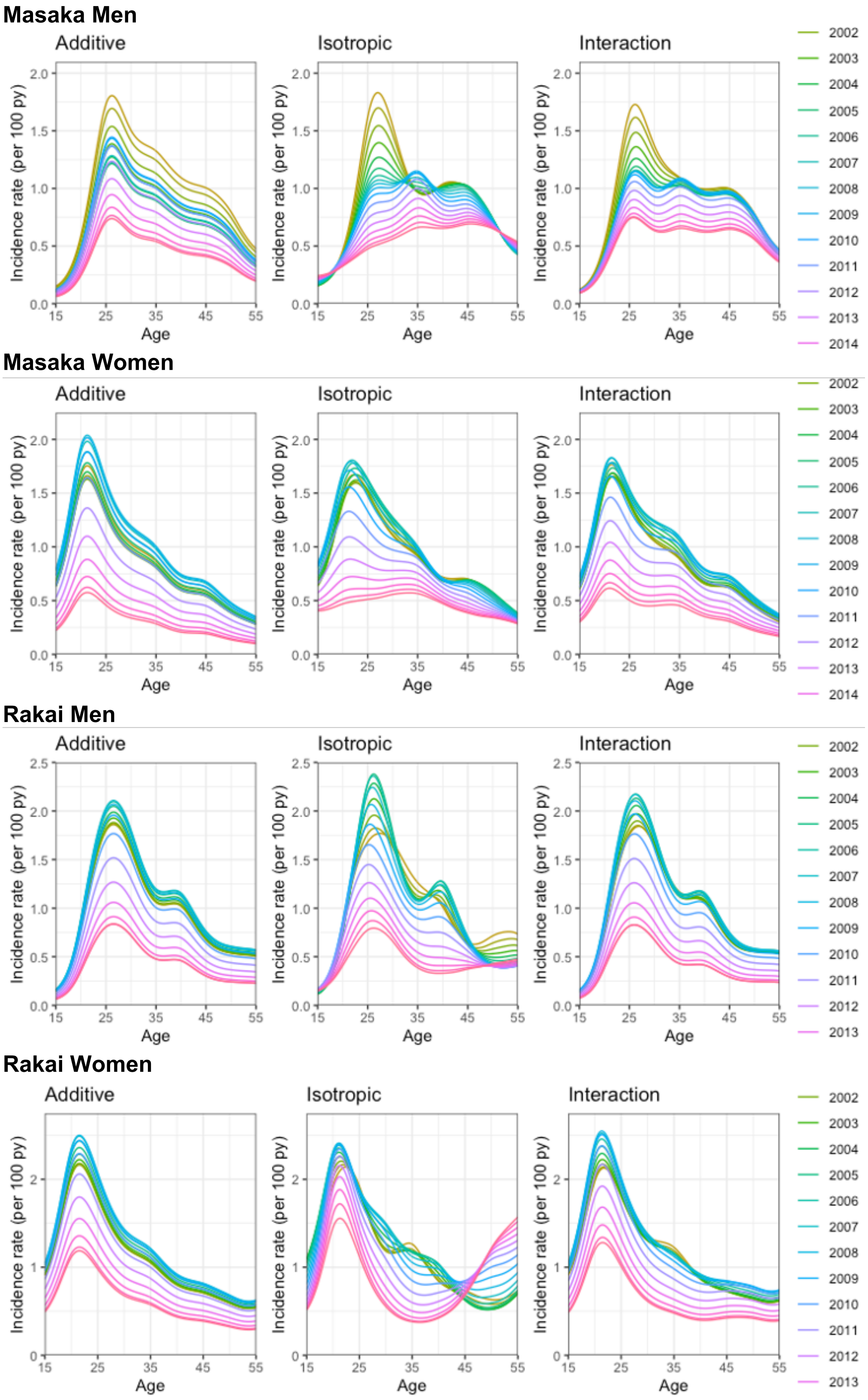

Figure S33: Age pattern of HIV incidence using 'additive', 'isotropic', or 'interaction' specification for $\tilde{\lambda}(t, a)$: Masaka and Rakai studies.

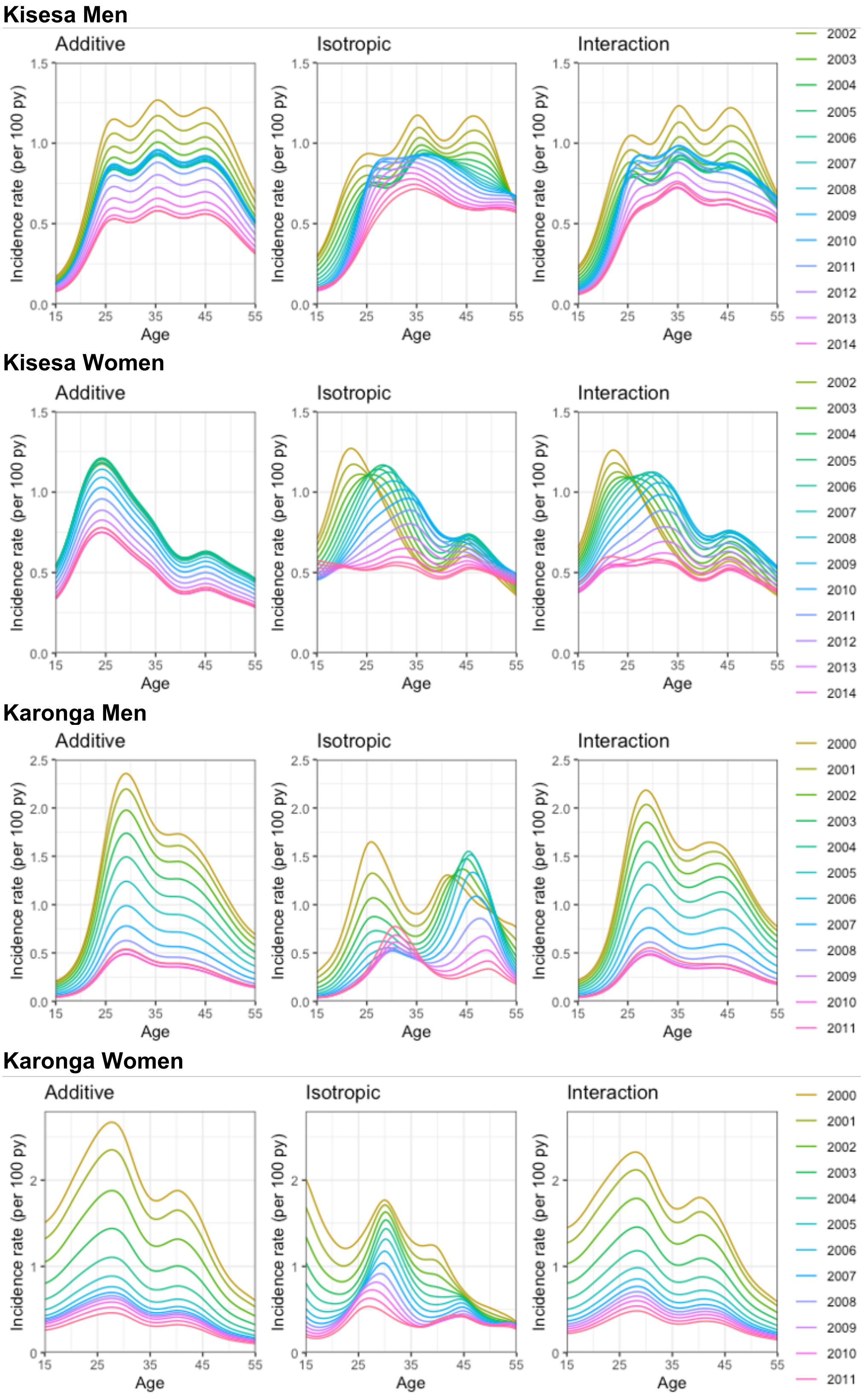

Figure S34: Age pattern of HIV incidence using 'additive', 'isotropic', or 'interaction' specification for $\tilde{\lambda}(t, a)$: Kisesa and Karonga studies.

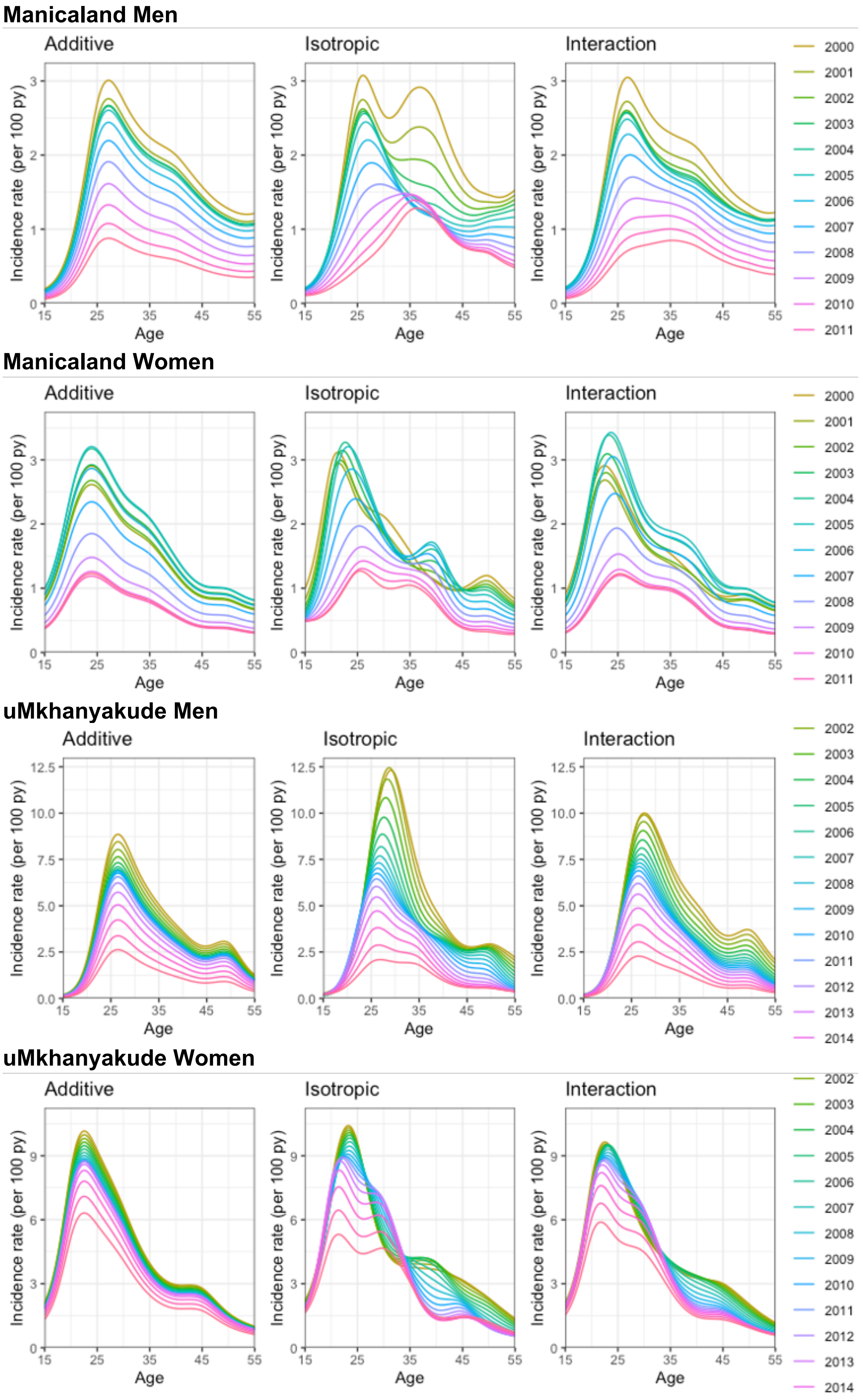

Figure S35: Age pattern of HIV incidence using 'additive', 'isotropic', or 'interaction' specification for $\tilde{\lambda}(t,a)$*: Manicaland and uMkhanyakude studies*

S5 STROBE statement

STROBE Statement—Checklist of items that should be included in reports of ***cohort studies***

	Item No	Recommendation	Page No
**Title and abstract**	1	(*a*) Indicate the study's design with a commonly used term in the title or the abstract<br>(*b*) Provide in the abstract an informative and balanced summary of what was done and what was found	a) Title<br>b) Abstract
**Introduction**			
Background/rationale	2	Explain the scientific background and rationale for the investigation being reported	Introduction, ¶ 1-4
Objectives	3	State specific objectives, including any prespecified hypotheses	Introduction, ¶ 4
**Methods**			
Study design	4	Present key elements of study design early in the paper	Methods, Data sources section
Setting	5	Describe the setting, locations, and relevant dates, including periods of recruitment, exposure, follow-up, and data collection	Methods, Data sources section ¶ 1, Table 1
Participants	6	(*a*) Give the eligibility criteria, and the sources and methods of selection of participants. Describe methods of follow-up<br>(*b*) For matched studies, give matching criteria and number of exposed and unexposed	a) Methods, Data sources section ¶ 1-2<br>b) N/A
Variables	7	Clearly define all outcomes, exposures, predictors, potential confounders, and effect modifiers. Give diagnostic criteria, if applicable	Methods, Model description ¶ 2
Data sources/ measurement	8*	For each variable of interest, give sources of data and details of methods of assessment (measurement). Describe comparability of assessment methods if there is more than one group	Methods, Data sources section ¶ 2
Bias	9	Describe any efforts to address potential sources of bias	Methods, Model description section, ¶ 1
Study size	10	Explain how the study size was arrived at	Methods, Data sources section ¶ 1
Quantitative variables	11	Explain how quantitative variables were handled in the analyses. If applicable, describe which groupings were chosen and why	N/A
Statistical methods	12	(*a*) Describe all statistical methods, including those used to control for confounding	a) Methods, Model description and Analyses sections

		(*b*) Describe any methods used to examine subgroups and interactions	b) Methods, Model description section ¶ 2-3
		(*c*) Explain how missing data were addressed	c) Methods, Model description ¶ 1
		(*d*) If applicable, explain how loss to follow-up was addressed	d) Methods, Model description ¶ 1
		(*e*) Describe any sensitivity analyses	e) Appendix Section S4
**Results**			
Participants	13*	(a) Report numbers of individuals at each stage of study—eg numbers potentially eligible, examined for eligibility, confirmed eligible, included in the study, completing follow-up, and analysed	a) Table 1 and Table S1 and S3-4
		(b) Give reasons for non-participation at each stage	b) Described in references 5, 14-18
		(c) Consider use of a flow diagram	c) N/A
Descriptive data	14*	(a) Give characteristics of study participants (eg demographic, clinical, social) and information on exposures and potential confounders	a) Table 1, Table S1-S4
		(b) Indicate number of participants with missing data for each variable of interest	b) N/A, approach utilises all available data
		(c) Summarise follow-up time (eg, average and total amount)	c) Table 1
Outcome data	15*	Report numbers of outcome events or summary measures over time	Results
Main results	16	(*a*) Give unadjusted estimates and, if applicable, confounder-adjusted estimates and their precision (eg, 95% confidence interval). Make clear which confounders were adjusted for and why they were included	a) Results
		(*b*) Report category boundaries when continuous variables were categorized	b) N/A
		(*c*) If relevant, consider translating estimates of relative risk into absolute risk for a meaningful time period	c) N/A
Other analyses	17	Report other analyses done—eg analyses of subgroups and interactions, and sensitivity analyses	Appendix, sections S2-4
**Discussion**			
Key results	18	Summarise key results with reference to study objectives	Discussion ¶ 1

Limitations	19	Discuss limitations of the study, taking into account sources of potential bias or imprecision. Discuss both direction and magnitude of any potential bias	Discussion ¶ 7
Interpretation	20	Give a cautious overall interpretation of results considering objectives, limitations, multiplicity of analyses, results from similar studies, and other relevant evidence	Discussion ¶ 1-6
Generalisability	21	Discuss the generalisability (external validity) of the study results	Discussion ¶ 7
**Other information**			
Funding	22	Give the source of funding and the role of the funders for the present study and, if applicable, for the original study on which the present article is based	Abstract and Acknowledgements

*Give information separately for exposed and unexposed groups.

Note: An Explanation and Elaboration article discusses each checklist item and gives methodological background and published examples of transparent reporting. The STROBE checklist is best used in conjunction with this article (freely available on the Web sites of PLoS Medicine at http://www.plosmedicine.org/, Annals of Internal Medicine at http://www.annals.org/, and Epidemiology at http://www.epidem.com/). Information on the STROBE Initiative is available at http://www.strobe-statement.org.

References

1. Eilers PHC, Marx BD. Flexible smoothing with B-splines and penalties. *Statistical Science* 1996; **11**(2): 89-121.

2. Todd J, Glynn JR, Marston M, et al. Time from HIV seroconversion to death: a collaborative analysis of eight studies in six low and middle-income countries before highly active antiretroviral therapy. *AIDS* 2007; **21 Suppl 6**: S55-63.

3. Bellan SE, Fiorella KJ, Melesse DY, Getz WM, Williams BG, Dushoff J. Extra-couple HIV transmission in sub-Saharan Africa: a mathematical modelling study of survey data. *Lancet* 2013; **381**(9877): 1561-9.

4. Glynn JR, Ponnighaus J, Crampin AC, et al. The development of the HIV epidemic in Karonga District, Malawi. *AIDS* 2001; **15**(15): 2025-9.

5. White RG, Vynnycky E, Glynn JR, et al. HIV epidemic trend and antiretroviral treatment need in Karonga District, Malawi. *Epidemiol Infect* 2007; **135**(6): 922-32.

6. Camlin CS, Snow RC, Hosegood V. Gendered Patterns of Migration in Rural South Africa. *Popul Space Place* 2014; **20**(6): 528-51.

7. Kishamawe C, Isingo R, Mtenga B, et al. Health & Demographic Surveillance System Profile: The Magu Health and Demographic Surveillance System (Magu HDSS). *Int J Epidemiol* 2015; **44**(6): 1851-61.
